# Case Report: Clinical Responses to Tislelizumab as a First-Line Therapy for Primary Hepatocellular Carcinoma With B-Cell Indolent Lymphoma

**DOI:** 10.3389/fimmu.2021.634559

**Published:** 2021-03-31

**Authors:** Qijun Li, Yong Dong, Yubin Pan, Honglin Tang, Da Li

**Affiliations:** Department of Medical Oncology, Sir Run Run Shaw Hospital, Zhejiang University School of Medicine, Hangzhou, China

**Keywords:** immune checkpoint inhibitors, double primary tumors, hepatocellular carcinoma, B-cell indolent lymphoma, tislelizumab, misdiagnosis, case report

## Abstract

**Background:**

As an emerging therapy with a promising efficacy, immunotherapy has been widely used in the treatment of solid tumors and hematologic malignancies. This clinical study compares the efficacy of tislelizumab, a domestic immune checkpoint inhibitor (ICI), to that of sorafenib when used as a first-line therapeutic option in hepatocellular carcinoma (HCC), and the concurrence of HCC and non-Hodgkin’s lymphoma (NHL) is rare, especially in the treatment of ICIs.

**Case presentation:**

A 61-year-old patient presenting with primary HCC and indolent B-cell lymphoma had a partial clinical response to tislelizumab for his primary HCC. Besides, we described a phenomenon of pseudo-progression and delayed diagnosis of his lymphoma during a long course of treatment.

**Conclusion:**

Tislelizumab, an immunotherapeutic option with a favorable efficacy and toxicity, can be used to manage double primary tumors. However, studies should aim to elucidate the probable mechanisms of this therapy. Pseudo-progression and separation remission make the treatment of double primary tumors even more challenging, which calls for additional caution in patients undergoing immunotherapy to avoid misdiagnosis and, therefore, begin early appropriate interventions.

## Introduction

Globally, liver cancer is the sixth most prevalent malignant tumor, however, it is the second most common cause of tumor associated mortalities (1, 2). Due to its aggressive behavior and limited therapeutic options, hepatocellular carcinoma (HCC), a type of primary liver cancer, has a poor prognosis. Conversely, the nature of indolent non-Hodgkin’s lymphoma (NHL) is relatively mild, despite there being no effectively radical treatment during its long chronic process. Therapeutic resistance, multiple relapses, and biological characteristic transformations lead to poor clinical outcomes (3). Occurrence of double tumors, comprising HCC and NHL, is fairly rare, and treatment is based on individual experience rather than standard protocols. Immunotherapy has rapidly developed and become an efficient therapy for non-small cell lung cancer, malignant melanoma, and other diseases (4, 5). It is a promising option for treating drug-resistant HCC (6). Tislelizumab, a newly humanized IgG4 antibody against programmed cell death-1(PD-1), has been approved for the treatment of Hodgkin’s lymphoma (HL), and a large number of clinical studies, such as the Phase III clinical trial of sorafenib as a first-line treatment for HCC (NCT03412773), have been performed. We report a case of indolent B-cell lymphoma-complicated HCC, which was effectively controlled by tislelizumab as the first line treatment. Authors also reviewed current literature and discussed the possible interaction between HCC and B-cell lymphoma during the long course of treatment.

## Case Presentation

In April 2013, a 61-year-old man was found to have a liver mass by abdominal ultrasonography during regular physical examination. The patient did not exhibit gastrointestinal reactions or abdominal pain symptoms, and he was therefore referred to the Dongyang People’s Hospital for further examination. There was no significant personal or family history that could have aided the diagnosis. During hospitalization, he was diagnosed with chronic hepatitis B (no hepatitis C) with liver cirrhosis, and the level of HBV-DNA was 3.33x10^^5^ IU/ml. The levels of serum tumor markers such as AFP and CEA were found to be 48.32 ng/ml and 5.71 U/ml, respectively. Abdominal computed tomography (CT) revealed a left lateral segment lesion of the liver with multiple enlarged lymph nodes around the lesser peritoneal sac, porta hepatis, retroperitoneum, and right paracardiac regions, which was radiologically suspected for small HCC and lymphadenopathy of infectious etiology. He was subjected to the left lateral lobe hepatectomy and celiac lymphadenectomy for the clinical diagnosis of HCC. Intraoperative findings revealed that the diameter of the lump was 0.9 x 0.8 cm. Pathology indicated hepatocellular carcinoma (**Figure S1**), while the two lymph nodes were negative. Therefore, his final diagnosis was HCC (pT1N0M0 stage I, BCLC 0). The patient was administered with entecavir (0.5 mg) once a day for antiviral treatment. Regular follow-up showed no tumor recurrence or active hepatitis for more than three years (**Table S1**).

**Figure 1:**
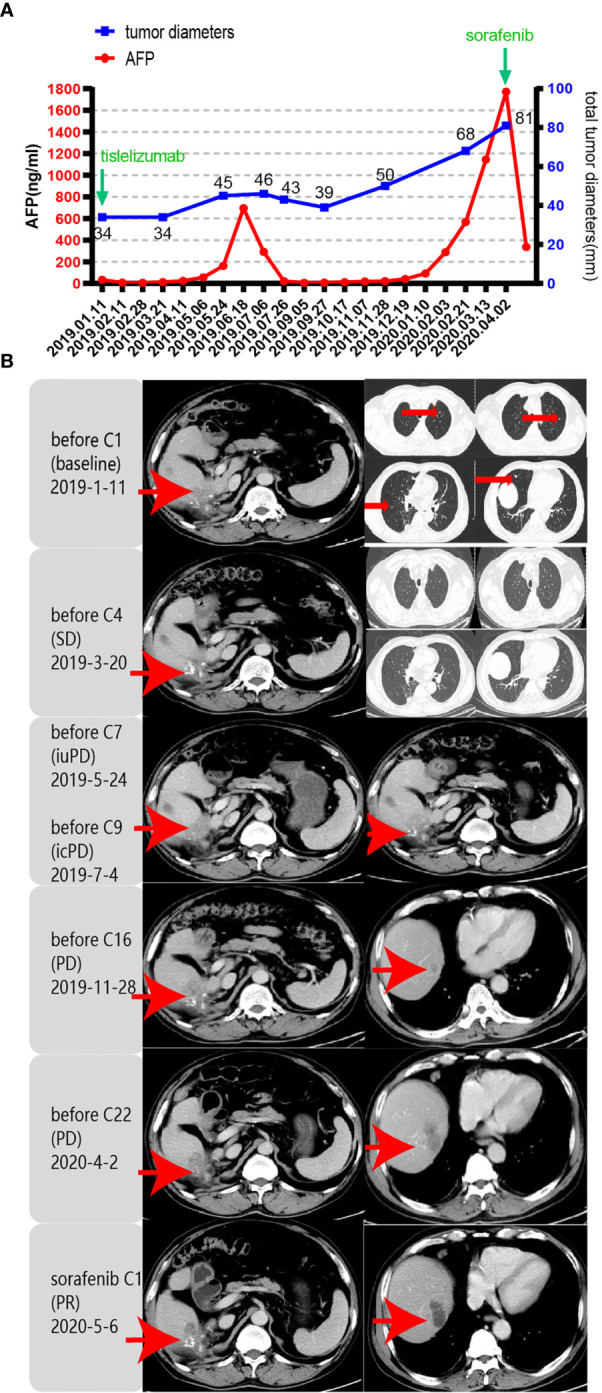
Response evaluation during the clinical course including changes in imaging and quantitative data. iuPD (immunity unconfirmed progressive disease); icPD (immunity confirmed progressive disease). **(A)** Trends in the levels of tumor monitoring indicators, including AFP (left Y-axis) and tumor diameters (right Y-axis) corresponding to the treatment timeline. X-axis showing the date of the disease course. The frequency of imaging evaluations is less than that of AFP. **(B)** Representative images of the CT scan revealed the increasing and decreasing process of both primary and metastatic lesions in the liver and lung after PD-1 antibody (tislelizumab) and sorafenib treatment. Red arrows indicate tumor lesions.

**Figure 2:**
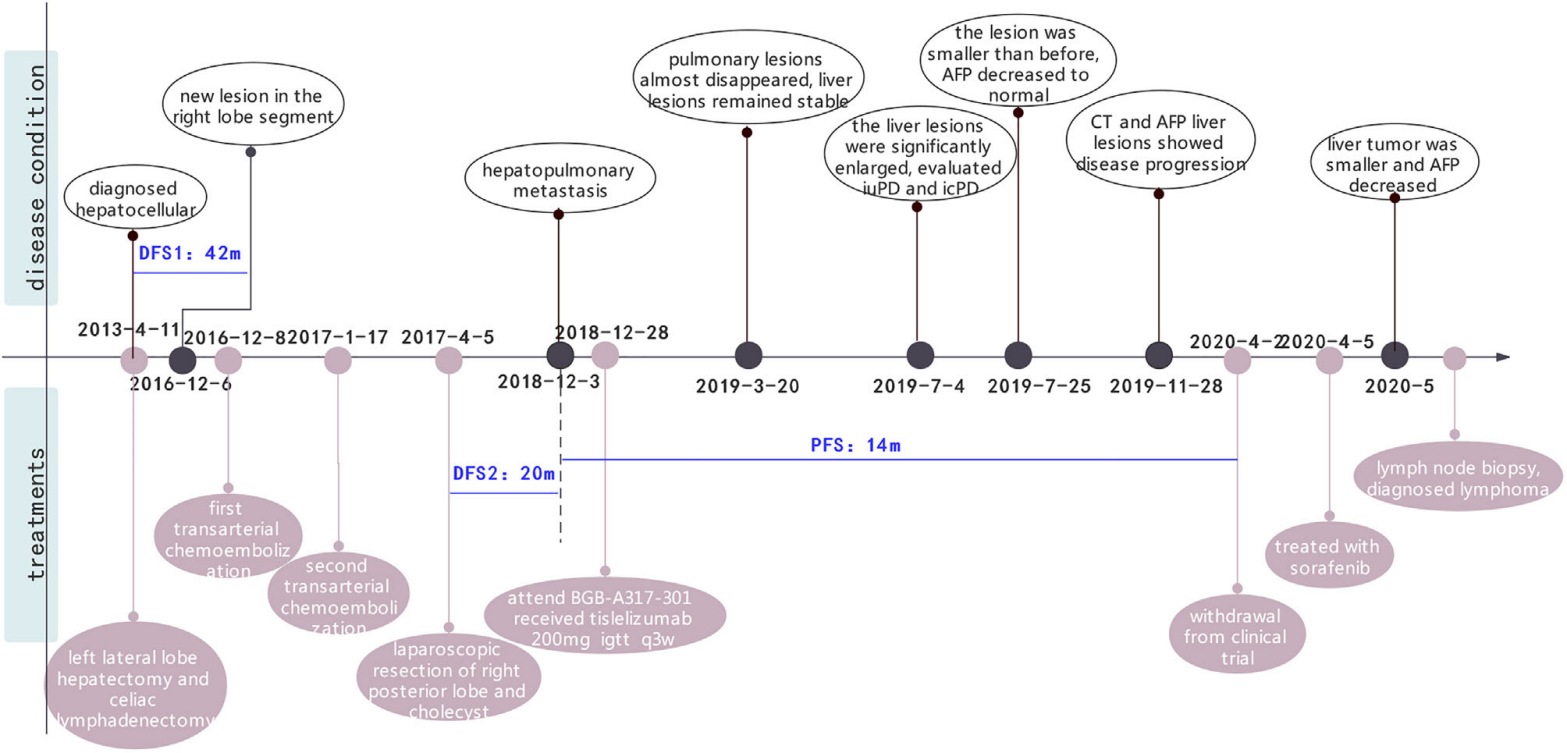
The whole clinical timeline of the patient, with major treatment and disease status. DFS, disease-free survival; PFS, progression-free survival.

Unfortunately, in July 2016, a new lesion in the right lobe segment was discovered by abdominal CT, without abnormal levels of serum liver function and tumor markers. However, the patient declined treatment with western medicines. In December 2016, the CT scan showed enlarged multiple liver nodules with vascular involvement, and he was hospitalized with HCC (cT3bN0M0 stage IIIB, BCLC B) in our hospital. Transarterial chemoembolization (TACE) was successively performed twice to downstage the tumor. After neoadjuvant therapy, he was histologically reassessed as HCC (ypT3N0M0 stage IIIB, BCLC B). Then, laparoscopic R0 resection of the right posterior liver lobe as well as the gall bladder was performed in April 2017 to eliminate residue lesions. Intraoperative findings revealed that lesion diameters were 9x8.5x10 cm with negative surgical margins. After partial liver resection, the patient’s HBV-DNA copies increased to 2.35x10^^4^ IU/ml, with mildly abnormal AST, ALT, and bilirubin levels. He was continuously administered with antiviral therapies and other medicines to protect liver functions. After successful management of the disease, he was discharged and regular follow-ups were performed thereafter. In December 2018, the patient was found to have hepatopulmonary metastases. CT scans showed multiple small nodules, the largest of which (in the lung) was about 8.6x6 mm, most possibly a result of HCC metastases, and about 18 mm in the liver. The patient was clinically diagnosed with HCC (cT3N0M1 stage IV, BCLC C). Notably, these enlarged lymph nodes in the peritoneum remained the same as before. Systemic therapies are available to treat patients with unresectable HCC. Multitargeted tyrosine kinase inhibitors (TKIs), such as sorafenib or lenvatinib, are first-line systemic therapies. The patient had not had a previous prescription of systemic treatments, and therefore, he met the inclusion criteria for BGB-A317-301(NCT03412773), a global study designed to compare the clinical efficacy as well as safety of tislelizumab and sorafenib (1:1 randomized) as a first-line systemic treatment for unresectable HCC, which was considered beneficially to the patient (clinical trial protocols are provided in the supplementary materials). After signing the informed consent, the patient was serially screened for confounding factors (exclusion criteria). On January 18^th^ 2019, he was administered with tislelizumab 200 mg igtt q3w. To accurately evaluate immunotherapeutic efficacy, we used iRECIST to determine disease progression (7). Reductions in pseudo-progressions (PsPDs) during immunotherapy were recorded. During two cycles of tislelizumab, the patient developed multiple rashes with pruritus (grade 2, CTCAE 5.0), and on February 1^st^ 2019, he was treated with loratadine, ebastin, and mometasone ointment. Since the rashes got worse (grade 3, CTCAE 5.0), the third cycle of therapy was suspended. From February 25^th^ 2019, the patient was orally administered with 35 mg prednisolone tablet once a day, and the rash was gradually alleviated. Dosage of prednisolone was reduced stepwise by the order of 25 mg, 15 mg, and 10 mg, with the last dose on March 21^st^, 2019. Before the third immunotherapeutic cycle, CT scans indicated that pulmonary lesions almost disappeared while the liver lesions remained stable (**Figure 1B**). Moreover, before the seventh cycle, the liver lesions were significantly enlarged, without a corresponding enlargement of lymph nodes, evaluated as iuPD (immunity unconfirmed progressive disease). In the ninth cycle, lesions were similar to the seventh cycle and evaluated as icPD (immunity confirmed progressive disease). During the treatment, AFP reached the highest level (696.18 ng/ml) before the eighth treatment (**Figure 1A**). Given that the patient did not meet the exclusion criteria, it was considered that he could still benefit from tislelizumab, and therefore, his participation in the clinical trial was continued after deep consideration. CT scan before the tenth cycle showed that the lesion size was smaller than the last assessment, and simultaneously, AFP decreased to normal (21.14 ng/mL). Therefore, the researchers considered that the patient exhibited “false progression” in the previous treatment. Liver lesions remained stable until the thirteenth cycle. Before the sixteenth cycle, there was slow disease progression, while before the 22^nd^ cycle (2020–4–2), liver lesions exhibited significant progression. Therefore, it was considered that tislelizumab was no longer beneficial to the patient, and he was withdrawn from the clinical trial. Disease progression is shown in **Figure 2**, and except for the maculo-papule, all adverse events are described in **Table 1**.

**Table 1:** Adverse events in the tislelizumab therapeutic course in the patient (graded by CTCAE 5.0).

Adverse events	Baseline	Maximum grade	Duration	irAE	Treatment
Pruritus	0	II	C2-C3	Yes	Glucocorticoid
Rash maculopapule	0	III	C2-C3	Yes	Glucocorticoid
Leukocytosis	0	III	C2-C5, C18-C21	No, may be unrelated	No
Lymphocyte count increased	I	III	C2-C5, C18-C21	No, may be related	No
Alanine transaminase (ALT) levels increased	I	I	C8	May be related	Medicine
Aspartate aminotransferase (AST) levels increased	0	I	C8	May be related	Medicine
Blood bilirubin levels increased	0	I	C14-C15	May be related	Medicine

The first screening indicators after the clinical trial as the baseline; CTCAE, Common Terminology Criteria for Adverse Events; C, cycle; irAE, immune-related adverse events.

## Outcomes and Follow-up

**Figure 3:**
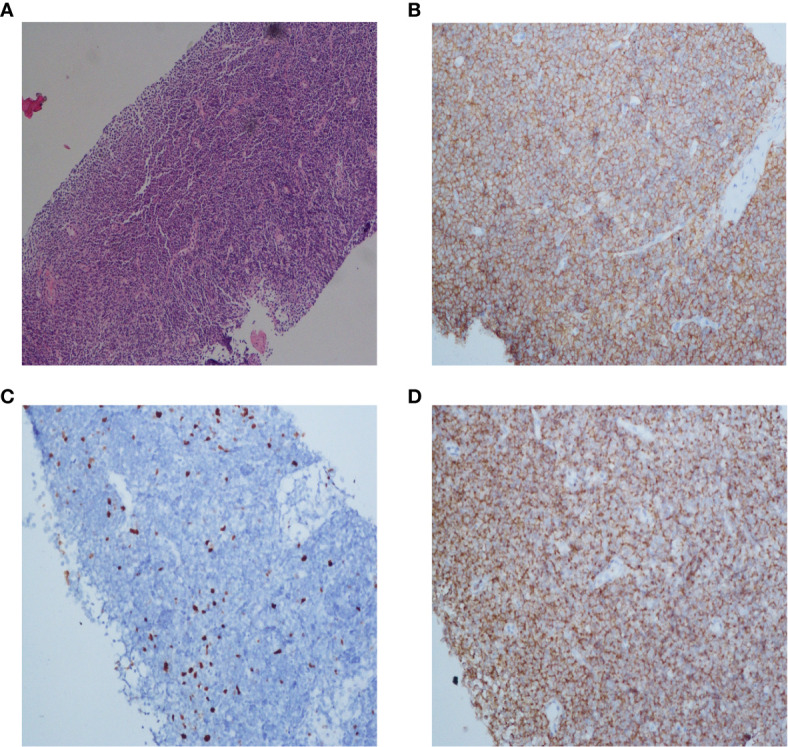
Histopathology and immunohistochemistry (IHC) of the lymph node of this patient. Microscopic observation (10×) of H&E staining showed a dense diffuse lymphoid cells infiltration **(A)**. Immunohistochemical staining of CD20 and Bcl-2 expression (20×) showed that tumor cells were positive for CD20 and Bcl-2, respectively **(B, D)**. The Ki-67 proliferative index (20×) was low **(C)**.

After being discontinued from the clinical trial, the patient was treated with sorafenib. One month later, the liver tumor was found to be smaller and AFP decreased significantly. Meanwhile, the care team noticed that during treatment with tislelizumab, his lymph nodes did not exhibit any change, regardless the lesions were either enlarged or shrank, HBV was either well or poorly controlled. For further evaluation, we performed a lymph node biopsy. Histological examinations of axillary and inguinal lymph nodes revealed indolent B-cell lymphoma (**Figure 3**). Retrospectively analyzed, his peritoneal sac, paracardiac regions, and superficial lymph nodes remained the same in size and unparallel to tumor progression, which supported that the patient had lymphoma seven years ago. It is unfortunate that a lymph node biopsy was not performed at that time, therefore, there is no definite pathological evidence to support our hypothesis. Currently, clinical follow-ups are still being performed and to date, the patient continues receiving sorafenib treatment and survives his double tumors. Under the treatment of ICIs, despite signs of “false progression”, his disease course showed his liver lesions were responsive to tislelizumab overall, but not his lymphoma. Recently, the patient was discovered to have an elevated lymphocyte count but without clinical symptoms, which may be correlated with the indolent lymphoma. Hematological assessments are shown in the Supplementary material (**Figure S2**).

## Discussion

Warren’s definition of multiple primary tumors refers to the simultaneous or successive occurrence of two or more unrelated primary malignant tumors (8, 9). Patients with malignant tumors are more likely to develop a second malignant tumor, which may be due to the persistent effects of risk factors, radiotherapy, and chemotherapy (10). Currently, incidences of synchronous multiple primary cancer are increasing. Carson H. J. documented the reported cases of synchronous NHL with other cancers. The most common are colon cancer, prostate cancer, and lung cancer respectively, while the HCC, is penultimate (11). In case reports of HCC with lymphoma (**Table 2**), the most common is HCC with invasive diffuse large B-cell lymphoma (DLBCL), while follicular lymphoma (FL) represents the majority of indolent NHL, which is consistent with the incidence of NHL (33). The overall survival (OS) of indolent NHL is high, with 70% of patients having more than 10 years of survival. However, in multiple malignant tumors, interactions between tumors may affect the OS. Retrospective studies have shown that the prognosis of gastric cancer patients complicated with lymphoma may depend more on gastric cancer (34), but Lee SI et al. documented that in patients with both HCC and hepatitis, delayed diagnosis of NHL, especially DLBCL, is associated with a poor prognosis (29). Therefore, it is crucial to identify multiple primary tumors early and precisely.

**Table 2:** Clinical information of hepatocellular carcinoma with lymphoma patients.

Case	Age (year)	Gender	HBV/HCV	Lymphoma		Hepatocellular adenocarcinoma		OS (months)
				Diagnosis	Treatment	Staging	Treatment	
Talamo T (12)	67	male	HBV	malignant lymphoma	palliative	IV stage	palliative	<1
Cavanna L (13)	50	male	Neither	NHL	chemotherapy	pT1NxM0	operation	30
Ono T (14)	59	female	HCV	DLBCL	None	T1NxM0	TACE	18
Shikuwa S (15)	64	male	HBV	B cell	None	TxNxM1	chemotherapy and radiotherapy	11
Monarca R (16)	66	male	HBV	chronic and indolent B-cell	Not mentioned	T1N0M0	Not mentioned	Not mentioned
Suriawinata A (17)	55	male	HCV	DLBCL	None	T2NxM0	liver transplantation	>15
Shapira M (18)	70	male	HCV	DLBCL	/	T2NxM0	/	/
Takeshima F (19)	65	female	HBV	MALT	hepatic segmentectomy	pT1N0M0	hepatic segmentectomy	>10
Kataoka T (20)	64	male	Neither	DLBCL	conservative therapy	T1NxM0	conservative therapy	1.5
Othsubo K (21)	66	male	HCV	DLBCL	R-CHOP	T2NxM0	RFA	Not mentioned
Himoto T (22)	63	male	HCV	DLBCL	CHOP	T1N0M0	PEIT and RFA	Not mentioned
Nonami A (23)	73	male	HBV	DLBCL	R-CHOP	T2N0M0	hepatectomy	>22
Lin A (24)	70	male	HCV	DLBCL	R-CHOP	Not mentioned	Not mentioned	>5
Lin A (24)	65	female	HCV	MZL	R-CHOP and radiotherapy	T2NxMx	None	14
Utsunomiya T (25)	70	female	HCV	DLBCL	Not mentioned	pT2N0M0	partial hepatectomy	4
Becker D (26)	68	male	HCV	SLL/CLL	untreated	pT1N0M0	RFA	Not mentioned
Heidecke S (27)	70	male	Neither	CLL	untreated	pT2NxMx	operation	>17
Tajiri H (28)	75	male	HCV	DLBCL	R-THP-COP	pT1N0M0	hepatectomy and chemotherapy	>12
Lee S (29)	60	male	HCV	FL	R-CVP	T2NxM0	TACE and RFA	>20
Chan R (30)	59	male	HBV	MALT	Right hepatectomy	pT1N0M0	Right hepatectomy	>48
Lee M (31)	52	male	HBV	MCL	CHOP	T3N0M0	RFA	>12
Meng J (32)	58	male	HBV	DLBCL	CHOP	I stage	operation	>62

HBV, chronic hepatitis B; HCV, chronic hepatitis B; NHL, non-Hodgkin’s lymphoma; DLBCL, diffuse large B-cell lymphoma; MALT, mantle cell lymphoma; SLL, small lymphocyte lymphoma; CLL, chronic lymphocytic leukemia; FL, follicular lymphoma; MZL, marginal zone lymphoma; MCL, mantle cell lymphoma; R-CHOP, a combination of rituximab, cyclophosphamide, adriamycin, vincristine and prednisone; R-THP-COP, a combination of rituximab, pirarubicin, cyclophosphamide, vincristine, and prednisolone; R-CVP, a combination of rituximab, cyclophosphamide, vincristine and prednisone; TACE, transarterial chemoembolization; RFA, radiofrequency ablation; PEIT, percutaneous ethanol injection therapy.

In our case, the patient with HCC was not diagnosed with B-cell indolent lymphoma until the lymph node biopsy was implemented at the later stage, so we made a retrospective analysis. Occurrence of both HCC and chronic hepatitis affects the diagnosis of multiple-lymphadenopathy, which is easily misdiagnosed as “reactive hyperplasia” or “lymph node metastasis of HCC”. Misdiagnoses resulted in delayed treatments. Therefore, NHL should be regarded as a differential diagnosis for HCC and chronic hepatitis patients (29). Compared to metachronous neoplasms, synchronous multiple neoplasms are perhaps tougher to identify. Next-generation sequencing (NGS) is effective in the identification of tumor-specific genes, which is important in the diagnosis of multifocal tumors and in informing clinical treatments (35). In our case, swollen lymph nodes were found in the initial treatment of HCC and were not parallel to the changes in HBV-DNA or tumor development. Therefore, if there is “separation remission” during the clinical treatment process, the possibility of double primary tumors should be ruled out. In our case, lymph node biopsy was negative for lymph node metastasis of liver cancer, so in the status of liver tumor progression with lung metastasis disappearance, local treatments of the liver can also be considered in the following treatments.

As mentioned in the treatment strategies, HCC is often diagnosed in the advanced stage, therefore, systemic treatment plays an essential role in its control. However, as the first-line treatment for advanced HCC, sorafenib does not show a dramatic benefit. The median survival time of sorafenib was only three months longer than placebo (36). Advances in immunotherapy have enhanced tumor treatment (5, 37), and some have shown good therapeutic effects in HCC. A phase III trial involving a combination of atezolizumab and bevacizumab as first-line treatment for unresectable HCC revealed a significant improvement, with a 12-month prolonged OS compared to sorafenib (38). Phase III randomized controlled trials comparing nivolumab (39) and tislelizumab (NCT03412773) with sorafenib as first-line therapeutic options for advanced HCC have been launched in succession. Unlike solid tumors, the decisive prognostic factor for patients with lymphoma is the pathological type rather than clinical stage. Even though the clinical course of indolent lymphoma is always stable or spontaneously relieved before progression (40), it is mostly incurable and has the probability of transforming to invasive lymphoma such as DLBCL (41), especially under the circumstance of immune disorders in patients with active tumor. Therefore, indolent lymphoma might require the same aggressive treatments under those scenarios. R-CHOP (a scheme including rituximab, which is an anti-CD20 monoclonal antibody, mAbs) is the first-line recommended treatment for indolent lymphoma at stages III and IV. In refractory HL, ICIs have shown good clinical outcomes (42). There are no clinical trials of tislelizumab for NHL, however, ICIs such as nivolumab (NCT02038946) have been used in the treatment of NHL, some case reports and small sample research studies of certain NHLs have displayed durable response under the treatment of ICIs (43, 44). At molecular and cytological levels, PD-1 positive expression was detected in DLBCL, FL and marginal zone lymphoma (MZL) (45). The density of PD-1 positive cells in FL is associated with the prognosis and possibility of transformation to DLBCL. However, expression levels of PD-1 vary from different studies (45–47). Most FLs have been shown to have a stronger immune escape (48), which may be due to the rich PD1^+^ γδ T lymphocytes. PD-1 regulates the immune components of γδ T cytotoxic cells, resulting in the hypofunction of γδ T lymphocytes which reduces antibody-dependent cell-mediated cytotoxicity (ADCC) (49). Therefore, ICIs have the ability to slow down FL development. Anti-CD20 mAbs can enhance intratumoral infiltration of γδ T cells (50), which provides the possibility to improve the efficacy of ICIs against immune desert tumors by rituximab. It is theoretically proven that a combination or bispecific antibodies of anti-CD20 mAbs and ICIs in the treatment of NHL complicated HCC can enhance the therapeutic effect.

Moreover, both NHL and HCC are associated with hepatitis B or C viruses (51, 52), and NHL patients have higher odds for HCC development (53, 54). Activated NF-κB pathways have been simultaneously reported in a mantle cell lymphoma (MCL), renal cell carcinoma and stromal tumor tissue (55), suggesting that there may be a common pathway between lymphoma and solid tumors. Similarly, through different mechanisms such as cell proliferation and oxidative stress, Bccip, miR-29, and PI3K pathways can simultaneously induce NHL and HCC (56–59). Therefore, the correlation between NHL and HCC theoretically implies that multiple primary tumors of NHL and HCC have a higher incidence rate. Moreover, immunotherapy makes it possible to manage related multiple primary tumors at the same time.

Even though we adopted tislelizumab instead of the standard first-line treatment of sorafenib when we treated the disease as primary HCC initially, treatment indications and the patient’s views were fully considered. The clinical trial provided the patient more treatment options, and the patient truly had clinical benefits from tislelizumab. Immunotherapy for HCC is promising and may also benefit indolent NHL patients that are resistant to chemotherapy. In our case, while lymphoma did not respond to tislelizumab, the influence of tislelizumab in NHL progression and histological transformation cannot be negated. The patient had no significant progression in lymph nodes and hematological indicators during treatment of tislelizumab, however, outcomes were different during sorafenib treatment. The natural course of NHL could not be excluded. Hematological tumors are often caused by multiple co-inhibitory signaling pathways (60), which suppress immune activation. Compared to chemotherapy, rituximab has been shown to reduce the risk of histological transformation in indolent lymphoma (2, 61). Treatments for asymptomatic indolent lymphoma are unnecessary, however reducing incidences of histological transformation in double or multiple primary tumors with indolent lymphoma may be a consideration for treatment options.

One of drawbacks of this case is the failure to identify lymphoma at the early stage, which made accurate parallel analysis along the treatment course impossible. In addition, detection of immune treatment resistance of HCC, such as the accumulation of β-catenin, was not completed in the later stage, and lacked the underlying relevant mechanism.

## Conclusion

There are no standard therapeutic options for lymphoma and HCC, as well as for double primary tumors. Individualized treatments should be decided based on the behavior of tumor and patients’ overall conditions. Clinical management of HCC with indolent B-cell lymphoma has rarely been reported in previous studies. Among the described cases, most are early HCC with local therapy, while standard therapy is used to treat lymphoma. To our knowledge, there are no reports of advanced HCC complicated with lymphoma using systemic therapy and achieves a long survival in literature.

For double primary tumors, latent cancer may be misdiagnosed as the progression of the first primary tumor due to primary drug resistance of ICIs (62). Therefore, it is necessary to determine the pathology of abnormal lymph nodes through biopsy. NHL can infiltrate into other tumors, such as HCC and renal cell carcinoma, so when atypical lymphoid cells are detected in tumor tissues, attention should be paid to mixed pathological features (25) since diagnosis of a double tumor will substantially affect clinical management. Moreover, NHL invasion may lead to the occurrence of precancerous lesions and promote the development of HCC (12, 20). Therefore, early diagnosis of lymphoma and simultaneous treatment of lymphoma and HCC can theoretically prolong survival time. Pathological characteristics for HCC and NHL are different, therefore, when standard systemic therapy for both tumors is applied synchronously, there may be great toxicity and poor tolerability. Treatment of HCC in the remission stage of NHL may improve the cure rate. This study elucidates the correlation between NHL and HCC, and the necessity for treating both diseases at the same time. The synergistic effects of CD20 mAbs and ICIs might provide a potential therapeutic option for double primary tumors with NHL. However, this conclusion should be verified further.

## Data Availability Statement

The original contributions presented in the study are included in the article/**Supplementary Material**. Further inquiries can be directed to the corresponding author.

## Ethics Statement

The studies involving human participants were reviewed and approved by Sir Run Run Shaw Hospital, Zhejiang University School of Medicine. The patients/participants provided their written informed consent to participate in this study. Written informed consent was obtained from the individual(s) for the publication of any potentially identifiable images or data included in this article.

## Author Contributions

All authors have contributed to the preparation of this manuscript. All authors contributed to the article and approved the submitted version.

## Funding

This work was financially supported by the National Natural Science Foundation of China (81573003), the Association Foundation of Zhejiang Natural Science Foundation-Zhejiang Society for Mathematical Medicine (NO. LSY19H160005), CSCO (Chinese society of clinical oncology) research foundation (Y-XD2019-243), and CSCO research foundation (Y-Roche2019/2-0042).

## Conflict of Interest

The authors declare that the research was conducted in the absence of any commercial or financial relationships that could be construed as a potential conflict of interest.
