# Supplementary material for: Case Report: Clinical Responses to Tislelizumab as a First-Line Therapy for Primary Hepatocellular Carcinoma With B-Cell Indolent Lymphoma
Source: Front Immunol. 2021 Mar 31;12:634559. doi: 10.3389/fimmu.2021.634559 (PMC8044442; doi:10.3389/fimmu.2021.634559)
Supplement: Supplementary file 1 [file Presentation_1.zip › 634559_SupMaterial/CLINICAL RESEARCH PROTOCOL.PDF]

CLINICAL RESEARCH PROTOCOL

**Protocol Title:**	A Randomized, Open-label, Multicenter Phase 3 Study to Compare the Efficacy and Safety of BGB-A317 versus Sorafenib as First-Line Treatment in Patients with Unresectable Hepatocellular Carcinoma
**Protocol Identifier:**	BGB-A317-301
**Phase:**	3
**Investigational Product:**	Tislelizumab (BGB-A317)
**Reference Number**	EudraCT 2017-002423-19
**Indication:**	Unresectable Hepatocellular Carcinoma
**Sponsor:**	BeiGene, Ltd.<br>c/o BeiGene USA, Inc.<br>1900 Powell Street<br>Suite 820<br>Emeryville, CA 94608<br>USA
**Sponsor Medical Monitors:**	Frederic Boisserie, MSc<br>55 Challenger Road, Suite 501<br>Ridgefield Park 07660 USA<br>Phone number: +1 857-327-8432<br>Email: frederic.boisserie@beigene.com<br><br>Yaxi Chen, MD<br>22nd Floor, Tower D<br>Central International Trade Center<br>No.6A Jianguomenwai Avenue<br>Chaoyang District 100022<br>Beijing, China<br>Phone number: +86 18501361479<br>Email: yaxi.chen@beigene.com
**Original Date:**	05 July 2017
**Amendment No. (Date):**	1.0 (13 September 2017)
**Amendment No. (Date)**	2.0 (03 October 2017)
**Amendment No. (Date)**	3.0 (18 October 2017)
**Amendment No. (Date)**	4.0 (28 June 2018)
**Amendment No. (Date)**	5.0 (11 May 2020)

Confidentiality Statement

This Document Is Not for Distribution – Do Not Copy

This document contains confidential information and is the proprietary property of BeiGene, Ltd., and its subsidiaries. This document is for use by individuals and their designated representatives for their confidential review, consideration, and/or participation in investigational trial(s). This document may not be copied or distributed for review by any unauthorized individuals without the prior written authorization of BeiGene, Ltd., or one of its subsidiaries. Your acceptance of this document constitutes agreement that you will not disclose the information contained herein to others without the prior written authorization from BeiGene, Ltd., or one of its subsidiaries.

FINAL PROTOCOL APPROVAL SHEET

Protocol BGB-A317-301: A Randomized, Open-label, Multicenter Phase 3 Study to Compare the Efficacy and Safety of BGB-A317 versus Sorafenib as First-Line Treatment in Patients with Unresectable Hepatocellular Carcinoma

BeiGene, Ltd., Approval:

11-MAY-2020

Frederic Boisserie, MSc
Director, Clinical Science

Date

INVESTIGATOR SIGNATURE PAGE

Protocol Title: A Randomized, Open-label, Multicenter Phase 3 Study to Compare the Efficacy and Safety of BGB-A317 versus Sorafenib as First-Line Treatment in Patients with Unresectable Hepatocellular Carcinoma

Protocol Identifier: BGB-A317-301

This protocol is a confidential communication of BeiGene, Ltd., and its subsidiaries. I confirm that I have read this protocol, I understand it, and I will work according to this protocol and the terms of the clinical study agreement governing the study. I will also work consistently with the ethical principles that have their origin in the Declaration of Helsinki and that are consistent with good clinical practices and the applicable laws and regulations. Acceptance of this document constitutes my agreement that no unpublished information contained herein will be published or disclosed without prior written approval from BeiGene, Ltd., or one of its subsidiaries.

Instructions for Investigator: Please SIGN and DATE this signature page prior to implementation of this sponsor-approved protocol. PRINT your name, title, and the name of the center in which the study will be conducted.

I have read the protocol in its entirety and agree to carry out the study according to this protocol.

Signature of Investigator: ______________________________ Date:______________
Printed Name: ______________________________
Investigator Title ______________________________
Name/Address of Center ______________________________
____
____
____

SYNOPSIS

**Name of Sponsor/Company:** BeiGene, Ltd.
**Investigational Product:** Tislelizumab (BGB-A317)
**Title of Study:** A Randomized, Open-label, Multicenter Phase 3 Study to Compare the Efficacy and Safety of BGB-A317 versus Sorafenib as First-Line Treatment in Patients with Unresectable Hepatocellular Carcinoma
**Protocol Identifier:** BGB-A317-301
**Phase of Development:** 3
**Number of Patients:** Approximately 640
**Study Centers:** Approximately 100 centers internationally
**Study Objectives:**<br>Primary:<br>• To compare overall survival (OS) between tislelizumab and sorafenib as first-line treatment in patients with unresectable hepatocellular carcinoma (HCC)<br>Secondary:<br>• To compare objective response rate (ORR) assessed by Blinded Independent Review Committee (BIRC) according to Response Evaluation Criteria in Solid Tumors (RECIST) Version (v)1.1 between tislelizumab and sorafenib<br>• To compare progression-free survival (PFS) assessed by BIRC according to RECIST v1.1 between tislelizumab and sorafenib<br>• To compare duration of response (DOR) assessed by BIRC according to RECIST v1.1 between tislelizumab and sorafenib<br>• To compare time to progression (TTP) between tislelizumab and sorafenib<br>• To compare health-related quality of life (HRQoL) between tislelizumab and sorafenib<br>• To compare tumor assessment outcomes (ie, ORR, PFS, DOR, TTP) assessed by the investigator according to RECIST v1.1 between tislelizumab and sorafenib<br>• To compare disease control rate (DCR) and clinical benefit rate (CBR), assessed by BIRC and investigator according to RECIST v1.1, between tislelizumab and sorafenib<br>• To compare safety and tolerability of tislelizumab versus sorafenib<br>Exploratory:<br>• To explore potential predictive biomarkers, including but not limited to programmed cell death ligand-1 (PD-L1) expression<br>• To characterize the pharmacokinetics (PK) of tislelizumab in patients with unresectable HCC<br>• To determine host immunogenicity to tislelizumab in patients with unresectable HCC

Study Endpoints:

Primary:

- OS

Secondary:

- ORR by BIRC
- PFS by BIRC
- DOR by BIRC
- TTP by BIRC
- HRQoL
- Tumor assessments (ORR, PFS, DOR, and TTP) assessed by investigator per RECIST v1.1
- DCR by BIRC and investigator
- CBR by BIRC and investigator
- Safety assessment (eg, new adverse events [AEs], AEs present at baseline that worsen in severity during the study, and clinical laboratory abnormalities)

Exploratory:

- PD-L1 expression and other potential predictive biomarkers
- Summary of serum concentrations of tislelizumab
- Assessments of immunogenicity of tislelizumab by determining the incidence of antidrug antibodies (ADAs)

Study Design:

This is a randomized, open-label, multicenter Phase 3 study to compare the efficacy and safety of tislelizumab to that of sorafenib as first-line treatment in adult patients with unresectable HCC. The study design schema is as follows:

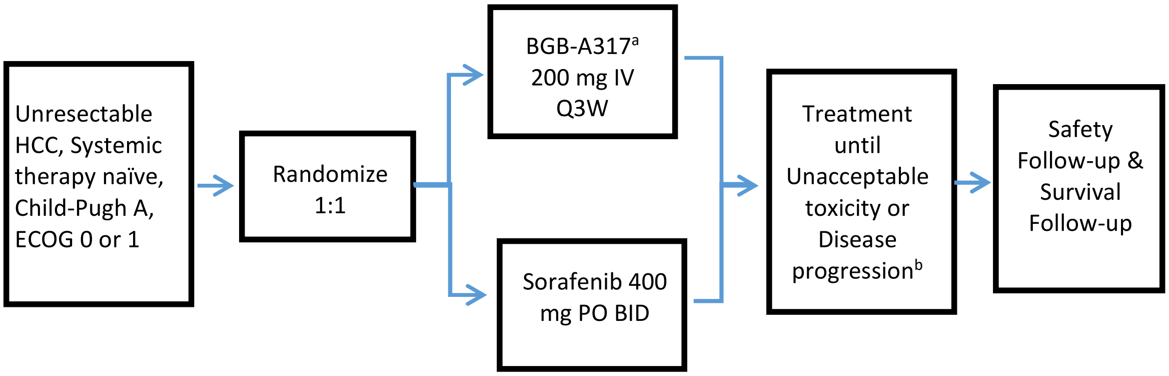

a. The initial infusion (Cycle 1, Day 1) will be administered over a period of 60 minutes. If this infusion is well tolerated, subsequent infusions may be administered over 30 minutes. After tislelizumab infusion, patients will be further monitored for a period of 2 hours during Cycles 1 and 2. From Cycle 3 onward, a postinfusion monitoring period of ≥ 30 minutes will be required.

b. At the discretion of the investigator, patients may be treated beyond disease progression under protocol-defined conditions (Section 7.13.1).

Abbreviations: BID, twice daily; ECOG, Eastern Cooperative Oncology Group; HCC, hepatocellular carcinoma; IV, intravenously; PO, orally; Q3W, once every 3 week

Before initiating this Phase 3 study in Japan, a substudy investigating the safety, tolerability, PK and preliminary efficacy in Japanese patients is planned (see Appendix 13 for details).

After providing written informed consent, completing all Screening assessments, and being confirmed as eligible for study participation, approximately 640 patients will be randomized in a 1:1 ratio to receive (on an open-label basis) either tislelizumab or sorafenib.

At randomization, patients will be stratified by the following 5 factors:

- Macrovascular invasion (present vs absent)
- Extrahepatic spread (present vs absent)
- ECOG (0 vs 1)
- Etiology (hepatitis C virus [HCV] vs other [includes hepatitis B virus (HBV)])
- Geography (Asia [excluding Japan] vs Japan vs Rest of World)

Patients with HBV and HCV co-infection will be grouped along with HBV into the "other" category of etiology for randomization. Patients will then begin open-label treatment with 1 of the following regimens:

- Arm A: tislelizumab 200 mg intravenously (IV) once every 3 weeks (Q3W)
- Arm B: Sorafenib 400 mg orally (PO) twice daily (BID)

All study treatment is to be continually administered until intolerable toxicity, withdrawal of informed consent, or the time point at which, in the opinion of the investigator, the patient is no longer benefiting from study therapy

Treatment beyond the initial investigator-assessed, RECIST v1.1-defined disease progression is permitted in both treatment arms provided the patient meets the criteria described in in Section 7.13.1.

Study Assessments:

Tumor response will be evaluated by the BIRC and investigator every 9 weeks during Year 1 and every 12 weeks from Year 2 onwards, in accordance with RECIST v1.1. If a patient discontinues study treatment due to any reason other than disease progression or death, tumor assessments will continue as scheduled until, disease progression, death, loss to follow-up, withdrawal of consent, or until the study terminates, whichever occurs first.

Patients will be evaluated for any AEs and serious adverse events (SAEs) occurring up to 30 days after the last dose of study drug (all severity grades, per National Cancer Institute Common Terminology Criteria for Adverse Events [NCI-CTCAE] Version [v]4.03) or initiation of new anticancer therapy, whichever occurs first, and all immune-related adverse events (irAEs) (tislelizumab arm only) occurring up to 90 days after the last dose of tislelizumab, regardless of whether or not the patient starts a new anticancer therapy. All drug-related SAEs will be recorded by the investigator after treatment discontinuation until patient death or loss to follow-up, whichever occurs first.

Safety and efficacy monitoring will be performed by an Independent Data Monitoring Committee (IDMC). The IDMC may recommend modifications to the study, including termination due to safety and/or efficacy concerns. The functions and membership of the IDMC will be described in the IDMC Charter.

Key Eligibility Criteria:

The population under study is adult patients (≥ 18 years of age on the day the patient voluntarily agrees to participate in the study) with histologically confirmed HCC that is classified as Barcelona Clinic Liver Cancer (BCLC) Stage C disease or BCLC Stage B disease that is not amenable to or has progressed after loco-regional therapy, and is not amenable to a curative treatment approach, and has not been previously treated with systemic therapy (in keeping with the first-line setting of this study). All patients are also required to have a Child-Pugh A classification for liver function assessed within 7 days of randomization, ≥ 1 measurable lesion per RECIST v1.1, and an Eastern Cooperative Oncology Group Performance Status score of ≤ 1.

Test Product, Dose, and Mode of Administration:

Tislelizumab will be administered at a dose of 200 mg IV Q3W.

Reference Therapy, Dose, and Mode of Administration:

Sorafenib will be administered at a dose of 400 mg PO BID.

Statistical Methods:

The statistical methods described in this section cover the summary of data collected from all randomized patients in the study.

Overall survival is the primary endpoint of the study. No multiplicity adjustments will be needed for testing both noninferiority or superiority on OS, due to the closed testing principle.

The secondary efficacy endpoints will be tested according to their order of presentation in Section 2.2.2. Treatment arm comparison for the key secondary endpoint ORR and for the other secondary endpoints will be performed if noninferiority for the primary efficacy endpoint (OS) is declared. Testing will continue until the first nonsignificant outcome occurs.

All tests will be performed at one-sided $\alpha = 0.025$ (or 2-sided $\alpha = 0.05$), unless otherwise specified.

Analysis Sets:

- Intent-to-Treat (ITT) Analysis Set – Includes all randomized patients. Patients will be analyzed according to their randomized treatment arm (ie, either tislelizumab or sorafenib). This will be the primary analysis set for all efficacy analyses.
- Per-Protocol (PP) Analysis Set – Includes all randomized patients who received ≥ 1 dose of their assigned study drug (tislelizumab or sorafenib) and had no major protocol deviations. Major protocol deviations will be determined and documented before the database lock for the primary analysis.
- Safety Analysis Set – Includes all patients who received ≥ 1 dose of their assigned study drug. (tislelizumab or sorafenib). This will be the analysis set for all safety analyses.

Primary Efficacy Analyses:

Overall Survival

The OS will be compared between the tislelizumab arm (Arm A) and the sorafenib arm (Arm B) by testing the null hypothesis of noninferiority, as follows:

H_0: $HR_{A/B} \geq 1.08$ against the alternative hypothesis of noninferiority;

H_a: $HR_{A/B} < 1.08$, where $HR_{A/B}$ denotes the true hazard ratio (HR) for tislelizumab versus sorafenib. A one-sided type 1 error of 0.025 will be used in the test of OS.

The 95% confidence interval (CI) of $HR_{A/B}$ will be estimated in the ITT analysis set using a Cox proportional hazard model with treatment arm as a factor and stratified by the actual value of the pooled stratification factors at randomization (eg, region [Asia vs US/EU], etiology, macrovascular invasion and/or extrahepatic spread [present vs absent] and ECOG). Noninferiority will be declared if the upper limit of the 95% CI for $HR_{A/B}$ is < 1.08.

In the final analysis, the superiority of tislelizumab over sorafenib will be tested for OS, using a stratified log-rank test conducted in the ITT analysis set, but only when noninferiority is demonstrated. Superiority will be declared if the one-sided p-value crosses the boundary specified in the table below in favor of Arm A in the stratified log-rank test at the final analysis. The median OS and cumulative probability of OS estimated at 6-month intervals will be calculated using Kaplan-Meier estimates for each treatment arm, and presented with 2-sided 95% CIs.

Timing and Stopping Boundary in the Interim and Final Analyses of Overall Survival

There will be 1 interim analysis of OS: a test of OS superiority that is planned to occur after 80% of the targeted death events have been observed. The early stopping rule for the interim analysis is set for futility and efficacy.

Specifically, the interim analysis will be performed when approximately 403 deaths (80% of the expected number, approximately 504 deaths) have been reported between the 2 treatment arms. It is estimated that it will take approximately 33.9 months to observe 403 deaths.

The final analysis of OS will take place after approximately 504 deaths have been observed. The upper (efficacy) boundary is based on the O'Brien-Fleming boundary, approximated by the Hwang-Shih-DeCani spending function.

Stopping boundaries (p-value and Z score) of the superiority test for OS at the interim and final analyses are as shown in the following table:

	Time (months)	# Deaths	p-value (Z score) for Efficacy	Approximate Hazard Ratio for Efficacy
Interim analysis	33.9	403	< 0.0110 (> 2.29)	< 0.7958
Final analysis	46.6	504	< 0.02 (> 2.01)	< 0.8358

Secondary Efficacy Analyses:

Objective Response Rate

The key secondary endpoint, ORR, as assessed by BIRC per RECIST v1.1, will be compared between tislelizumab and sorafenib via testing of the null hypothesis:

H_0: ORR in Arm A = ORR in Arm B against the alternative:

H_a: ORR in Arm A > ORR in Arm B

The statistical significance of the difference in ORR between the 2 treatment arms in the ITT analysis set will be evaluated with the Cochran-Mantel-Haenszel chi-square test, with the actual value of the stratification factors as strata tested at an alpha level of 0.05 (2-sided). The 2-sided 95% CIs for the odds ratio and the difference in ORR will be calculated, as will the Clopper-Pearson 95% CIs for the ORR within each arm.

Progression-Free Survival

Progression-free survival will be compared between the 2 treatment arms, using the ITT analysis set and a stratified log-rank test with actual value of the stratification factors as strata.

The median PFS and the cumulative probability of PFS at 3-month intervals will be calculated for each treatment arm and presented with 2-sided 95% CIs. Additionally, PFS will be estimated using the Kaplan-Meier method. The PFS censoring rule will follow the United States Food and Drug Administration (FDA) Guidance for Industry Clinical Trial Endpoints for the Approval of Cancer Drugs and Biologics (FDA Guidance for Industry 2007).

Duration of Response and Time to Progression

Both DOR and TTP, as assessed by BIRC per RECIST v1.1, will be analyzed similarly to the approach used for PFS. The DOR will be summarized within responders.

Health-Related Quality of Life

European Organisation for Research and Treatment of Cancer Quality of Life Cancer Questionnaire-Hepatocellular Carcinoma 18 Questions (EORTC QLQ-HCC18) and European Organisation for Research and Treatment of Cancer Quality of Life Questionnaire-Core 30 (EORTC QLQ-C30) postbaseline index scores will be compared between the 2 treatment arms, using a mixed model. European Quality of Life 5-Dimensions (EQ-5D-5L) will be summarized as well.

Investigator Assessments of Secondary Efficacy Endpoints

Investigator assessments of ORR, PFS, DOR, and TTP per RECIST v1.1 will be analyzed similarly to the corresponding assessments made by BIRC. Best overall response (BOR) is defined in this protocol as the best response recorded from randomization until either the data cutoff date or the date the patient begins a new anticancer treatment, whichever occurs first. Patients with no postbaseline response assessment (regardless of reason) will be considered non-responders in the analysis of BOR.

The proportion and its corresponding Clopper-Pearson 95% CI for each of the response categories (complete response [CR], partial response [PR], stable disease [SD], and progressive disease [PD]) will be presented by treatment arm. Both DCR and CBR will be analyzed using an approach similar to that employed in the ORR analysis, using the ITT analysis set.

Exploratory Analyses:

Distribution of PD-L1 expression will be examined in the ITT analysis set. Any potential association between PD-L1 expression and superior tislelizumab treatment effect over sorafenib will be explored. Other potential predictive markers will also be assessed.

Pharmacokinetic Analysis:

The PK samples will be collected in this study as outlined in Appendix 1.

Tislelizumab postdose and trough serum concentration (C_{trough}) data will be tabulated and summarized by visit/cycle at which these concentrations are collected. Descriptive statistics will include means, medians, ranges, and standard deviations, as appropriate.

Additional PK analyses will be conducted as appropriate.

Exposure-response (efficacy or safety endpoints) analysis may be carried out if supported by data.

Immunogenicity Analysis:

Immunogenicity samples will be collected in this study as outlined in Appendix 1.

The immunogenicity results will be summarized using descriptive statistics by the number and percentage of patients who develop detectable ADAs. The incidences of positive ADAs and neutralizing ADAs will be reported for evaluable patients. The effect of immunogenicity on PK, efficacy and safety may be evaluated if data allow.

Safety Analyses:

Study drug exposure (for both tislelizumab and sorafenib) will be summarized by duration, dosage, and dose intensity for each treatment arm.

Verbatim AE terms will be mapped to the corresponding Medical Dictionary for Regulatory Activities (MedDRA) Preferred Terms and graded per NCI-CTCAE v4.03. All treatment-emergent adverse events (TEAEs) will be summarized. A TEAE is defined in this protocol as any AE or SAE with either an onset date or a date of worsening in severity from baseline (ie, pretreatment) occurring on or after first dose of study drug and up to either 30 days following discontinuation from study drug or start of new anticancer therapy, whichever occurs first. The TEAE classification also applies to irAEs and related SAEs that are recorded up to 90 days after discontinuation from study drug, regardless of whether or not the patient starts a new anticancer therapy.

All SAEs, deaths, TEAEs of ≥ Grade 3 severity, TEAEs assessed by the investigator as treatment-related, irAEs, and TEAEs leading to treatment discontinuation, dose reduction, or dose interruption will be summarized. In all AE summary tables, multiple occurrences of the same AE will be counted once at the maximum severity grade within a particular MedDRA System Organ Class and Preferred Term.

All clinical laboratory and vital sign assessments yielding values outside the normal ranges will be identified. Select laboratory and vital sign data will be summarized by NCI-CTCAE v4.03 severity grade.

Sample Size:

The sample size calculation is based on the number of events required to demonstrate the noninferiority and superiority of tislelizumab over sorafenib (ie, Arm A over Arm B) in the comparison of OS.

Under the original OS HR assumption of 0.75 (13.3 months in Arm A versus 10.0 months in Arm B), approximately 504 deaths in total were planned to have approximately 89% power in the superiority test and 98.4% power in the noninferiority test with a 1.08 noninferiority margin. A total of 640 patients were to be randomized in a 1:1 ratio to Arms A and B over a 16-month period at a constant enrollment rate (40 patients/month). An interim analysis was planned after 75% of the targeted number of OS events (ie, approximately 378 deaths) occurred.

After reviewing recent data, the interim analysis was postponed until approximately 80% of the targeted number of OS events (ie, 403 deaths) were observed, with the planned number of deaths in the final analysis remaining at approximately 504. At the time of protocol amendment 5.0, enrollment was completed with a total of 674 patients randomized. Using a more conservative HR assumption of 0.79 at the time of final analysis after an initial 7-month delayed treatment effect (ie, assuming HR = 1 in the first 7 months), the estimated powers for the superiority test are 44% in the interim analysis and 72% in the final analysis using a simulation. The targeted number of events are estimated to occur approximately 33.9 and 46.6 months after study initiation under the actual enrollment rates, updated HR assumptions, and a median OS of 13.5 months in Arm B. The power in the noninferiority test is 0.935 under the updated assumptions.

In the comparison of key secondary endpoint of ORR between the 2 treatment arms, the power of a Miettinen and Nurminen test (Miettinen and Nurminen 1985) comparing 2 binomial rates for superiority in 674 patients is approximately 99%, assuming an ORR of 0.15 and 0.05 in Arms A and B, respectively.

TABLE OF CONTENTS

FINAL PROTOCOL APPROVAL SHEET 3
INVESTIGATOR SIGNATURE PAGE 4
SYNOPSIS 5
TABLE OF CONTENTS 12
LIST OF TABLES 19
LIST OF FIGURES 20
LIST OF ABBREVIATIONS AND DEFINITIONS OF TERMS 21
1. INTRODUCTION 26
1.1. Hepatocellular Carcinoma 26
1.1.1. Treatment Options for Hepatocellular Carcinoma 26
1.2. Tislelizumab 29
1.2.1. Clinical Pharmacology 29
1.2.2. Summary of Relevant Clinical Experience with Tislelizumab 30
1.2.3. Benefit-Risk Assessment 35
1.3. Study Conduct 36
2. STUDY OBJECTIVES AND ENDPOINTS 37
2.1. Objectives 37
2.1.1. Primary Objectives 37
2.1.2. Secondary Objectives 37
2.1.3. Exploratory Objectives 37
2.2. Endpoints 37
2.2.2. Secondary Endpoints 38
2.2.3. Exploratory Endpoints 38
3. STUDY DESIGN 39
3.1. Summary of Study Design 39
3.2. Study Schematic 39
3.3. Duration of Study 40
3.3.1. Screening Phase 40
3.3.2. Treatment Phase 40
3.3.3. Safety Follow-up Phase 41
3.3.4. Survival Follow-up Phase 41

3.4. Study Rationales 41
3.4.1. Rationale for Tislelizumab in the Treatment of Hepatocellular Carcinoma.....41
3.4.2. Rationale for Selection of Tislelizumab Dose 41
3.4.3. Rationale for Selection of Sorafenib as Comparator 42
4. MATERIALS AND METHODS 43
4.1. Selection of Study Population 43
4.1.1. Inclusion Criteria 43
4.1.2. Exclusion Criteria 44
5. STUDY TREATMENT 47
5.1. Formulation, Packaging, Handling, and Storage 47
5.1.1. Tislelizumab 47
5.1.2. Sorafenib 47
5.2. Dosage, Administration, and Compliance 48
5.2.1. Tislelizumab 48
5.2.2. Sorafenib 49
5.3. Handling of Overdose 49
5.4. Investigational Medicinal Product Accountability 49
5.5. Dose Modifications or Delays 50
5.5.1. Dose Modification for Tislelizumab 50
5.5.2. Dose Modification for Sorafenib 50
5.6. Disposal and Destruction 51
6. PRIOR AND CONCOMITANT THERAPY 52
6.1. Prior Therapy 52
6.2. Concomitant Therapy 52
6.2.1. Permitted Therapy 52
6.2.2. Excluded (Prohibited or Restricted) Therapy 53
7. STUDY ASSESSMENTS AND PROCEDURES 55
7.1. Screening 55
7.1.1. Demographic and Medical History 55
7.1.2. Females of Childbearing Potential and Contraception 56
7.1.3. Informed Consent Form and Screening Log Completion 56
7.1.4. Pulmonary Function Tests 56
7.2. Enrollment 56

7.2.1. Confirmation of Eligibility 56
7.2.2. Patient Numbering 57
7.2.3. Randomization 57
7.2.4. Tislelizumab and Sorafenib Dispensation 57
7.3. Safety Assessments 57
7.3.1. Vital Signs 57
7.3.2. Physical Examinations 57
7.3.3. Eastern Cooperative Oncology Group Performance Status Grading 58
7.3.4. Laboratory Safety Tests 58
7.3.5. Electrocardiograms 59
7.3.6. Adverse Events 59
7.3.7. Ophthalmologic Examination 59
7.4. Tumor and Response Evaluations 59
7.5. Pharmacokinetic and Antidrug Antibody Testing 61
7.6. Biomarkers 61
7.7. Health-Related Quality of Life Assessment 62
7.8. Visit Windows 62
7.9. Unscheduled Visits 62
7.10. End of Treatment Visit 62
7.11. Safety Follow-up Visit 62
7.12. Survival Follow-up 63
7.13. Patient, Treatment, Study, and Site Discontinuation 63
7.13.1. Discontinuation of Study Treatment 63
7.13.2. Study Termination and Study Site Closure 65
8. SAFETY MONITORING AND REPORTING 66
8.1. Risks Associated with Tislelizumab 66
8.2. Risks Associated with Sorafenib 66
8.3. General Plan to Manage Safety Concerns 66
8.3.1. Eligibility Criteria 66
8.3.2. Safety Monitoring Plan 67
8.4. Adverse Events 67
8.4.1. Definition of Adverse Events 67
8.4.2. Assessment of Severity 68

8.4.3. Assessment of Causality 68
8.4.4. Following Adverse Events 69
8.4.5. Laboratory Test Abnormalities 70
8.5. Definition of a Serious Adverse Event 70
8.6. Suspected Unexpected Serious Adverse Reaction 71
8.7. Timing, Frequency, and Method of Capturing Adverse Events 71
8.7.1. Adverse Event Reporting Period 71
8.7.2. Reporting Serious Adverse Events 71
8.7.3. Eliciting Adverse Event Information 72
8.7.4. Recording Diagnosis Versus Signs and Symptoms 72
8.7.5. Recording Adverse Events Occurring Secondary to Other Events 73
8.7.6. Recording Persistent or Recurring Adverse Events 73
8.7.7. Recording Disease Progression 73
8.7.8. Recording Deaths 74
8.7.9. Recording Pregnancies 74
8.7.10. Recording Post-Study Adverse Events 74
8.7.11. Expedited Reporting to Health Authorities, Investigators, Institutional Review Boards, and Independent Ethics Committees 74
8.7.12. Assessing and Recording Immune-Related Adverse Events 74
8.8. Management of Adverse Events of Special Interest 75
8.8.1. Infusion-Related Reactions 75
8.8.2. Immune-Related Adverse Events 77
8.8.3. Hepatic and Renal Function Abnormalities 78
9. STATISTICAL METHODS AND ANALYSIS PLAN 80
9.1. Analysis Sets 80
9.2. Patient Disposition 80
9.3. Demographic and Other Baseline Characteristics 80
9.4. Prior and Concomitant Medications 80
9.5. Efficacy Analyses 81
9.5.1. Primary Efficacy Analyses 81
9.5.2. Secondary Efficacy Analyses 82
9.6. Safety Analyses 84
9.6.1. Extent of Exposure 84

9.6.2. Adverse Events 84
9.6.3. Laboratory Analyses 85
9.6.4. Vital Signs Analyses 85
9.6.5. Ophthalmologic Examination 85
9.7. Pharmacokinetic Analyses 85
9.8. Immunogenicity Analyses 85
9.9. Other Exploratory Analyses 86
9.10. Determination of Sample Size 86
9.11. Interim Analyses 86
10. STUDY COMMITTEES AND COMMUNICATION 88
10.1. Blinded Independent Review Committee 88
10.2. Independent Data Monitoring Committee 88
11. SOURCE DOCUMENTS AND ACCESS TO SOURCE DATA/DOCUMENTS 89
11.1. Access to Information for Monitoring 89
11.2. Access to Information for Auditing or Inspections 89
12. QUALITY ASSURANCE AND QUALITY CONTROL 90
12.1. Regulatory Authority Approval 90
12.2. Quality Assurance 90
12.3. Study Site Inspections 90
12.4. Drug Accountability 90
13. ETHICS/PROTECTION OF HUMAN SUBJECTS 92
13.1. Ethical Standard 92
13.2. Institutional Review Board/Independent Ethics Committee 92
13.2.1. Protocol Amendments 92
13.3. Informed Consent 93
13.4. Patient and Data Confidentiality 93
14. FINANCIAL DISCLOSURE 95
15. DATA HANDLING AND RECORD KEEPING 96
15.1. Data Collection and Management Responsibilities 96
15.1.1. Data Collection 96
15.1.2. Data Management/Coding 96
15.2. Data Integrity 97

15.3. Study Records Retention 97
16. PROTOCOL DEVIATIONS 99
17. PUBLICATION AND DATA SHARING POLICY 100
17.1. Study and Study Center Closure 100
17.2. Information Disclosure and Inventions 101
18. REFERENCES 103
19. APPENDICES 107
APPENDIX 1. SCHEDULE OF ASSESSMENTS 108
APPENDIX 2. CHILD-PUGH CLASSIFICATION SCORING SYSTEM 113
APPENDIX 3. THE RESPONSE EVALUATION CRITERIA IN SOLID TUMORS (RECIST) GUIDELINES, VERSION 1.1 114
APPENDIX 4. PRE-EXISTING IMMUNE DEFICIENCIES OR AUTOIMMUNE DISEASES 123
APPENDIX 5. CLINICAL LABORATORY ASSESSMENTS 124
APPENDIX 6. CONTRACEPTION GUIDELINES AND DEFINITIONS OF “WOMEN OF CHILDBEARING POTENTIAL”, “NO CHILDBEARING POTENTIAL” 125
APPENDIX 7. NEW YORK HEART ASSOCIATION FUNCTIONAL CLASSIFICATION 127
APPENDIX 8. CHRONIC KIDNEY DISEASE EPIDEMIOLOGY COLLABORATION (CKD-EPI) EQUATION 128
APPENDIX 9. DOSE MODIFICATION GUIDELINE FOR SORAFENIB 129
APPENDIX 10. IMMUNE-RELATED ADVERSE EVENT EVALUATION AND MANAGEMENT 131
APPENDIX 11. LIST OF CHINESE HERBAL MEDICINE OR CHINESE PATENT MEDICINES WHICH HAVE EFFECT OF CONTROL CANCER OR BOOST IMMUNITY 141
APPENDIX 12. LIST OF COUNTRY SPECIFIC PROTOCOL AMENDMENTS 143
APPENDIX 13. SAFETY RUN-IN SUBSTUDY INVESTIGATING SAFETY, TOLERABILITY, PHARMACOKINETICS AND PRELIMINARY ANTITUMOR ACTIVITY OF ANTI-PD-1 MONOCLONAL ANTIBODY TISLELIZUMAB IN JAPANESE PATIENTS WITH UNRESECTABLE HEPATOCELLULAR CARCINOMA 144
SYNOPSIS FOR SUBSTUDY 145
1. INTRODUCTION 149
2. STUDY OBJECTIVES AND ENDPOINTS 149

2.1. Objectives 149
2.1.1. Primary Objectives 149
2.1.2. Secondary Objectives: 149
2.1.3. Exploratory Objectives 149
2.2. Endpoints 150
2.2.1. Primary Endpoints 150
2.2.2. Secondary Endpoints 150
2.2.3. Exploratory Endpoints 150
3. STUDY DESIGN 150
3.1. Summary of Study Design 150
3.2. Schedule of Study Assessments 151
3.3. Duration of Study 158
3.4. Dose-Limiting Toxicities 158
3.5. Study Rationale 158
4. MATERIALS AND METHODS 159
4.1. Selection of Study Population 159
4.2. Inclusion Criteria 159
4.3. Exclusion Criteria 160
5. STUDY TREATMENT 162
5.1. Formulation, Packaging, Handling, and Storage 162
5.2. Dosage, Administration, and Compliance 162
5.3. Handling of Overdose 163
5.4. Investigational Medicinal Product Accountability 163
5.5. Disposal and Destruction 163
6. PRIOR AND CONCOMITANT THERAPY 163
7. STUDY ASSESSMENTS AND PROCEDURES 163
8. SAFETY MONITORING AND REPORTING 164
9. STATISTICAL METHODS AND ANALYSIS PLAN 165
9.1. Analysis Sets 165
9.2. Patient Disposition 165
9.3. Demographic and Other Baseline Characteristics 165
9.4. Prior and Concomitant Therapies 166
9.5. Analyses 166

9.5.1. Primary Analyses 166
9.5.2. Secondary Analyses 166
9.5.3. Exploratory Analyses 166
9.6. Safety Analyses 166
9.7. Pharmacokinetic Analyses 167
9.8. Immunogenicity Analyses 167
10. STUDY COMMITTEES AND COMMUNICATION 167
11. SOURCE DOCUMENTS AND ACCESS TO SOURCE DATA/DOCUMENTS 167
12. QUALITY ASSURANCE AND QUALITY CONTROL 167
13. ETHICS/PROTECTION OF HUMAN SUBJECTS 168
14. DATA HANDLING AND RECORD KEEPING 168
APPENDIX 14. REGIONAL PRODUCT LABELS FOR SORAFENIB 169

LIST OF TABLES

Table 1: Treatment-Emergent Adverse Events, Assessed as Related to Tislelizumab, Occurring in ≥ 5% of Patients and Corresponding Incidence at ≥ Grade 3 Severity – Study BGB-A317_001, Phase 1a 31
Table 2: Treatment-Emergent Adverse Events Considered Immune-Related in Study BGB-A317_001 – Pooled Phases 1a and 1b 32
Table 3: Selection and Timing of Dose for Each Patient 48
Table 4: Eastern Cooperative Oncology Group Performance Status – Grading System 58
Table 5: Timeframes and Documentation Methods for Reporting Serious Adverse Events to the Sponsor or Designee 71
Table 6: Treatment Modification Guidelines for Symptoms of Infusion-Related Reactions Due to Tislelizumab 75
Table 7: Immune-Related Adverse Events Associated with Anti-PD-1 Drugs 77
Table 8: Stopping Boundaries (p-value and Z score) and Approximate Hazard Ratio Threshold for Interim and Final Analyses of Superiority Test for Overall Survival 82
Appendix 13 Table 1: Japan Substudy Schedule of Assessments 152
Appendix 13 Table 2: Schedule of Japan Substudy Pharmacokinetic and Anti-Tislelizumab Antibody Sampling 157

LIST OF FIGURES

Figure 1: Study Design Schematic 39

LIST OF ABBREVIATIONS AND DEFINITIONS OF TERMS

Abbreviation	Definition
AASLD	American Association for the Study of Liver Disease
ADA	Antidrug antibody
ADCC	Antibody-dependent cellular cytotoxicity
ADCP	Antibody-dependent cellular phagocytosis
ADL	Activities of daily living
AE	Adverse event
AFP	Alpha fetoprotein
ALT	Alanine aminotransferase
ASCO	American Society of Clinical Oncology
AST	Aspartate aminotransferase
ATC	Anatomical Therapeutic Category
AUC	Area under the concentration-time curve
$AUC_{0\text{-}14d}$	Area under the concentration-time curve from Day 0 to Day 14
$AUC_{0\text{-}21d}$	Area under the concentration-time curve from Day 0 to Day 21
BCLC	Barcelona Clinic Liver Cancer
BGB-A317	Code name for monoclonal antibody tislelizumab
BID	Twice daily
BIRC	Blinded Independent Review Committee
BOR	Best overall response
CBR	Clinical benefit rate
CI	Confidence interval
CK	Creatine kinase
CK-MB	Creatine kinase cardiac muscle isoenzyme
CL	Clearance
C_{max}	Maximum observed plasma concentration
CP	Child-Pugh
CP-A	Child-Pugh Class A
CP-B	Child-Pugh Class B
CR	Complete response
CSR	Clinical Study Report
CT	Computed tomography
C_{trough}	Trough serum concentration
CTCAE	Common Terminology Criteria for Adverse Events

Abbreviation	Definition
CYP 3A4	Cytochrome P450 3A4 isoenzyme
DCR	Disease control rate
DLT	Dose-limiting toxicity
DNA	Deoxyribonucleic acid
DOR	Duration of response
eCCGs	Electronic Case Report Form Completion Guidelines
ECG	Electrocardiogram
ECOG	Eastern Cooperative Oncology Group
eCRF	Electronic case report form
EDC	Electronic data capture
eGFR	Estimated glomerular filtration rate
EORTC QLQ-C30	European Organisation for Research and Treatment of Cancer Quality of Life Questionnaire-Core 30
EORTC QLQ-HCC18	European Organisation for Research and Treatment of Cancer Quality of Life Questionnaire-Hepatocellular Carcinoma 18 Questions
EQ-5D-5L	5-level version of European Quality of Life 5-Dimensional Questionnaire
ESMO	European Society for Medical Oncology
EU	European Union
FcγR	Gamma Fc receptor (eg, Fcγ-RI, Fcγ-R2I)
FDA	Food and Drug Administration
FDG-PET	Fluorodeoxyglucose-position emission tomography
FFPE	Formalin-fixed paraffin-embedded
FSH	Follicle Stimulating Hormone
GCP	Good Clinical Practice
GI	Gastrointestinal
HBcAb	Hepatitis B core antibody
HBsAg	Hepatitis B surface antigen
HBV	Hepatitis B virus
HCC	Hepatocellular carcinoma
HCV	Hepatitis C virus
HR	Hazard ratio
HRQoL	Health-related quality of life
IB	Investigator’s Brochure
ICF	Informed consent form
ICH	International Conference on Harmonisation

Abbreviation	Definition
ICU	Intensive Care Unit
IDMC	Independent Data Monitoring Committee
IDMS	Isotope dilution mass spectrometry
IEC	Independent Ethics Committee
IFN-α	Interferon-alpha
IFN-γ	Interferon gamma
Ig	Immunoglobulin
IgA	Immunoglobulin A
IgG	Immunoglobulin G (eg, IgG1, IgG2, IgG3, IgG4)
IMP	Investigational medicinal product
IND	Investigational New Drug
INR	International normalized ratio
irAE	Immune-related adverse event
IRB	Institutional Review Board
IRT	Interactive response technology
ITT	Intent-to-Treat
IUD	Intrauterine device
IUS	Intrauterine hormone-releasing system
IV	Intravenous(ly)
K_D	Dissociation constant
MCH	Mean corpuscular hemoglobin
MDSC	Myeloid-derived suppressor cells
MDRD	Modification of Diet in Renal Disease
MedDRA	Medical Dictionary for Regulatory Activities
MRI	Magnetic resonance imaging
MSI	Microsatellite instability
MTD	Maximum tolerated dose
^{18}F-NaF PET	18F-sodium fluoride position emission tomography
NCI-CTCAE	National Cancer Institute Common Terminology Criteria for Adverse Events
NE	Not evaluable
NCCN	National Comprehensive Cancer Network
NKDEP	National Kidney Disease Education Program
NSAID	Nonsteroidal anti-inflammatory drug
ORR	Objective response rate

Abbreviation	Definition
OS	Overall survival
PBMC	Peripheral blood mononuclear cell
PD	Progressive disease
PD-1	Programmed cell death protein-1
PD-L1	Programmed cell death ligand-1
PD-L2	Programmed cell death ligand-2
PET	Positron emission tomography
PFS	Progression-free survival
PI	Prescribing Information
PK	Pharmacokinetic(s)
PO	Orally
PP	Per-Protocol
PR	Partial response
PS	Performance Status
PT	Prothrombin time
Q2W	Once every 2 weeks
Q3W	Once every 3 weeks
QTc	QT interval corrected for heart rate
RBC	Red blood cell
RECIST	Response Evaluation Criteria in Solid Tumors
RNA	ribonucleic acid
RSI	Reference Safety Information
SAE	Serious adverse event
SAP	Statistical Analysis Plan
SD	Stable disease
SOC	System Organ Class
SpO_2	Peripheral oxygen saturation
SUSAR	Suspected unexpected serious adverse reaction
TACE	Transarterial chemoembolization
TC-99m	Technetium-99m
TEAE	Treatment-emergent adverse event
T_{max}	Time to maximum plasma concentration
$t_{1/2}$	Elimination half-life
TSH	Thyroid stimulating hormone

Abbreviation	Definition
TTP	Time to progression
ULN	Upper limit of normal
US, USA	United States
USP	United States Pharmacopeia
Vd	Volume of distribution
WHO	World Health Organization

1. INTRODUCTION

1.1. Hepatocellular Carcinoma

Hepatocellular carcinoma (HCC) is a major global health problem, accounting for 85-90% of all reported cases of liver cancer (a term with which HCC is often used interchangeably) (El-Serag 2007). According to the World Health Organization's GLOBOCAN 2012 database, liver cancer was the sixth most common type of cancer that year, with 782,000 new cases worldwide; it was also the second most common cause of cancer-related mortality, responsible for an estimated 746,000 deaths (Torre 2015).

Most HCC cases (> 80%) occur in Eastern Asia and in sub-Saharan Africa, with typical incidence rates of > 20 per 100,000 individuals. China alone accounts for approximately 50% of both new HCC cases and HCC-related deaths worldwide (Torre 2015). Southern European countries, such as Spain, Italy, and Greece, tend to have more moderate incidence rates (~10 to 20 per 100,000 individuals), whereas North America, South America, Northern Europe, and Oceania have a relatively low incidence of HCC (< 5 per 100,000 individuals) (El-Serag 2012).

A variety of risk factors are known to be causative for HCC. These include infection with hepatitis viruses, aflatoxin B, tobacco, vinyl chloride, heavy alcohol intake, non-alcoholic fatty liver disease, hemochromatosis, and diabetes. Together, hepatitis B virus (HBV) and hepatitis C virus (HCV) account for 80-90% of all HCC cases worldwide (Bosch 2005). Chronic HBV infection is the dominant risk factor for the disease in most areas of Asia, with the exception of Japan (El-Serag 2012), while chronic infection with HCV is the leading cause of HCC in Western countries and in Japan (Choo 2016).

Treatment options for HCC are based on the stage of the disease at diagnosis per the Barcelona Clinic Liver Cancer (BCLC) classification system, which draws from a combination of Eastern Cooperative Oncology Group (ECOG) Performance Status (Table 4), Child-Pugh classification criteria for liver function (Appendix 2), and extent of disease (Llovet 2004) to define disease staging for HCC. Approximately 30% of HCC cases are diagnosed at the early stages (ie, BCLC stages 0 or A) and are amenable to potentially curative treatments, including liver transplantation, resection, or loco-regional procedures such as radiofrequency ablation and percutaneous ethanol injection. However, there is a high rate (70%) of HCC recurrence within 5 years (Oikonomopoulos 2016).

In intermediate-stage HCC (ie, BCLC Stage B), transarterial chemoembolization (TACE) is the recommended treatment modality (Han 2015) for these patients, but the data are difficult to interpret in the context of BCLC staging; the data supporting the recommendation were not categorized according to BCLC classification (Han 2015). Nonetheless, patients do progress after TACE treatment and become ineligible for further TACE therapy.

For patients with advanced disease (ie, BCLC Stage C), sorafenib, a multitargeted tyrosine kinase inhibitor, is the only globally approved systemic therapy for HCC (Keating 2017). Details of sorafenib treatment in the setting of advanced HCC are provided below.

1.1.1. Treatment Options for Hepatocellular Carcinoma

The majority (approximately 70%) of patients diagnosed with HCC present with unresectable disease (Mazzaferro 1996). In 2008, sorafenib (NEXAVAR Prescribing Information [PI]), a

multi-kinase inhibitor, was approved by the United States (US) Food and Drug Administration (FDA) for use in this patient population. It inhibits multiple intracellular (CRAF, BRAF) and cell surface kinases (KIT, FLT-3, RET, VEGFR1/2/3, and PDGFRβ), which impede tumor growth and angiogenesis.

The clinical efficacy of sorafenib in the HCC patient population has been well-documented in 2 key studies (Balogh 2016): the "SHARP" trial and the Asia-Pacific trial, as described below.

The SHARP study was a multicenter, double-blinded, placebo-controlled trial conducted in Europe, North America, South America, and Australasia. The study, which enrolled 602 patients with advanced HCC, well-preserved liver function, and no history of prior systemic therapy, found that sorafenib significantly improved both overall survival (OS) and time to radiologic progression as compared to placebo: median OS was 10.7 months in the sorafenib group compared to 7.9 months in the placebo group (hazard ratio [HR]: 0.69; 95% confidence interval [CI]: 0.55-0.87; $p < 0.001$) (Llovet 2008), while median time to radiologic progression was 5.5 months in sorafenib-treated patients compared to 2.8 months in placebo-treated patients ($p < 0.001$) and overall response rate assessed by investigator was 2% in sorafenib-treated patients compared to 1% in placebo-treated patients ($p = 0.05$).

Whereas the SHARP study primarily included patients from Europe and the Americas, a second Phase 3 trial with a comparable design enrolled 271 Asian-Pacific patients (from China, Taiwan, and South Korea) with the same clinical profile as patients in the SHARP study. This trial, too, established a significant treatment benefit for sorafenib compared to placebo (median OS: 6.5 vs 4.2 months; HR: 0.68; 95% CI: 0.50-0.93; $p = 0.014$) (Cheng 2009).

Sorafenib is also an appropriate treatment option for patients with intermediate-stage HCC (BCLC Stage B) whose disease has progressed after loco-regional therapy (typically, chemoembolization). Retrospective data from the SHARP study showed an HR of 0.72 for OS in this particular group of patients, comparable to the HR generated for the overall study population OS (Finn 2015).

Furthermore, sorafenib is indicated for treatment of HCC patients with Child-Pugh Class A (CP-A) and Child-Pugh Class B (CP-B) liver function. Use of sorafenib in CP-B patients was evaluated in a Phase 2 study of 137 patients with advanced HCC, 28% of whom were CP-B. Results showed sorafenib to be generally well tolerated and to demonstrate similar antitumor activity between the 2 groups. There were no significant differences in pharmacokinetics (PK) between CP-A and CP-B patients (Abou-Alfa 2006).

To further evaluate the efficacy and safety of sorafenib in CP-B patients, the GIDEON registry study was conducted and included > 3000 patients globally. Overall incidence of adverse events (AEs) was observed to be comparable between CP-A and CP-B patients, as was the incidence of Grade 3 or 4 AEs. Though serious adverse events (SAEs) were more common in CP-B patients (60% vs 36%), the higher incidence was noted in patients whose CP scores were ≥ 8 (Marrero 2016).

Taken altogether, these clinical data have illustrated a robust sorafenib-derived treatment benefit for HCC patients, irrespective of the underlying cause of their disease (Lee 2010). Currently, sorafenib is recommended in all published guidelines (Keating 2017, Heimbach 2017, National Comprehensive Cancer Network [NCCN] 2017) for patients with advanced HCC and relatively

preserved liver function who are not candidates for either resection or liver transplantation, and who have progressed after loco-regional therapies (Bruix 2012).

However, sorafenib is difficult for patients to tolerate. The most common side effects include hypertension, hemorrhage, hand-foot skin reaction, diarrhea, sensory neuropathy, weight loss, rash, alopecia, anorexia, and pain in abdomen (NEXAVAR PI). As reported in an observational field study, 54% and 40% of patients treated with sorafenib have required dose reduction and treatment interruption, respectively (Iavarone 2011). Given that the median OS in advanced-stage HCC patients treated with sorafenib is < 12 months, and in light of the relatively poor tolerability of the drug, additional novel therapy is clearly needed in this patient population.

Another investigational tyrosine kinase inhibitor, lenvatinib, has been compared to sorafenib in terms of its efficacy in treating advanced-stage HCC. The Phase 3 study, Study 304, conducted in patients with unresectable HCC, has established the noninferiority of lenvatinib to sorafenib in a comparison of OS (Eisai 2017). However, lenvatinib has not been approved in any country for the treatment of HCC and thus, cannot be considered part of global standard of care.

At present, sorafenib remains the only systemic agent approved worldwide for the treatment of advanced HCC in the first-line setting.

1.1.1.1. Anti-PD-1/Anti-PD-L1 Therapy for Advanced Hepatocellular Carcinoma

The immune checkpoint-inhibitory receptor known as programmed cell death protein-1 (PD-1) is mainly expressed in activated T-cells (Topalian 2012, Bersanelli 2017). The PD-1 signaling cascade negatively regulates T-cell receptor activities while attenuating T-cell proliferation and function, with the ultimate consequence of T-cell exhaustion.

The expression of PD-1 is markedly upregulated in tumor-infiltrating lymphocytes, and the expression of programmed cell death ligand-1 (PD-L1) is significantly increased in tumor cells and tumor-associated immune cells in the presence of stimulating cytokines (eg, interferon-alpha [IFN-α] and interferon gamma [IFN-γ]) in the tumor microenvironment. Furthermore, increased PD-1 expression in tumor-infiltrating lymphocytes and/or PD-L1 expression in tumor cells and tumor-associated stromal cells have been observed in many types of solid tumors (Jin 2016, Ono 2017, Patel 2015, Van Der Kraak 2016, McDaniel 2016, Gong 2011).

These data provide a basis for the use of PD-1 antagonists as immuno-oncologic agents. The therapeutic approach of blocking PD-1 and PD-L1 interactions has recently demonstrated efficacy in a variety of tumor types. Monoclonal antibodies to PD-1, such as nivolumab and pembrolizumab, have the ability to bind to PD-1, thus disrupting interactions between the protein and its ligands (PD-L1 and programmed cell death ligand-2 [PD-L2]) and impeding inhibitory signals in the T-cell microenvironment (Wang 2014). These monoclonal antibodies have now been approved for the treatment of several cancers, including bladder, lung, head and neck squamous cell carcinomas, as well as melanoma, in the US, Europe, and beyond.

Recently published results from an ongoing, open-label, Phase 1b/2 study of nivolumab in patients with advanced HCC ("CheckMate040") (El-Khoueiry 2017) include an objective response rate (ORR) of 20% (95% CI: 15-26) in 214 patients treated in the study's dose expansion phase. The median duration of response (DOR) for all patients in the dose-escalation phase was 9.9 months and the 9-month OS rate was 74% (95% CI: 67-79%). These results are

very promising and suggest the potential efficacy of anti-PD-1 antibodies as therapies for patients with advanced-stage, or otherwise difficult-to-treat, HCC.

1.2. Tislelizumab

Tislelizumab is a humanized, immunoglobulin (Ig)G4-variant monoclonal antibody against PD-1 under clinical development for the treatment of several human malignancies.

Tislelizumab acts by binding to the extracellular domain of human PD-1 with high specificity as well as high affinity (dissociation constant [K_D] = 0.15 nM). It competitively blocks binding efforts by both PD-L1 and PD-L2; thus, inhibiting PD-1-mediated negative signaling in T-cells. In *in vitro* cell-based assays, tislelizumab was observed to consistently and dose-dependently enhance the functional activity of human T-cells and pre-activated, primary peripheral blood mononuclear cells (PBMCs). In addition, tislelizumab has demonstrated antitumor activity in several allogeneic xenograft models, in which PBMCs were co-injected with human cancer cells (A431 [epidermoid carcinoma]) or tumor fragments (BCCO-028 [colon cancer]) into immunocompromised mice.

The immunoglobulin G (IgG)4 variant antibody has very low binding affinity to FcγRIIIA and complement 1q by in vitro assays, suggesting either low or no antibody-dependent cellular cytotoxicity (ADCC) or complement-dependent cytotoxicity effects in humans (Labrijn 2009).

Please refer to the tislelizumab Investigator's Brochure for additional details regarding nonclinical studies.

1.2.1. Clinical Pharmacology

1.2.1.1. Pharmacokinetics

An interim PK analysis (data cutoff date of 08 October 2016) was conducted by noncompartmental analysis methods, using serum concentrations from patients who received doses of tislelizumab 0.5, 2.0, 5.0, and 10 mg/kg once every 2 weeks (Q2W) and patients who received doses of 2.0 and 5.0 mg/kg once every 3 weeks (Q3W) in Study BGB-A317_001 (Phase 1a, Part 1 and Part 2). The maximum observed plasma concentration (C_{max}) and drug exposure (ie, the area under the concentration-time curve [AUC]) increased in a nearly dose proportional manner from 0.5 mg/kg to 10 mg/kg, both after single-dose administration and at steady state.

Population PK analysis was conducted with a 2-compartment model with first order elimination. Systemic clearance of BGB A317 was 0.00794 L/h, volume of distribution in the central and peripheral compartment were 2.75 and 1.65 L, respectively, and terminal elimination half-life was approximately 17 days.

Patients' body weight is not a significant covariate on the clearance of tislelizumab, which supports fixed dosing.

1.2.1.1.1. Lack of Ethnic Differences in Exposure

Based on the information available to date, tislelizumab exposure in Asian and Caucasian patients is similar, and the safety profile at clinically relevant doses is tolerable and manageable.

Preliminary PK data from Study BGB-A317_001 are summarized in Section 1.2.1.1 above. Comparison of PK parameters indicates that after a single intravenous (IV) infusion of tislelizumab, dose-normalized exposure was consistent across Asian (n = 10) and Caucasian (n = 93) patients in the study, which was conducted in the US, Australia, New Zealand, Korea, and Taiwan. Additionally, dose-normalized exposure was consistent between BGB-A317_Study_001, in which most patients were Caucasian (n = 107) and Study BGB-A317-102, conducted in Chinese patients (n = 6).

These preliminary findings indicate that ethnic differences are unlikely to affect the exposure of tislelizumab.

Furthermore, these data are consistent with findings of limited ethnic differences in studies of therapeutic monoclonal antibodies (Chiba 2014). In addition, there do not appear to be clinically relevant differences in PK exposures from studies of 2 other anti-PD-1 antibodies, nivolumab and pembrolizumab (Yamamoto 2017 and Yamazaki 2017).

1.2.2. Summary of Relevant Clinical Experience with Tislelizumab

For details of ongoing studies with tislelizumab, please refer to the most recent tislelizumab Investigator's Brochure, where the most current information is available regarding the clinical experience with tislelizumab.

As of a 13 January 2017 data cutoff, 403 patients have received ≥ 1 dose of tislelizumab in the monotherapy Study BGB-A317_001, the results of which are briefly summarized below.

1.2.2.1. Study BGB-A317_001

Study BGB-A317_001 is a 2-stage trial consisting of a) a Phase 1a, 3-part, dose-escalation and dose-finding component with the key objectives of establishing a maximum tolerated dose (MTD), if any, as well as a recommended Phase 2 dose or doses and b) a subsequent Phase 1b, efficacy-evaluation component examining the clinical activity of tislelizumab in treating select tumor types.

Phase 1a Component (Parts 1-3):

The primary objective of the Phase 1a component of Study BGB-A317_001 is to assess the safety and tolerability of tislelizumab in patients with advanced tumors of various types. This component is divided into 3 parts, as follows:

Part 1: Multiple-dose, dose-escalation, first-in-human study

Part 2: Evaluation of the safety and PK of 2 dosing schedules: Q2W versus Q3W at selected doses

Part 3: Assessment of the safety and PK of tislelizumab administered at fixed doses

In Part 1 of the Phase 1a component, the maximum administered dose of tislelizumab was 10 mg/kg Q2W. The MTD was not identified. Only 1 dose-limiting toxicity (DLT) (Grade 3 colitis) occurred (in a patient receiving tislelizumab at 5 mg/kg Q2W).

Based on the overall safety findings in 103 patients receiving tislelizumab monotherapy in Parts 1 and 2 of Phase 1a, tislelizumab 5 mg/kg Q3W was selected as the dose to be explored in the subsequent Phase 1b component.

Phase 1b Component:

The Phase 1b component is a multicenter, open-label, multiple-arm, indication-expansion study. The primary objective of this component is to assess the antitumor activity of tislelizumab against select tumor types.

1.2.2.1.1. Safety Results (13 January 2017 Data Cutoff)

Phase 1a Component:

As of a data cutoff of 13 January 2017, treatment-emergent adverse events (TEAEs) that were assessed by the investigator as related to tislelizumab had been reported in 72% of patients across all 3 parts of the Phase 1a component. The most frequently occurring treatment-related TEAEs included fatigue (22%), diarrhea (14%), pruritus (13%), rash (13%), and nausea (10%). There was no apparent correlation between dose level (2 mg/kg, 5 mg/kg, or 10 mg/kg) and either the incidence or the severity of treatment-related TEAEs.

Eleven of the 111 total patients (10%) experienced treatment-related TEAEs assessed as related to tislelizumab that were ≥ Grade 3 in severity. Among these events were 2 AEs each of fatigue, diabetic ketoacidosis, and hypotension. Table 1 shows the most frequently occurring treatment-related TEAEs (reported in ≥ 5% of patients [ie, ≥ 6 patients]) in the Phase 1a component of Study BGB-A317_001.

Table 1: Treatment-Emergent Adverse Events, Assessed as Related to Tislelizumab, Occurring in ≥ 5% of Patients and Corresponding Incidence at ≥ Grade 3 Severity – Study BGB-A317_001, Phase 1a

**MedDRA System Organ Class**<br>Preferred Term	**Overall Incidence (All Severity Grades)**	**≥ Grade 3 Severity**
	**N = 111<br>n (%)**	**N = 111<br>n (%)**
**Patients with ≥ 1 treatment-related AE**	**80 (72.1)**	**11 (9.9)**
**Skin and subcutaneous tissue disorders**		
Pruritus	14 (12.6)	0 (0.0)
Rash	14 (12.6)	0 (0.0)
**Gastrointestinal disorders**		
Diarrhea	15 (13.5)	0 (0.0)
Nausea	11 (9.9)	0 (0.0)
**General disorders and administration site conditions**		
Fatigue	24 (21.6)	2 (1.8)
**Endocrine disorders**		
Hypothyroidism	7 (6.3)	0 (0.0)
**Investigations**		
Alanine aminotransferase increased	6 (5.4)	1 (0.9)
**Injury, poisoning and procedural complications**		

MedDRA System Organ Class<br>Preferred Term	Overall Incidence (All Severity Grades)	≥ Grade 3 Severity
	N = 111<br>n (%)	N = 111<br>n (%)
Infusion related reaction	7 (6.3)	0 (0.0)

Notes: Data as of 13 January 2017.
Relationship to study drug is as assessed by investigator.
A TEAE is defined as any AE that starts on or after the dosing date, or (in the case of a continuing AE) worsens in severity during treatment relative to the pretreatment state.
All AEs are coded using MedDRA v17.0 or later and graded according to NCI-CTCAE v4.03.
Any patient with multiple occurrences of the same AE is counted only once in the AE category.
Abbreviations: AE, adverse event; MedDRA, Medical Dictionary for Regulatory Activities; NCI-CTCAE, National Cancer Institute Common Terminology Criteria for Adverse Events; TEAE, treatment-emergent adverse event; v, version.

Phase 1b Component:

As of a 13 January 2017 data cutoff, TEAEs assessed by the investigator as related to tislelizumab had occurred in 41.8% of patients in the Phase 1b component of Study BGB-A317_001. The most frequently reported treatment-related TEAEs included fatigue (9%), rash (5%), nausea (5%), and diarrhea (5%). Ten of the 189 total patients (5%) experienced treatment-related TEAEs that were ≥ Grade 3 in severity, including 2 events each of pneumonitis, diarrhea, and colitis. All other treatment-related TEAEs occurred in a single patient.

Pooled Analysis of Potential Immune-Related Adverse Events of Interest in Study BGB-A317_001:

Table 2 lists all TEAEs (reported as of the 13 January 2017 data cutoff) considered to be potential immune-related adverse events (irAEs) in Study BGB-A317_001.

For this analysis, all TEAEs assessed by the investigator as related to tislelizumab were pooled. The pooled data were then combined with a predetermined "hit-list" of Medical Dictionary for Regulatory Activities (MedDRA) Preferred Terms believed to represent immune-related events and symptoms.

Table 2: Treatment-Emergent Adverse Events Considered Immune-Related in Study BGB-A317_001 – Pooled Phases 1a and 1b

System Organ Class<br>Preferred Term	Worst Grade (N = 300)				Overall Incidence
	Grade 1<br>n (%)	Grade 2<br>n (%)	Grade 3<br>n (%)	Grade 4<br>n (%)	N = 300<br>n (%)
**Patients with ≥ 1 immune-related AE**					**104 (34.7)**
**Skin and subcutaneous tissue disorders**					
Rash	21 (7.0)	3 (1.0)	0 (0.0)	0 (0.0)	24 (8.0)
Pruritus	21 (7.0)	0 (0.0)	0 (0.0)	0 (0.0)	21 (7.0)
Rash maculo-papular	4 (1.3)	3 (1.0)	0 (0.0)	0 (0.0)	7 (2.3)
Pruritus generalized	4 (1.3)	0 (0.0)	0 (0.0)	0 (0.0)	4 (1.3)

System Organ Class<br>Preferred Term	Worst Grade (N = 300)				Overall Incidence
	Grade 1<br>n (%)	Grade 2<br>n (%)	Grade 3<br>n (%)	Grade 4<br>n (%)	N = 300<br>n (%)
Rash erythematous	3 (1.0)	0 (0.0)	0 (0.0)	0 (0.0)	3 (1.0)
Dermatitis	2 (0.7)	0 (0.0)	0 (0.0)	0 (0.0)	2 (0.7)
Acute febrile neutrophilic dermatitis	0 (0.0)	1 (0.3)	0 (0.0)	0 (0.0)	1 (0.3)
Erythema	1 (0.3)	0 (0.0)	0 (0.0)	0 (0.0)	1 (0.3)
Rash macular	1 (0.3)	0 (0.0)	0 (0.0)	0 (0.0)	1 (0.3)
Rash papular	1 (0.3)	0 (0.0)	0 (0.0)	0 (0.0)	1 (0.3)
**Gastrointestinal disorders**					
Diarrhea	16 (5.3)	6 (2.0)	2 (0.7)	0 (0.0)	24 (8.0)
Colitis	0 (0.0)	3 (1.0)	3 (1.0)	0 (0.0)	6 (2.0)
Stomatitis	0 (0.0)	1 (0.3)	1 (0.3)	0 (0.0)	2 (0.7)
Autoimmune pancreatitis	0 (0.0)	0 (0.0)	1 (0.3)	0 (0.0)	1 (0.3)
Rectal hemorrhage	1 (0.3)	0 (0.0)	0 (0.0)	0 (0.0)	1 (0.3)
**Endocrine disorders**					
Hyperthyroidism	6 (2.0)	3 (1.0)	1 (0.3)	0 (0.0)	10 (3.3)
Hypothyroidism	4 (1.3)	6 (2.0)	0 (0.0)	0 (0.0)	10 (3.3)
Diabetes mellitus	0 (0.0)	1 (0.3)	1 (0.3)	0 (0.0)	2 (0.7)
Diabetic ketoacidosis	0 (0.0)	0 (0.0)	1 (0.3)	1 (0.3)	2 (0.7)
Thyroiditis	1 (0.3)	1 (0.3)	0 (0.0)	0 (0.0)	2 (0.7)
Endocrine disorder	1 (0.3)	0 (0.0)	0 (0.0)	0 (0.0)	1 (0.3)
Type 1 diabetes mellitus	0 (0.0)	0 (0.0)	0 (0.0)	1 (0.3)	1 (0.3)
**Investigations**					
Alanine aminotransferase increased	4 (1.3)	2 (0.7)	1 (0.3)	0 (0.0)	7 (2.3)
Aspartate aminotransferase increased	6 (2.0)	1 (0.3)	0 (0.0)	0 (0.0)	7 (2.3)
Blood creatinine increased	2 (0.7)	0 (0.0)	0 (0.0)	0 (0.0)	2 (0.7)
Blood thyroid stimulating hormone decreased	1 (0.3)	0 (0.0)	0 (0.0)	0 (0.0)	1 (0.3)
**Musculoskeletal and connective tissue disorders**					
Arthralgia	7 (2.3)	2 (0.7)	0 (0.0)	0 (0.0)	9 (3.0)
Arthritis	1 (0.3)	1 (0.3)	0 (0.0)	0 (0.0)	2 (0.7)
**Injury, poisoning and procedural complications**					
Infusion related reaction	2 (0.7)	8 (2.7)	0 (0.0)	0 (0.0)	10 (3.3)

System Organ Class<br>Preferred Term	Worst Grade (N = 300)				Overall Incidence
	Grade 1<br>n (%)	Grade 2<br>n (%)	Grade 3<br>n (%)	Grade 4<br>n (%)	N = 300<br>n (%)
**Respiratory, thoracic and mediastinal disorders**					
Dyspnea	3 (1.0)	1 (0.3)	1 (0.3)	0 (0.0)	5 (1.7)
Pneumonitis	0 (0.0)	2 (0.7)	3 (1.0)	0 (0.0)	5 (1.7)
**Hepatobiliary disorders**					
Autoimmune hepatitis	0 (0.0)	1 (0.3)	0 (0.0)	0 (0.0)	1 (0.3)
Hepatic function abnormal	0 (0.0)	1 (0.3)	0 (0.0)	0 (0.0)	1 (0.3)
Hepatitis	0 (0.0)	1 (0.3)	0 (0.0)	0 (0.0)	1 (0.3)
Hyperbilirubinemia	1 (0.3)	0 (0.0)	0 (0.0)	0 (0.0)	1 (0.3)
**Metabolism and nutrition disorders**					
Diabetes mellitus	0 (0.0)	1 (0.3)	1 (0.3)	0 (0.0)	2 (0.7)
Diabetic ketoacidosis	0 (0.0)	0 (0.0)	1 (0.3)	1 (0.3)	2 (0.7)
Hyperglycemia	0 (0.0)	1 (0.3)	1 (0.3)	0 (0.0)	2 (0.7)
Type 1 diabetes mellitus	0 (0.0)	0 (0.0)	0 (0.0)	1 (0.3)	1 (0.3)
**Eye disorders**					
Conjunctivitis	0 (0.0)	1 (0.3)	0 (0.0)	0 (0.0)	1 (0.3)
Uveitis	0 (0.0)	1 (0.3)	0 (0.0)	0 (0.0)	1 (0.3)
**General disorders and administration site conditions**					
Mucosal inflammation	0 (0.0)	0 (0.0)	0 (0.0)	1 (0.3)	1 (0.3)
**Nervous system disorders**					
Peripheral motor neuropathy	1 (0.3)	0 (0.0)	0 (0.0)	0 (0.0)	1 (0.3)
Peripheral sensory neuropathy	1 (0.3)	0 (0.0)	0 (0.0)	0 (0.0)	1 (0.3)

Notes: Data as of 13 January 2017.
Relationship to study drug is as assessed by investigator.
A TEAE is defined as any AE that starts on or after the dosing date, or (in the case of a continuing AE) worsens in severity during treatment relative to the pretreatment state.
All AEs are coded using MedDRA v17.0 or later and graded according to NCI-CTCAE v4.03.
Any patient with multiple occurrences of the same AE is counted only once in the AE category.
Abbreviations: AE, adverse event; MedDRA, Medical Dictionary for Regulatory Activities; NCI-CTCAE, National Cancer Institute Common Terminology Criteria for Adverse Events; TEAE, treatment-emergent adverse event; v, version.

1.2.2.1.2. Efficacy Results (13 January 2017 Data Cutoff)

Duration of Treatment:

As of a 13 January 2017 data cutoff, a total of 111 patients had received tislelizumab in Parts 1-3 of the Phase 1a component of Study BGB-A317_001.

Among the 22 patients treated in Part 1 of Phase 1a, the mean treatment duration was 109.2 days (range: 1 to 365 days). Treatment lasted longer for the 81 patients treated in Part 2 of Phase 1a, with mean durations of 142.4 days of treatment for patients receiving tislelizumab Q2W and 191.9 days of treatment for patients receiving tislelizumab Q3W (overall range: 1 to 471 days).

For the 8 patients treated in Part 3 of Phase 1a, the mean treatment duration was 54.1 days (range: 32 to 67 days).

Clinical Response:

Phase 1a

One of 22 (5%) patients in Part 1 of Phase 1a had a documented clinical response (a confirmed partial response [PR]); this patient was receiving tislelizumab 2 mg/kg. Meanwhile, 1/81 (1.2%) patients in Part 2 of Phase 1a had a documented complete response (CR) and 15/81 (18.5%) had a documented PR while receiving tislelizumab 2 mg/kg Q2W.

The overall clinical response rates in the Phase 1a component of Study BGB-A317_001 were 11.3% (95% CI: 4.66-21.89%) for the Q2W regimen (combining 2 mg/kg and 5 mg/kg dosing) and 20.4% (95% CI: 10.24-34.34%) for the Q3W regimen (combining 2 mg/kg and 5 mg/kg dosing).

Phase 1b

Observations in Patients with Hepatocellular Carcinoma

As of 28 April 2017, 40 patients with unresectable HCC were enrolled into Phase 1B of the Study and treated with tislelizumab Q3W at a dose of 5 mg/kg.

The median treatment duration was 64 days (range 1 – 471 days) and 25/40 patients remained on study treatment. Among 27 evaluable patients, 3 PRs were observed, with 2 confirmed responses and one awaiting confirmation. All responders remained on study treatment. Stable disease was observed in 9 patients. Disease control rate (PR + stable disease [SD]) was 44%.

The preliminary safety profile and antitumor activity support continued exploration and development of tislelizumab in patients with advanced or unresectable HCC.

1.2.3. Benefit-Risk Assessment

Patients with unresectable HCC represent a population with a great unmet medical need. Sorafenib is currently the only approved systemic therapy available to all HCC patients worldwide that has modest efficacy and a manageable safety profile (characterized by a relatively moderate incidence of dose reductions or drug discontinuations; Section 1.1.1).

Data from a Phase 1/2 clinical trial of nivolumab, an anti-PD-1 antibody, demonstrated an objective response of 20% (95% CI: 15-26) that is durable, thus suggesting the potential efficacy of anti-PD-1 antibody for the treatment of patients with advanced HCC (for additional discussion, see Section 1.1.1.1).

In a small cohort of pretreated patients with advanced HCC (n = 27 evaluable), all treated with tislelizumab 5 mg/kg in Study BGB-A317_001, PRs were observed in 3 patients (2 confirmed PRs, 1 unconfirmed PR). It is important to note that median duration of treatment in these patients was only 64 days (range: 1 to 471 days), and responses were assessed after every 9

weeks on therapy. Confirmation of response occurred at least 4 weeks after initial response assessment.

More than 400 patients have been treated with tislelizumab monotherapy at clinically relevant doses (≥ 2 mg/kg). The safety profile is consistent with known class effects of anti-PD-1 antibodies, and included mostly mild/moderate AEs. Very few severe (≥ Grade 3) irAEs have been observed; when they do occur, these events are generally reversible and manageable with study drug interruption and/or steroid treatment. For further discussion of the tislelizumab safety profile, please refer to the IB.

Given the unmet medical need and limited treatment options in this indication, the benefit/risk assessment based on available tislelizumab Phase 1 data and published data from the Phase 1/2 study of nivolumab is considered favorable. In order to assess the potential benefit and safety of BGB-A317 monotherapy over standard of care sorafenib, this randomized trial comparing tislelizumab monotherapy to sorafenib will be conducted.

An Independent Data Monitoring Committee (IDMC) will be established to regularly monitor the safety of tislelizumab when compared with sorafenib. An interim analysis for OS superiority test is planned (Section 9.11).

1.3. Study Conduct

This study will be conducted in compliance with the protocol approved by the Institutional Review Board (IRB) or Independent Ethics Committee (IEC) and in accordance with Good Clinical Practice (GCP) standards.

2. STUDY OBJECTIVES AND ENDPOINTS

2.1. Objectives

2.1.1. Primary Objectives

- To compare OS between tislelizumab and sorafenib as first-line treatment in patients with unresectable HCC

2.1.2. Secondary Objectives

- To compare ORR assessed by Blinded Independent Review Committee (BIRC) according to Response Evaluation Criteria in Solid Tumors [RECIST]) Version (v)1.1 between tislelizumab and sorafenib
- To compare progression-free survival (PFS) assessed by BIRC according to RECIST v1.1 between tislelizumab and sorafenib
- To compare DOR assessed by BIRC according to RECIST v1.1 between tislelizumab and sorafenib
- To compare time to progression (TTP) between tislelizumab and sorafenib
- To compare health-related quality of life (HRQoL) between tislelizumab and sorafenib
- To compare tumor assessment outcomes (ie, ORR, PFS, DOR, TTP) assessed by investigator according to RECIST v1.1 between tislelizumab and sorafenib
- To compare disease control rate (DCR) and clinical benefit rate (CBR), assessed by BIRC and investigator according to RECIST v1.1, between tislelizumab and sorafenib
- To compare safety and tolerability of tislelizumab versus sorafenib

2.1.3. Exploratory Objectives

- To explore potential predictive biomarkers, including but not limited to PD-L1 expression
- To characterize the PK of tislelizumab in patients with unresectable HCC
- To determine host immunogenicity to tislelizumab in patients with unresectable HCC

2.2. Endpoints

2.2.1.1. Primary Endpoints

- OS – defined as the time from the date of randomization to the date of death due to any cause

2.2.2. Secondary Endpoints

- ORR as assessed by BIRC – defined as the proportion of patients with a documented CR or PR per RECIST v1.1
- PFS – defined as the time from the date of randomization to the date of the first objectively documented tumor progression, assessed by BIRC per RECIST v1.1, or death, whichever occurs first
- DOR – defined as the time from the first determination of an objective response, assessed by BIRC per RECIST v1.1, until the first documentation of progression or death, whichever occurs first
- TTP – defined as the time from the date of randomization to the date of the first objectively documented tumor progression, assessed by BIRC per RECIST v1.1
- HRQoL – measured using European Organisation for Research and Treatment of Cancer Quality of Life Questionnaire-Hepatocellular Carcinoma 18 Questions (EORTC QLQ-HCC18) index-score, European Organisation for Research and Treatment of Cancer Quality of Life Questionnaire-Core 30 (EORTC QLQ-C30) index-score, and European Quality of Life 5-Dimensions, 5-level (EQ-5D-5L) scale
- Tumor assessment (ie, ORR, PFS, DOR and TTP), assessed by investigator per RECIST v1.1
- DCR – defined as the proportion of patients whose best overall response (BOR) is CR, PR, or SD, assessed by BIRC and investigator per RECIST v1.1
- CBR – defined as the proportion of patients who have CR, PR, or SD of ≥ 24 weeks in duration, assessed by BIRC and investigator per RECIST v1.1
- Safety assessment (eg, new AEs, AEs present at baseline that worsen in severity during the study, and clinical laboratory abnormalities)

2.2.3. Exploratory Endpoints

- PD-L1 expression and other potential predictive biomarkers
- Summary of serum concentrations of tislelizumab
- Assessments of immunogenicity of tislelizumab by determining the incidence of antidrug antibodies (ADAs)

3. STUDY DESIGN

3.1. Summary of Study Design

This is a randomized, open-label, multicenter Phase 3 study in adult patients with unresectable HCC. Patients must have progressed after loco-regional therapy or not be amenable to management with either surgery or loco-regional therapy. Patients must also have received no prior systemic therapy for HCC, in keeping with the first-line setting of this study. A substudy investigating the safety, tolerability, PK, and preliminary efficacy in Japanese patients is planned; preliminary safety and tolerability will be evaluated before Japanese patients are recruited in this Phase 3 study (see Appendix 13).

The study procedures will occur over a Screening phase (up to 28 days); Treatment phase (until intolerable toxicity, withdrawal of informed consent, or the time point at which, in the opinion of the investigator, the patient is no longer benefiting from study therapy); Safety Follow-up phase (up to 30 days following last study treatment or initiation of new anticancer therapy, whichever occurs first, for any AEs and up to 90 days following last dose of tislelizumab for irAEs, regardless of whether or not the patient starts a new anticancer therapy; and a Survival Follow-up phase (duration varying by patient).

A schedule of efficacy and safety assessments is presented in Appendix 1.

3.2. Study Schematic

The study design schematic is presented in Figure 1. Approximately 640 patients will be randomized 1:1 to receive (on an open-label basis) either tislelizumab or sorafenib.

Figure 1: Study Design Schematic

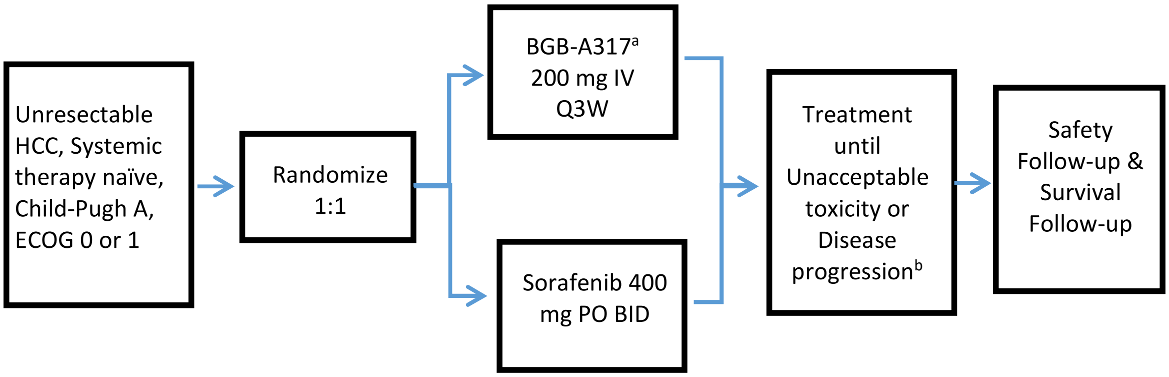

a. The initial infusion (Cycle 1, Day 1) will be administered over a period of 60 minutes. If this infusion is well tolerated, subsequent infusions may be administered over 30 minutes. After tislelizumab infusion, patients will be further monitored for a period of 2 hours during Cycles 1 and 2. From Cycle 3 onward, a postinfusion monitoring period of > 30 minutes will be required.
b. At the discretion of the investigator, patients may be treated beyond disease progression under protocol-defined conditions (Section 7.13.1).

Abbreviations: BID, twice daily; ECOG, Eastern Cooperative Oncology Group; HCC, hepatocellular carcinoma; IV, intravenously; PO, orally; Q3W, once every 3 weeks.

3.3. Duration of Study

Total duration of study participation will vary by patient. Each study phase is further discussed below.

3.3.1. Screening Phase

Screening evaluations will be performed within 28 days prior to randomization. Patients who agree to participate in this study will sign the informed consent form (ICF) prior to undergoing any Screening procedure (refer to Appendix 1 for details). Patients who are suspected or known to have serious respiratory concurrent illness or exhibit significant respiratory symptoms unrelated to underlying cancer will also take a pulmonary function test (refer to Section 7.1.4 and Appendix 1 for details). Screening evaluations may be repeated as needed within the Screening period; the investigator is to assess preliminary patient eligibility according to the latest Screening assessment results.

Archival tumor tissue is required to be obtained for biomarker analysis (except if not allowed by local regulations/IRBs/IECs). If archival samples are not available, collection of a fresh tumor biopsy at baseline is highly recommended if accessible. Refer to Section 7.6 for details.

3.3.2. Treatment Phase

After completing all Screening activities, patients confirmed eligible by the sponsor will be randomized in a 1:1 ratio to receive either tislelizumab or sorafenib treatment. Randomization will be stratified according to macrovascular invasion (present vs absent), extrahepatic spread (present vs absent), etiology (HCV vs other [includes HBV]), ECOG (0 vs. 1) and geography (Asia [excluding Japan] vs Japan vs Rest of World).

Patients with HBV and HCV co-infection will be grouped along with HBV into the “other” category of etiology for randomization. Patients will receive open-label treatment with 1 of the following:

- Arm A: tislelizumab 200 mg intravenously (IV) Q3W
- Arm B: Sorafenib 400 mg orally (PO) twice daily (BID)

All study treatment is to be continually administered until intolerable toxicity, withdrawal of informed consent, or the time point at which, in the opinion of the investigator, the patient is no longer benefiting from study therapy.

Treatment beyond the initial investigator-assessed, RECIST v1.1-defined disease progression is permitted in both treatment groups provided the patient meets the criteria described in Section 7.13.1. Radiological assessment of tumor response status should be performed every 9 weeks in Year 1 and every 12 weeks from Year 2 onwards. Tumor response will be assessed by BIRC and investigators. Details are provided in Section 7.4 and Section 10.1.

Safety will be assessed throughout the study by monitoring AEs/SAEs (toxicity grades assigned per National Cancer Institute Common Terminology Criteria for Adverse Events [NCI-CTCAE] Version [v]4.03), and laboratory abnormalities. Vital signs, physical examinations, ECOG performance status (PS) change, and electrocardiogram (ECG) results will also be used for safety assessment. Safety assessments are further detailed in Section 7.3 and the Schedule of Assessments (Appendix 1).

3.3.3. Safety Follow-up Phase

In both study arms, patients will return approximately 30 days after the last dose of study drug or before the initiation of a new anticancer therapy (whichever occurs first) for a Safety Follow-up Visit to collect AEs or SAEs that may have occurred after the patient discontinued from the study treatment. In the tislelizumab arm, all irAEs will be recorded up to 90 days after the last dose of tislelizumab, regardless of whether or not the patient starts a new anticancer therapy. In both treatment groups, all drug-related SAEs will be recorded by the investigator after treatment discontinuation until patient death or loss to follow-up, whichever occurs first.

3.3.4. Survival Follow-up Phase

Patients who discontinue study drug for reasons other than disease progression (eg, toxicity) or death will continue to undergo tumor assessments according to Section 7.4 and the Schedule of Assessments (Appendix 1), until the patient experiences disease progression, withdraws consent, loss to follow-up, death, or until the study terminates, whichever occurs first.

Patients will be followed for survival and further anticancer therapy information after discontinuation of study treatment via telephone calls, patient medical records, and/or clinic visits approximately every 3 months (± 14 days) until death, loss to follow-up, withdrawal of consent, or study termination by the sponsor (Section 7.12).

3.4. Study Rationales

3.4.1. Rationale for Tislelizumab in the Treatment of Hepatocellular Carcinoma

High levels of gamma Fc receptor (FcγR)-expressing myeloid-derived cells (eg, M2 macrophage, myeloid-derived suppressor cells [MDSC]) in tumor tissues predict a poor survival of tumor-bearing animals after anti-PD-1 monoclonal antibody treatment; this is possibly due to Fc-FcγR-mediated ADCC or antibody-dependent cellular phagocytosis (ADCP) depletion of effector T-cells (Gul 2015, Prieto 2015, Makarov-Rusher 2015, Beers 2016, Dahan 2015). As a no- to low-FcγR-binding agent (thus causing minimal ADCC/ADCP effect), tislelizumab is expected to show superior efficacy and lower toxicity in HCC patients compared to sorafenib.

Available data from a clinical trial with another anti-PD-1 monoclonal antibody, nivolumab, has shown the drug to have both a manageable safety profile and promising antitumor activity in patients with unresectable HCC (Section 1.1.1.1).

Finally, according to the latest data collected from the Phase 1 Study BGB-A317_001, tislelizumab monotherapy has established a manageable safety profile, with the most common side effects consistent with known class effects of other anti-PD-1 antibodies (Section 1.2.3).

3.4.2. Rationale for Selection of Tislelizumab Dose

The fixed dose of tislelizumab 200 mg Q3W was selected on the basis of available clinical data.

The safety of tislelizumab has been tested across a range of doses in Study BGB-A317_001 (0.5 mg/kg to 10 mg/kg Q2W [n = 62]; 2 mg/kg to 5 mg/kg Q3W [n = 41]) with no MTD defined at the highest dose examined. Efficacy has also been demonstrated in 23 of 266 (9%) evaluable patients to date, diagnosed with a variety of tumor types and treated according to a scheduled dose range. Specifically, rates of treatment-related AEs and SAEs observed in patients taking 2

mg/kg and 5 mg/kg Q2W and Q3W were comparable, suggesting no clear dose-dependence across these regimens.

Similarly, confirmed ORRs in patients treated with tislelizumab 2 mg/kg and 5 mg/kg Q2W ranged between 5 and 14%, compared to a range of 17 to 37% for patients treated at 2 mg/kg and 5 mg/kg Q3W.

According to Phase 1a component PK data, serum concentrations of tislelizumab showed linear relationships with doses ranging from 0.5 mg/kg Q2W to 10 mg/kg Q2W. Because the clearance of tislelizumab was found to be independent of body weight, a 200-mg dose (body-weight adjusted dose between 3 and 4 mg/kg) administered every 3 weeks was expected to lead to serum exposures that fall between those observed after 2 mg/kg and 5 mg/kg doses. This prediction was corroborated with simulations conducted using the population PK analysis and further supported by preliminary PK data from 5 patients who received 200 mg Q3W (Phase 1a, Part 3). tislelizumab concentrations after the first 200-mg dose were between the concentrations observed after 2 mg/kg and 5 mg/kg doses (in patients from Phase 1a, Parts 1 and 2). Additionally, as shown by available data from Studies BGB-A317_001 and BGB-A317-102, the PK profile of tislelizumab is consistent between Asian (Chinese) and Caucasian patients.

Additionally, no unexpected treatment-related AEs occurred in the 200-mg fixed dose cohort (Phase 1a, Part 3) when compared to body-weight-based cohorts. Of the evaluable patients treated (n = 4), 1 patient had a BOR of SD and 3 patients had BORs of progressive disease (PD). Therefore, clinical activity with a manageable and tolerable safety profile is expected to be maintained in patients receiving tislelizumab 200 mg Q3W.

In conclusion, tislelizumab 200 mg once Q3W is the recommended dose for this Phase 3 global study.

3.4.3. Rationale for Selection of Sorafenib as Comparator

Sorafenib is the only globally available, approved, systemic treatment to have demonstrated statistically significant improvement in OS and prolonged TTP among patients with advanced HCC in 2 large randomized trials (Cheng 2009, Llovet 2008), as discussed in Section 1.1.1.

Sorafenib is presently the standard of care worldwide for patients living with unresectable HCC. Therefore, this study will compare the clinical efficacy and safety of tislelizumab to sorafenib in this patient population.

4. MATERIALS AND METHODS

4.1. Selection of Study Population

The specific eligibility criteria for selection of the approximately 640 patients planned for randomization (1:1 ratio to tislelizumab or sorafenib) are provided in Sections 4.1.1 and 4.1.2. The sponsor will not grant any eligibility waivers.

4.1.1. Inclusion Criteria

To be eligible to participate in this study, a patient must meet all of the following criteria:

1. Is male or female, aged ≥ 18 years on the day the patient voluntarily agrees to participate in the study (or the legal age of consent in the jurisdiction in which the study is taking place)
2. Has a histologically confirmed diagnosis of HCC
3. Has either BCLC Stage C disease, or BCLC Stage B disease that is not amenable to or has progressed after loco-regional therapy, and is not amenable to a curative treatment approach (see Forner et al, 2010)
4. Has received no prior systemic therapy for HCC
   NOTE: Patients who have received prior local therapy (eg, TACE) are not excluded. Prior systemic treatment is allowed for HCC patients enrolled in the Safety Run-in Substudy in Japan (Appendix 13)
5. Has ≥ 1 measurable lesion as defined per RECIST v1.1, provided that:
   - The target lesion(s) selected have not been previously treated with local therapy OR
   - The target lesion(s) selected that are within the field of prior local therapy have subsequently progressed as defined by RECIST v1.1
6. Has Child-Pugh A classification for liver function assessed within 7 days of randomization (Appendix 2)
7. Has ECOG PS score ≤ 1
8. Has adequate organ function, as demonstrated by meeting all of the following clinical laboratory assessment criteria at Screening:
   - Absolute neutrophil count ≥ 1.5 × 10^9/L, platelets ≥ 75 × 10^9/L, and hemoglobin ≥ 85 g/L
     NOTE: Patients must not have required a transfusion of blood products and/or hematopoietic growth factors within the 14 days before sample collection
   - Estimated glomerular filtration rate (eGFR) > 30 mL/min/1.73 m^2 by Chronic Kidney Disease Epidemiology Collaboration equation (Appendix 8)
   - Serum albumin ≥ 29 g/L
   - Serum total bilirubin ≤ 51.3 µmol/L (3 mg/dl)

- Aspartate aminotransferase (AST) and alanine aminotransferase (ALT) both $\leq 5 \times$ ULN

9. If patient has HBV or HCV infection, meets the following criteria as applicable to the infection type:

   For patients with inactive/asymptomatic carrier, chronic, or active HBV:

   - Has HBV deoxyribonucleic acid (DNA) < 500 IU/mL (or 2500 copies/mL) at Screening
     NOTE: Patients with detectable hepatitis B surface antigen (HBsAg) or detectable HBV DNA should be managed per treatment guidelines. Patients receiving antivirals at Screening should have been treated for > 2 weeks prior to randomization and should continue treatment for 6 months after study drug treatment discontinues.

   For patients with HCV:

   - Infection is evidenced by detectable HCV ribonucleic acid (RNA)

10. If a female of childbearing potential (ie, physiologically capable of becoming pregnant), agrees to practice highly effective methods of birth control for the duration of the study and (for patients in Arm A) for > 120 days after the last dose of tislelizumab OR (for patients in Arm B) > 30 days after the last dose of sorafenib, and have a negative urine or serum pregnancy test within 7 days of the first study drug administration (see Appendix 6).

11. If a non-sterile male, agrees to practice highly effective methods of birth control for the duration of the study and (for patients in Arm A) for > 120 days after the last dose of tislelizumab OR (for patients in Arm B) > 14 days after the last dose of sorafenib.

4.1.2. Exclusion Criteria

To be eligible to participate in this study, a patient cannot meet any of the following exclusion criteria:

1. Has known fibrolamellar HCC, sarcomatoid HCC, or mixed cholangiocarcinoma and HCC histology
2. Has tumor thrombus involving main trunk of portal vein or inferior vena cava
3. Has received within 28 days before randomization loco-regional therapy to the liver (ie, TACE, transcatheter embolization, hepatic arterial infusion, radiation, radioembolization, or ablation)
4. Has received within 28 days before randomization any prior immunotherapy (eg, interleukin, interferon, thymoxin, et cetera) **or within 14 days** any Chinese herbal medicine or patent medicine used to control cancer (Appendix 11)
5. Has, at Screening, and/or has any prior history of ≥ Grade 2 hepatic encephalopathy (Appendix 2)
6. Has, at Screening, pericardial effusion, uncontrollable pleural effusion, or clinically significant ascites defined as meeting either of (a) detectable ascites on Screening physical examination OR (b) has at Screening, ascites requiring paracentesis

7. Has a history of severe hypersensitivity reaction to other monoclonal antibodies
8. Has, at Screening, or has had within 6 months before randomization any clinical evidence of portal hypertension with bleeding esophageal or gastric varices
9. Patients with toxicities which have not recovered to baseline or stabilized as a result of prior anticancer therapy, except alopecia
10. Has, at Screening, or has had within 6 months before randomization, any bleeding or thrombotic disorder or any prescribed anticoagulant requiring therapeutic international normalized ratio monitoring (eg, warfarin or similar agents)
11. Has, at Screening, or has had within the 2 years before randomization, any active malignancy, with the exception of the HCC under investigation in this trial and any locally recurring cancer that has been treated curatively (eg, resected basal or squamous cell skin cancer, superficial bladder cancer, carcinoma in situ of the cervix or breast)
12. Has, at Screening, any known central nervous system metastasis and/or leptomeningeal disease
13. Has, at Screening, any active immune deficiency or autoimmune disease and/or has a history of any immune deficiency or autoimmune disease that may relapse (Appendix 4)

    NOTE: Patients with the following diseases are not excluded:

    - Type 1 diabetes
    - Hypothyroidism (provided it is managed with hormone replacement therapy only)
    - Controlled celiac disease
    - Skin diseases not requiring systemic treatment (eg, vitiligo, psoriasis, alopecia)
    - Any other disease that is not expected to recur in the absence of external triggering factors
14. Has any condition that has required systemic treatment with either corticosteroids (> 10 mg daily of prednisone or equivalent) or other immunosuppressive medication within 14 days before randomization

    NOTE: Patients who are currently or have previously been on any of the following steroid regimens are not excluded:

    - Adrenal replacement steroid (dose ≤ 10 mg daily of prednisone or equivalent) in the absence of active autoimmune disease
    - Topical, ocular, intra-articular, intranasal, or inhalational corticosteroid with minimal systemic absorption
    - Short course of corticosteroid prescribed prophylactically (eg, for contrast dye allergy) or for the treatment of a non-autoimmune condition (eg, delayed-type hypersensitivity reaction caused by contact allergen)
15. Has any history of interstitial lung disease or non-infectious pneumonitis, unless induced by radiation therapy

16. Has, at Screening, any severe chronic or active infection (excluding viral hepatitis) requiring systemic antibacterial, antifungal, or antiviral therapy (eg, tuberculosis)
17. Has Screening ECGs with QT interval corrected for heart rate (QTc) (corrected by Fridericia's method) > 450 msec

    NOTE: If any patient has QTc > 450 msec on initial ECG, a follow-up ECG will be performed to confirm result.
18. Has any of the following cardiovascular risk factors:
    - Cardiac chest pain, defined as moderate pain that limits instrumental activities of daily living (ADL), within 28 days before randomization
    - Symptomatic pulmonary embolism within 28 days before randomization
    - Any history of acute myocardial infarction within 6 months before randomization
    - Any history of heart failure meeting New York Heart Association Classification III or IV (Appendix 7) within 6 months before randomization
    - Any event of ventricular arrhythmia > Grade 2 in severity within 6 months before randomization
    - Any history of cerebrovascular accident or transient ischemic attack within 6 months before randomization
19. Has a known history of human immunodeficiency virus
20. Has any underlying medical condition that, in the investigator's opinion, will make the administration of study treatment hazardous or potentially obscure the interpretation of AEs/toxicities
21. Has undergone prior allogeneic stem cell transplantation or organ transplantation
22. Has been administered a live vaccine within 4 weeks before randomization
    NOTE: Seasonal vaccines for influenza are generally inactivated vaccines and are allowed. Intranasal vaccines are live vaccines and are not allowed.
23. Has undergone any major surgical procedure within 28 days before randomization
24. Has any contraindication for sorafenib treatment (eg, severe hypersensitivity to sorafenib or any other component of NEXAVAR) NOTE: Contraindication for sorafenib treatment is not an exclusion from participation in the Safety Run-in Substudy in Japan (Appendix 13)
25. Female patients who are nursing

    Note : Female patients who suspend nursing are allowed. If the patient wishes to resume nursing after completion of treatment, time from the completion of study drug treatment until nursing resumption would be 90 days (>5 half-lives of tislelizumab).

5. STUDY TREATMENT

5.1. Formulation, Packaging, Handling, and Storage

5.1.1. Tislelizumab

Tislelizumab is a monoclonal antibody formulated for IV injection in a single-use vial (20R glass, United States Pharmacopeia [USP] type I), containing a total of 100 mg antibody in 10 mL of isotonic solution. Tislelizumab has been aseptically filled in single-use vials with a Flurotec-coated butyl rubber stopper and an aluminum cap. Each vial is packaged in a single carton box.

The label will include at a minimum, drug name, dose strength, contents, sponsor, protocol number, kit number, batch/lot number, directions for use, storage conditions, caution statements, retest or expiry date, and space to enter the patient number and name of investigator. The contents of the label will be in accordance with all applicable local regulatory requirements.

The study drug must be kept at the temperature condition as specified on the label. Tislelizumab must be stored at temperatures between 2°C and 8°C and protected from light. Refer to the Pharmacy Manual for details regarding IV administration, accountability, and disposal. Please also refer to the Investigator's Brochure for other details regarding tislelizumab.

The interactive response technology (IRT) system will be used for drug supply management. The study drug will be dispatched to a study center only after receipt of the required documents in accordance with applicable regulatory requirements and the sponsor's procedures. The investigator or pharmacist/designated personnel are responsible for maintaining the drug supply inventory and acknowledgment receipt of all study drug shipments. All study drug must be stored in a secure area with access limited to the investigator and authorized study center personnel and under physical conditions that are consistent with study drug-specific requirements.

Study drug must be dispensed or administered according to procedures described herein. Only patients enrolled in the study may receive study drug, in accordance with all applicable regulatory requirements. Only authorized study center personnel may supply or administer study drug.

5.1.2. Sorafenib

Sorafenib will be supplied as red, round, film-coated tablets, each containing 200 mg of sorafenib (as the tosylate); however, the actual appearance and composition of the product may depend on the respective marketed product sourced for the participating countries.

The label will include at a minimum, drug name, dose strength, contents, sponsor, protocol number, bottle number, lot number, directions for use, storage conditions, caution statements, retest or expiry date, and space to enter the patient number and name of the investigator. The contents of the label will be in accordance with all applicable local regulatory requirements.

The study drug must be kept at the temperature condition as specified on the label.

The IRT system will be used for drug supply management. The study drug will be dispatched to a study center only after receipt of the required documents in accordance with applicable

regulatory requirements and the sponsor's procedures. The investigator or pharmacist/designated personnel is responsible for maintaining the drug supply inventory and acknowledgment receipt of all study drug shipments. All study drug must be stored in a secure area with access limited to the investigator and authorized study center personnel and under physical conditions that are consistent with study drug-specific requirements.

Study drug must be dispensed or administered according to procedures described herein. Only patients enrolled in the study may receive study drug, in accordance with all applicable regulatory requirements. Only authorized study center personnel may supply or administer study drug.

For further details, see the manufacturer's local prescribing information for sorafenib.

5.2. Dosage, Administration, and Compliance

Dosing schedules for both arms, broken out by individual arm, are provided in Table 3. The first dose of study drug is to be administered within 2 days of randomization. All patients will be monitored continuously for AEs. Treatment modifications (eg, dose delay, reduction, interruption, or discontinuation) will be based on specific laboratory and AE criteria, as described in Section 5.5.

Table 3: Selection and Timing of Dose for Each Patient

Study Drug	Dose	Frequency of Administration	Route of Administration	Duration of Treatment
Tislelizumab	200 mg	Once every 3 weeks	Intravenous	Until intolerable toxicity, withdrawal of informed consent, or the time point at which, in the opinion of the investigator, the patient is no longer benefiting from study therapy
Sorafenib (NEXAVAR)	400 mg (2 × 200-mg tablets)	Twice daily	Orally	Until intolerable toxicity, withdrawal of informed consent, or the time point at which, in the opinion of the investigator, the patient is no longer benefiting from study therapy

In both arms, treatment beyond initial disease progression (as assessed by the investigator per RECIST v1.1) is permitted, provided that the patient meets the criteria in Section 7.13.1.

5.2.1. Tislelizumab

Tislelizumab 200 mg will be administered on Day 1 of each 21-day cycle (Q3W).

Tislelizumab will be administered by IV infusion, through an IV line containing a sterile, non-pyrogenic, low-protein-binding 0.2 or 0.22 micron in-line or add-on filter. Specific instructions for product preparation and administration are provided in the Pharmacy Manual.

As a routine precaution, after infusion of tislelizumab on Day 1 of Cycle 1 and Cycle 2, patients must be monitored for 2 hours afterwards in an area with resuscitation equipment and emergency

agents. From Cycle 3 onward, at least a 30-minute monitoring period is required in an area with resuscitation equipment and emergency agents.

The initial infusion (Cycle 1, Day 1) will be delivered over 60 minutes. If this is well tolerated, then the subsequent infusions may be administered over 30 minutes, which is the shortest time period permissible for infusion. tislelizumab must not be concurrently administered with any other drug (refer to Section 6).

Guidelines for dose modification, treatment interruption, or discontinuation and for the management of irAEs and infusion-related reactions are provided in detail in Section 5.5.1, Section 8.8.1, and Appendix 10.

Refer to the Pharmacy Manual for detailed instructions on drug preparation, storage, and administration.

5.2.2. Sorafenib

Patients randomized to the sorafenib arm will receive treatment with sorafenib, to be self-administered PO at the recommended dose of 400 mg (2 tablets of 200 mg) BID without food (at least 1 hour before or 2 hours after a meal). The tablets should be swallowed with a glass of water. Refer to the appropriate local prescribing information for additional details.

An adequate amount of supply of sorafenib will be dispensed to patients on Day 1 of each new cycle (Q3W). Each time study drug is dispensed, compliance will be evaluated and encouraged. Treatment compliance will also be monitored by drug accountability and recorded in the patient's medical record and electronic case report form (eCRF). If the number of tablets returned does not agree with the expected number, the patient should be counseled and proper dosing reinforced.

If a dose is missed, the patient should skip the missed dose and take the next dose as scheduled. A double dose should not be administered to make up for missed individual doses.

Patients will be monitored continuously for AEs and will be instructed to notify the investigator immediately for any and all AEs. Management of suspected adverse drug reactions may require temporary interruption and/or dose reduction of sorafenib therapy. Guidelines for dosage modification and treatment interruption or discontinuation are provided in Section 5.5.2.

5.3. Handling of Overdose

Any overdose (defined as ≥ 600 mg of tislelizumab in a 24-hour period) or incorrect administration of study drug should be noted in the patient's chart and on the appropriate eCRF. AEs associated with an overdose or incorrect administration of study drug will be recorded on the AE eCRF. Any SAEs associated with an overdose or incorrect administration are required to be reported within 24 hours of awareness via SAE reporting process as described in Section 8.7.2. Supportive care measures should be administered as appropriate.

5.4. Investigational Medicinal Product Accountability

The investigational medicinal products (IMPs) required for completion of this study (tislelizumab and sorafenib) will be provided by the sponsor, as required by local or country

specific guidance. The investigational site will acknowledge receipt of IMPs. Any damaged shipments will be replaced.

Accurate records of all IMP received, dispensed, returned, and disposed should be recorded on the site's Drug Inventory Log. Refer to the Pharmacy Manual for details of IMP management.

Compliance will be assessed by the investigator and/or study personnel at each patient visit and information provided by the patient.

The investigator and/or study personnel will keep accurate records of drug dispensed and used by each patient. This information must be captured in the source document at each patient visit. The investigator is responsible for tislelizumab and sorafenib, reconciliation, and record maintenance. In accordance with all applicable regulatory requirements, the investigator or designated study center personnel must maintain tislelizumab and sorafenib accountability records throughout the course of the study. This person will document the amount of tislelizumab and sorafenib received from the sponsor, the amount supplied, and/or administered to and returned by patients, if applicable.

5.5. Dose Modifications or Delays

Reasons for dose modifications or delays, the supportive measures taken, and the outcome will be documented in the patient's chart and recorded in the eCRF.

5.5.1. Dose Modification for Tislelizumab

There will be no dose reduction of tislelizumab in this study. Dose delays or interruption of < 12 weeks will be permitted. The investigator should make every effort to maintain dose intensity in patients.

Patients may temporarily suspend study treatment if they experience a toxicity that is considered related to tislelizumab and requires that a dose be withheld. Patients should resume tislelizumab treatment as soon as possible after the AE recovers to baseline or Grade 1 severity (whichever is more severe) within 12 weeks after the last dose of tislelizumab. If the patient is unable to resume tislelizumab in that timeframe, study treatment should be discontinued.

In case a patient is benefiting from the study treatment while meeting the discontinuation criteria, resumption of study treatment may occur upon discussion and agreement with sponsor medical monitor.

If the timing of a protocol-mandated study visit coincides with a holiday, weekend, or other event, the visit should be scheduled on the nearest feasible date (refer to the visit window in Appendix 1), with subsequent dosing continued on the original 21-day treatment interval schedule, with a minimum of 14 days between tislelizumab dosing.

Management guidelines for irAEs and infusion-related reactions in patients treated with tislelizumab are presented in Appendix 10 and Section 8.8.2, and Section 8.8.1, respectively.

5.5.2. Dose Modification for Sorafenib

Dose modifications for sorafenib will be performed according to the investigator's clinical judgement and consistent with the prescribing information, using the details in this section as guidance (see Nexavar PI, 2017).

Management of suspected adverse drug reactions may require temporary interruption and/or dose reduction of sorafenib therapy (Appendix 9). When dose reduction is necessary, the sorafenib dose may be reduced to 400 mg once daily. If additional dose reduction is required, sorafenib may be reduced to a single 400 mg dose every other day. Once the dose has been decreased, it should remain reduced for all subsequent administrations or further reduced if necessary. The dose of sorafenib may be increased after improvement of non-hematological adverse reactions if it is allowed per country specific labeling. Sorafenib treatment may be delayed up to 30 days, if the reason for the delay is toxicity/AE. After 30 days, treatment will be permanently discontinued. In exceptional cases where patients are responding, re-initiation of therapy after missing > 30 consecutive days of treatment may be done on a case by case basis after discussion with the sponsor medical monitor.

5.6. Disposal and Destruction

After completion of the study, all unused tislelizumab and sorafenib will be inventoried and packaged for return shipment by the hospital unit pharmacist or other designated study center personnel. After receiving written sponsor approval, the inventoried supplies can be destroyed on site or at the depot according to institutional policies.

6. PRIOR AND CONCOMITANT THERAPY

6.1. Prior Therapy

The exclusion criteria (Section 4.1.2) specify that patients will not have received prior systemic therapy for their HCC.

6.2. Concomitant Therapy

6.2.1. Permitted Therapy

Most concomitant medications and therapies deemed necessary in keeping with the local standards of medical care at the discretion of the investigator for the supportive care (eg, antiemetics, antidiarrheals) and in a patient's interest are allowed. All concomitant medications will be recorded on the eCRF including all prescription, and non-prescription medications, herbal supplements, IV medications and fluids. If changes (dose, stop, or start) in concomitant medication occur during the study, documentation of drug dosage, frequency, route, date, and reason for use will be recorded on the eCRF.

All concomitant medications received within 30 days before randomization and 30 days after the last infusion or dose of study treatment should be recorded.

Patients with active hepatitis B defined as either detectable HBsAg or HBV DNA at baseline must initiate treatment 2 weeks prior to randomization or first dose, and continue until 6 months after the last dose. Patients should continue effective antiviral treatment during the study to decrease potential viral re-activation risk. Tenofovir, and entecavir are recommended in the American Association for the Study of Liver Disease (AASLD) guideline because they lack resistance with long-term use (Terrault et al, 2016; AASLD/IDSA HCV Guidance Panel, 2015). The investigator might use other antiviral agents, if appropriate, following local guidelines. Management of antiviral therapy is at the discretion of the investigator; however, a reason must be provided if a patient with active hepatitis B is not treated with antiviral prophylaxis.

BeiGene does not require patients with active hepatitis C to receive treatment with antiviral therapy. Patients with detectable HCV RNA and who are receiving treatment at screening should remain on continuous, effective antiviral therapy during the study. Investigators can consider treatment with sofosbuvir alone or in combination with other antivirals following the AASLD guideline or the local guidelines as appropriate. However, interferon-based therapy for either HBV or HCV is not permitted on study. Patients who are given antiviral therapy must initiate treatment at least 2 weeks prior to randomization.

Systemic corticosteroids given for the control of irAEs must be tapered gradually (see Appendix 10) and be at non-immunosuppressive doses (≤ 10 mg/day of prednisone or equivalent) before the next tislelizumab administration. The short-term use of steroids as prophylactic treatments (eg, patients with contrast allergies to diagnostic imaging contrast dyes) is permitted.

Bisphosphonates and RANK-L inhibitors are allowed for bone metastases if initiated prior to enrollment and at a stable dose. Bisphosphonates are permitted during the trial for a non-malignant indication.

Palliative (limited-field) radiation therapy is permitted, but only for pain control or prophylaxis of bone fracture to sites of bone disease present at baseline provided the following criteria are met:

- Repeat imaging demonstrates no new sites of bone metastases
- The lesion being considered for palliative radiation is not a target lesion for RECIST v1.1
- The case is discussed with sponsor medical monitor to ensure study compliance

Additionally, palliative radiation or other focally ablative therapy for other non-target sites of the disease is permitted if clinically indicated per investigators' discretion and after consultation with the medical monitor.

Whenever possible, these patients should have a tumor assessment of the lesion(s) before receiving the radiotherapy in rule out progression of disease.

6.2.2. Excluded (Prohibited or Restricted) Therapy

6.2.2.1. Therapies Excluded During Tislelizumab Treatment

The following medications are prohibited at the time of Screening and during the administration of tislelizumab:

- Live vaccines within 28 days before the first dose of tislelizumab and 60 days following the last dose of tislelizumab

The following medications are restricted at the time of Screening and during the administration of tislelizumab:

- Immunosuppressive agents (except to treat a treatment-emergent drug-related AE)
- Systemic corticosteroids > 10 mg daily (prednisone or equivalent), except to treat or control a treatment-emergent drug-related AE or for short-term use as prophylactic treatment
- Herbal remedies with immunostimulating properties (ie, mistletoe extract) or that are known to potentially interfere with liver or other major organ functions (ie, hypericin). Patients must notify the investigator of all herbal remedies used during the study

6.2.2.2. Therapies Excluded During Sorafenib Treatment

For patients randomized to sorafenib treatment, the following medications are contraindicated; they are thus to be prohibited during the administration of sorafenib:

- Warfarin or similar agents that require therapeutic INR monitoring

For patients randomized to sorafenib treatment, the following medications are known to interfere; they are thus to be restricted during the administration of sorafenib:

- QT-prolonging agents: Sorafenib has been shown in some clinical studies to have a modest prolongation effect on QT interval/QTc (Kloth 2015). Monitor electrolytes

and ECGs in patients with concomitant drugs known to prolong the QT interval, including Class 1a and 3 antiarrhythmics.

- CYP3A4 inducers: Avoid concomitant use of strong CYP3A4 inducers (eg, carbamazepine, dexamethasone, phenobarbital, phenytoin, rifampin, rifabutin, St. John's wort), when possible, because these drugs can decrease the systemic exposure to sorafenib.

Note that it is not an exhaustive list of agents; local sorafenib prescribing information should be consulted for general guidance.

6.2.2.3. Therapies Excluded During All Study Treatment

The following medications are to be prohibited at the time of Screening and during the administration of study treatment:

- Any concurrent antineoplastic therapy (ie, chemotherapy, hormonal therapy, immunotherapy, or standard or investigational agents [including Chinese (and other Country) herbal medicine and patent medicines] for the treatment of cancer) is not allowed (Appendix 11)
- Extensive radiation therapy (except for local, palliative radiotherapy to bone; refer to Section 6.2.1) is not allowed

The following medications are to be restricted at the time of Screening and during the administration of study treatment:

- Use of potentially hepatotoxic drugs in patients with impaired hepatic function including Child-Pugh-A classification should be carefully monitored. Patients must notify the investigator of all concurrent medications used during the study

The following guidelines should be also followed during the study:

- With the exception of diagnostic biopsy of tumor tissue or placement of a venous access device, the investigator should discuss with the sponsor medical monitor any patient who requires surgery during the study
- Patients should avoid alcohol completely and should avoid other addictive drugs during the study

7. STUDY ASSESSMENTS AND PROCEDURES

A flowchart of scheduled study assessments is provided in Appendix 1. Patients will be closely monitored for safety and tolerability throughout the study. All assessments must be performed and documented in the medical record and eCRF for each patient.

Dosing will occur only if the clinical assessment and local laboratory test values (which must be available before any dosing) have been reviewed and found to be acceptable per-protocol guidelines.

If the timing of a protocol-mandated study visit coincides with a holiday, weekend, or other events, the visit should be scheduled on the nearest feasible date (the visit window is provided in Appendix 1), with subsequent dosing continued on the original 21-day treatment interval schedule, with a minimum of 14 days between tislelizumab dosing.

7.1. Screening

Screening evaluations will be performed within 28 days prior to randomization. Patients who agree to participate will sign the ICF prior to undergoing any Screening procedure. Patients who have a history of serious or severe pulmonary disease or are suspected to have serious or severe respiratory concurrent illness or exhibit significant respiratory symptoms should undergo pulmonary function tests (refer to Appendix 1 for details). Patients with electrolyte deficiencies, including magnesium, potassium, and calcium, must have these clinically corrected in the screening period before enrollment in the study. Screening evaluations may be repeated as needed within the Screening period; the investigator is to assess patient eligibility according to the latest Screening assessment results.

Results of standard of care tests or examinations performed prior to obtaining informed consent and ≤ 28 days prior to randomization may be used for the purposes of Screening rather than repeating the standard of care tests unless otherwise indicated.

Procedures conducted during the Screening Visit only are described in this section. For the description of other assessments that are conducted during Screening, as well as throughout the study, refer to the following sections: Safety Assessments (Section 7.3), Tumor and Response Evaluations (Section 7.4) and Biomarkers (Section 7.6).

Re-screening under limited conditions may be allowed after consultation with BeiGene, eg, when a patient narrowly misses a laboratory criterion and it's correctable and not due to rapidly deteriorating condition or PD. Re-screening is allowed only once.

7.1.1. Demographic and Medical History

Medical history should include any history of clinically significant disease, surgery, or cancer history (including prior anticancer therapies and procedures); reproductive status (ie, of childbearing potential or no childbearing potential [Section 7.1.2]); history of alcohol consumption and tobacco (ie, presence or absence); and all medications (eg, prescription drugs, over-the-counter drugs, herbal or homeopathic remedies, nutritional supplements) used by the patient within 30 days before randomization. If appropriate, clinically significant disease should be graded according to NCI-CTCAE v 4.03 and reported in the Medical History eCRF.

Demographic data will include age, gender, and self-reported race/ethnicity.

Cancer history will include an assessment of prior surgery, prior radiotherapy, prior drug therapy, including start and stop dates, best response and reason for discontinuation, and collecting presence or absence of macrovascular invasion and/or extrahepatic spread. Information on radiographic studies prior to study entry may be collected for review by the investigator.

7.1.2. Females of Childbearing Potential and Contraception

Childbearing potential is defined as being physiologically capable of becoming pregnant. Refer to Appendix 6 for contraception guidelines and definitions of "women of childbearing potential" and "no childbearing potential"

7.1.3. Informed Consent Form and Screening Log Completion

Voluntary, written informed consent for participation in the study must be obtained before performing any study-specific procedures.

The ICFs for enrolled patients and for patients who are screened but not enrolled will be maintained at the study site.

All Screening evaluations must be completed and reviewed to confirm that patients meet all eligibility criteria before randomization. Details of the process for the review of eligibility criteria prior to randomization will be defined in a separate screening and randomization process document to ensure consistency of the review of eligibility requirements. The investigator will maintain a Screening log to record details of all patients screened and to confirm eligibility or record reasons for Screening failure, as applicable.

7.1.4. Pulmonary Function Tests

Patients who are suspected or known to have serious/severe respiratory conditions or exhibit significant respiratory symptoms unrelated to the underlying cancer will undergo pulmonary function testing which may include but is not limited to spirometry and assessment of diffusion capacity done during the Screening period to assist the determination of suitability on the study.

At least 50% of performance expected by age is required for spirometry (including forced vital capacity and forced expiratory volume in the first second of expiration). Assessment of diffusion capacity during Screening is required in patients with a history of thoracic radiotherapy or restrictive lung disease. When diffusing capacity for carbon monoxide is performed, at least 60% of performance expected by age is required.

7.2. Enrollment

7.2.1. Confirmation of Eligibility

The investigator will assess and the sponsor, or designee, will confirm the eligibility of each patient. All Screening procedure results and relevant medical history must be available before eligibility can be determined. All inclusion criteria must be met and none of the exclusion criteria may apply. No eligibility waivers will be granted.

7.2.2. Patient Numbering

After obtaining informed consent, study site personnel will access the IRT system to assign a unique patient number to a potential study participant.

7.2.3. Randomization

Site personnel will access the IRT system to assign study drugs. Study treatment must commence within 2 days after randomization/treatment assignment. At randomization, patients will be stratified by the following 5 factors:

- Macrovascular invasion (present vs absent)
- Extrahepatic spread (present vs absent)
- ECOG (0 vs 1)
- Etiology (HCV vs other [includes HBV])
- Geography (Asia [excluding Japan] vs Japan vs Rest of World)

Patients with HBV and HCV co-infection will be grouped along with HBV into the "other" category of etiology for randomization.

7.2.4. Tislelizumab and Sorafenib Dispensation

Tislelizumab and sorafenib will be dispensed and administered as described in Section 5.2

7.3. Safety Assessments

7.3.1. Vital Signs

Vital signs collected on study will include measurements of pulse rate and blood pressure (systolic and diastolic) while the patient is in a seated position after resting for 10 minutes, as well as body temperature (°C).

For the first infusion of tislelizumab, the patient's vital signs will be assessed within 60 minutes before, during, and 30 minutes after the infusion of tislelizumab. For subsequent infusions, vital signs will be collected within 60 minutes before infusion and if clinically indicated, during and 30 minutes (up to + 15 minutes) after the infusion. Patients will be informed about the possibility of delayed post infusion symptoms and instructed to contact the investigator if they develop such symptoms. Refer to Section 8.8.1 regarding management of infusion-related reactions.

For patients randomized to the sorafenib arm, monitor blood pressure weekly during the first 6 weeks after the first dose. Thereafter, monitor blood pressure at Day 1 of every cycle. Investigators may perform additional or more frequent blood pressure assessments if clinically indicated.

7.3.2. Physical Examinations

A complete physical examination, including an evaluation of the head, eyes, ears, nose, throat, cardiovascular, dermatological, musculoskeletal, respiratory, gastrointestinal, and neurological systems is required to be performed at Screening. Any abnormality identified during Screening will be graded according to NCI-CTCAE v 4.03 and recorded on the Clinically Significant

Symptoms at Baseline eCRF with appropriate disease/condition terms. Height (baseline only) and weight will be measured and recorded in the eCRF.

At subsequent visits (or as clinically indicated), limited, symptom-directed physical examinations will be performed. Changes from baseline will be recorded in patient notes and recorded on eCRF where appropriate/indicated. New or worsened clinically significant abnormalities are to be recorded as AEs on the eCRF. Refer to Section 8.4.1. regarding AE definitions and reporting and follow-up requirements.

7.3.3. Eastern Cooperative Oncology Group Performance Status Grading

Each patient's ECOG PS (Table 4) will be assessed at the Screening Visit, pretreatment on Day 1 of each treatment cycle, End of Treatment Visit and Safety Follow-up Visit.

Table 4: Eastern Cooperative Oncology Group Performance Status – Grading System

Grade	Performance Status
0	Fully active; able to carry on all predisease performance without restriction
1	Restricted in physically strenuous activity but ambulatory and able to carry out work of a light or sedentary nature, eg, light house work, office work
2	Ambulatory and capable of all self-care but unable to carry out any work activities; up and about more than 50% of waking hours
3	Capable of only limited self-care; confined to bed or chair for more than 50% of waking hours
4	Completely disabled; cannot carry on any self-care; totally confined to bed or chair
5	Dead

7.3.4. Laboratory Safety Tests

Laboratory assessments on serum chemistry, hematology, coagulation, and urinalysis will be conducted, of which certain elements will be collected as specified in Appendix 5.

If laboratory tests at Screening (hematology, chemistry, coagulation, urinalysis, and alpha fetoprotein) are not performed within 7 days prior to randomization, these tests should be repeated and reviewed before study drug administration. Hematology and serum chemistry (including liver function tests) should be performed weekly for the first 3 cycles and at the beginning of subsequent cycles (data collected as specified in Appendix 5). After Cycle 1, results are to be reviewed within 48 hours before study drug administration.

Furthermore, the following tests will be performed as specified in Appendix 1:

- Urine pregnancy test, performed at each visit prior to dosing; a serum pregnancy test must be performed if the urine pregnancy test is positive or equivocal
- Thyroid function testing (thyroid stimulating hormone [TSH], free T3, free T4)
- Hepatitis serology and viral load
  - HBsAg, antibodies against HBsAg, antibodies against hepatitis B core antigen [HBcAb])
  - HCV serology (anti-HCV antibody)

  - HBV DNA and HCV RNA
- Alpha fetoprotein biomarker assessment

Details about sample collection and shipment will be provided in a separate instruction manual. Investigators in regions where central laboratories are required, may also use results from local laboratories for assessing eligibility, safety monitoring and dosing decisions.

7.3.5. Electrocardiograms

12-lead ECG recordings are required at Screening, Safety Follow-up, and as clinically indicated. All ECG recordings should be performed after the patient has been resting for at least 10 minutes, and a repeat ECG should be performed to confirm findings, if any. Additional ECG monitoring should be performed following local guidance based on the sorafenib labeling (see Nexavar PI, 2017). Abnormal ECGs should be repeated in triplicate.

7.3.6. Adverse Events

Adverse events will be graded and recorded throughout the study according to NCI-CTCAE, version 4.03 (NCI-CTCAE, June 2010). Characterization of toxicities will include severity, duration, and time to onset.

All AEs, including SAEs, will be collected as described in Section 8.5. At the end of treatment, ongoing AEs considered related to study treatment will be followed until the event has resolved to baseline or ≤ Grade 1, the event is assessed by the investigator as stable, the patient is lost to follow-up, the patient withdraws consent.

7.3.7. Ophthalmologic Examination

Ophthalmologic examination, as described below, will be performed on all patients during Screening, and subsequently throughout the study only for those patients randomized to receive tislelizumab.

Eye exam, visual acuity test, and optical coherence tomography (or equivalent diagnostic test) will be assessed by an ophthalmologist at Screening. Eye exam, visual acuity test, and optical coherence tomography (or equivalent diagnostic test for retinal examination) captured as standard of care prior to obtaining written informed consent and within 28 days of randomization may be used for the Screening evaluation. Patients will undergo repeat assessments by an ophthalmologist approximately every 15 weeks (± 7 days) during study treatment and a final assessment < 30 days after the last dose of study treatment.

In addition, investigators should solicit patients regarding changes in vision, visual disturbance, or ocular inflammation at each scheduled study visit during study treatment. For any change in vision, referral to an ophthalmologist will be made for further management guidance (see Appendix 10).

7.4. Tumor and Response Evaluations

Tumor imaging will be performed within 28 days prior to the first study treatment. Results of standard of care tests or examinations (Appendix 1) performed prior to obtaining informed consent and ≤ 28 days prior to study entry may be used for the purposes of Screening rather than

repeating the standard of care tests. During the study, tumor imaging will be performed approximately every 9 weeks (± 7 days) in Year 1 and every 12 weeks (± 7 days) from Year 2 onwards. Investigators may perform additional assessments if clinically indicated.

Screening assessments and each subsequent assessment must include computed tomography (CT) scans (with oral/IV contrast, unless contraindicated) or magnetic resonance imaging (MRI) of the chest, abdomen, and pelvis. MRI (or CT scan if MRI is contraindicated or not readily available) of the head at baseline (≤ 28 days of before randomization) may be required at screening based on clinical judgement. Other known or suspected sites of disease must be included in the imaging assessments (neck, brain, etc.). MRI may be used when it is the standard of care at a site, regardless of whether or not CT is contraindicated.

The liver should be imaged using multi-phasic scans as much as possible, including late arterial phase and portal venous phase (and equilibrium phase, if consistent with local standard of care). Every effort should be made to keep the methodology consistent across visits for a subject (ie, phases acquired, timing for each phase, etc).

If a patient is known to have a contraindication to CT contrast media or develops a contraindication during the trial, a non-contrast CT of the chest plus a contrast-enhanced MRI (if possible) of abdomen and pelvis should be performed. If a CT scan for tumor assessment is performed in a positron emission tomography (PET)/CT scanner, the CT acquisition must be consistent with the standards for a full-contrast diagnostic CT scan.

Bone scans (Technetium-99m [TC-99m]) or ^{18}F-sodium fluoride-positron emission tomography (^{18}F-NaF PET) should be performed at Screening if clinically indicated. If bone metastases are present at Screening and cannot be seen on subsequent CT or MRI scans, or if clinically indicated, TC-99m or ^{18}F-NaF PET bone scans should be repeated when either progression in bone or a CR in the target lesion is suspected. CT scans of the neck or extremities should also be performed if clinically indicated and followed throughout the study, if there is evidence of metastatic disease in these regions at Screening. At the investigator's discretion, other methods of assessment of target lesion and non-target lesions per RECIST v1.1 may be used.

For subsequent tumor assessments, the same radiographic procedure used to assess disease sites at Screening are required to be used throughout the study (eg, the same contrast protocol for CT scans). Imaging of the head is not required during subsequent tumor exams except if new central nervous system disease is suspected. All known sites of disease must be documented at Screening and reassessed at each subsequent tumor evaluation.

Tumor response will be assessed by the investigator using RECIST v1.1 (Appendix 3) and also by the BIRC (see Section 10.1). The same evaluator should perform assessments, if possible, to ensure internal consistency across visits.

At the investigator's discretion, radiographic scans should be repeated at any time if PD is suspected.

Patients who discontinue study treatment early for reasons other than disease progression (eg, toxicity) or death will continue to undergo tumor assessments following the original plan until the patient experiences disease progression, withdraws consent, loss to follow-up, death, or until the study terminates, whichever occurs first.

Patients who continue tislelizumab/sorafenib treatment beyond radiographic disease progression (Section 7.13.1) will be monitored with a follow-up scan no more than 6 to 8 weeks beyond the initial diagnosis of radiographic PD before discontinuation of tislelizumab/sorafenib treatment. For patients who continue treatment based on investigator assessment of clinical benefit, tumor assessment will continue until treatment discontinuation.

Tumor assessments are required to be performed on schedule regardless of whether study treatment has been administered or held.

7.5. Pharmacokinetic and Antidrug Antibody Testing

Pharmacokinetic samples will be collected in patients receiving tislelizumab at the time points presented in Appendix 1. Procedures for collection of PK samples are described in the Laboratory Manual.

Tislelizumab may elicit an immune response. Patients with signs of any potential immune response to tislelizumab will be closely monitored.

Validated Screening and confirmatory assays will be employed to detect ADAs at multiple time points throughout the study (Appendix 1). The immunogenicity evaluation will utilize a risk-based immunogenicity strategy (Bai 2012, Worobec 2004) to characterize ADA responses to tislelizumab in support of the clinical development program.

The following assessments will be performed at a central laboratory:

- ADA assays: serum samples will be tested for the presence of ADAs to tislelizumab using a validated immunoassay
- PK assay: serum samples will be assayed for tislelizumab concentration with use of a validated immunoassay

Shipping, storage, and handling of samples for the assessment of tislelizumab PK and ADA assays will be managed through a central laboratory. Instruction manuals and supply kits will be provided for all central laboratory assessments.

7.6. Biomarkers

Shipping, storage, and handling of archival tumor, fresh tumor, and leftover tumor tissue for the assessment of biomarkers will be managed through a central laboratory. Refer to the Laboratory Manual for details of sample handling.

Archival tumor tissues (formalin-fixed paraffin-embedded [FFPE] block with tumor tissue or approximately 10 [≥ 5] unstained slides) are required (except if not allowed by local regulations/IRBs/IECs) to be sent to central laboratory for central immunohistochemistry assay of PD-L1 status. In addition to PD-L1 expression, other exploratory predictive biomarkers, such as tumor infiltrating lymphocytes, tumor mutation analysis, and gene expression profiling, that are related to response or clinical benefit of tislelizumab may also be evaluated.

In the absence of archival tumor tissues, a fresh biopsy of a tumor lesion at baseline is optional (written informed consent is required prior to fresh tumor biopsies). For fresh biopsy specimens, acceptable samples include core needle biopsies for deep tumor tissue or excisional, incisional, punch, or forceps biopsies for cutaneous, subcutaneous, or mucosal lesions.

Tumor tissue should be of good quality based on total and viable tumor content. Fine needle aspiration, brushing, cell pellets from pleural effusion, and lavage samples are not acceptable.

7.7. Health-Related Quality of Life Assessment

Patients will be asked to complete the EORTC QLQ-C30, EORTC QLQ-HCC18, and EQ-5D-5L questionnaires, before any clinical activities that are performed that day, during on-study clinic visits according to the schedule in Appendix 1. The questionnaires will be provided in the patient's preferred language.

7.8. Visit Windows

All visits must occur within ± 3 days from the scheduled date, unless otherwise noted (see Appendix 1). All assessments will be performed on the day of the specified visit unless an acceptable time window is specified. Assessments scheduled on the day of study treatment administration (Day 1) of each cycle should be performed prior to study treatment infusion/dose unless otherwise noted. Laboratory results are required to be reviewed prior to dosing.

If the timing of a protocol-mandated study visit coincides with a holiday, weekend, or other events, the visit should be scheduled on the nearest feasible date (the visit window is provided in Appendix 1), with subsequent dosing continued on the original 21-day treatment interval schedule, with a minimum of 14 days between tislelizumab dosing.

7.9. Unscheduled Visits

Unscheduled visits may be performed at any time at the patient's or investigator's request and may include vital signs/focused physical examination; ECOG performance status; AE review; concomitant medications and procedures review; radiographic assessments; physical examination of liver, spleen, and lymph nodes; disease-related constitutional symptoms; and hematology and chemistry laboratory assessments. The date and reason for the unscheduled visit must be recorded in the source documentation.

If an unscheduled visit is necessary to assess toxicity or for suspected disease progression, then diagnostic tests may be performed based on investigator assessment as appropriate, and the results of these tests should be entered on the unscheduled visit eCRF.

7.10. End of Treatment Visit

The End of Treatment Visit is conducted when the investigator determines that tislelizumab or sorafenib will no longer be used. If routine laboratory tests (eg, hematology, serum chemistry) are completed within 7 days before the End of Treatment Visit, tests need not be repeated. Tumor assessment is not required at the End of Treatment Visit provided that fewer than 6 weeks have passed since the last assessment.

7.11. Safety Follow-up Visit

Patients who discontinue treatment for any reason will be asked to return to the clinic for the Safety Follow-up Visit (to occur within 30 days [± 7 days]) after the last study treatment. In the situation where study treatment is withheld/interrupted for 30 days or more prior to permanent discontinuation of study treatment (End of Treatment visit), the Safety Follow-up Visit should be

conducted at the same time as the End of Treatment Visit. Common procedures to both visits should be performed only once.

In addition, telephone contacts with patients should be conducted to assess AEs and concomitant medications (if appropriate, ie, associated with an AE or is a new anticancer therapy) at 60, and 90 days (±14 days) after the last dose of tislelizumab, whether or not the patient starts a new anticancer therapy. AEs and SAEs are collected up to 30 days after the last dose of study drug or initiation of new anticancer therapy, whichever occurs first, and irAEs (tislelizumab only) are collected up to 90 days after the last dose of study drug. Beyond 90 days, investigators should continue to report any SAEs or AEs that are believed to be related to study drug(s) if they become aware of them.

The End of Treatment Visit at which a response assessment showed PD, resulting in patient discontinuation, may be used as the Safety Follow-up Visit, provided the End of Treatment Visit occurred 30 days (± 7 days) after the last study treatment. Patients who discontinue study treatment prior to disease progression will have their tumors assessed as outlined in Section 7.4.

See the study flowchart provided in Appendix 1 for assessments to be performed at the Safety Follow-up Visit.

7.12. Survival Follow-up

Following discontinuation of the study treatment, all patients will be followed for survival status beginning 3 months after the Safety Follow-up Visit or as directed by the sponsor. Information on Survival Follow-up and the subsequent anticancer treatment will be collected via telephone calls, patient medical records, and/or clinic visits approximately every 3 months (± 14 days) until death, loss to follow-up, withdrawal of consent, or study termination by the sponsor.

7.13. Patient, Treatment, Study, and Site Discontinuation

Patients who discontinue study treatment, but who have not withdrawn consent for follow-up, should be followed for assessments of antitumor activity (Section 7.4), safety (Section 7.11), and survival (Section 7.12), if possible.

7.13.1. Discontinuation of Study Treatment

Patients have the right to voluntarily withdraw from the study or discontinue study treatment at any time for any reason. In addition, the investigator has the right to discontinue a patient from the study treatment at any time. Reasons of a patient may be discontinued from the study treatment may include, but are not limited to the following:

- Patient withdrawal of consent at any time
- Any medical condition that the investigator or sponsor determines may jeopardize the patient’s safety, if he or she were to continue in the study
- Investigator or sponsor determines it is in the best interest of the patient
- Patient noncompliance

Every effort should be made to obtain information on patients who discontinue from study treatment. The primary reason for discontinuation should be documented on the appropriate eCRF.

Patients must discontinue study treatment if they experience any of the following:

- Symptomatic deterioration (eg, uncontrollable pain secondary to disease or unmanageable ascites, etc.) attributed to disease progression
- Intolerable toxicity related to tislelizumab or sorafenib, including development of an immune mediated or sorafenib-mediated AE determined by the investigator to be unacceptable given the individual patient's potential response to therapy and severity of the event (see Appendix 9 and Appendix 10)
- If tislelizumab dosing had to be delayed > 12 weeks (see Section 5.5.1) or if sorafenib dosing had to be delayed > 30 days (see Section 5.5.2)
- Any medical condition that may jeopardize the patient's safety if he or she continues on study treatment
- Use of another non-protocol anticancer therapy (Section 6.2.2)
- Pregnancy

Patients will be permitted to continue tislelizumab if pseudo-progression is suspected and/or there is a reasonable belief that the patient could derive benefit from tislelizumab after RECIST v1.1 criteria for PD are met, and they must meet all of the following criteria:

- Absence of clinical symptoms and signs of disease progression (including clinically significant worsening laboratory values)
- ECOG PS ≤1
- Absence of rapid progression of disease or of progression at a critical anatomical site (eg, progression of a spinal lesion with impending cord compression) or that necessitates urgent alternative medical intervention

Patients with radiographic disease progression that is confirmed at a subsequent tumor assessment may be considered for continued tislelizumab treatment at the discretion of the investigator if they continue to meet the criteria above and have evidence of clinical benefit.

Patients will be permitted to continue sorafenib beyond initial investigator-assessed RECIST v1.1-defined progression if the patient has investigator-assessed clinical benefit and is tolerating study drug, and if they meet all of the criteria as listed above as the criteria to be considered for the continuation of tislelizumab when pseudo-progression is suspected.

The decision to continue tislelizumab /sorafenib beyond initial investigator-assessed progression must be discussed with the sponsor medical monitor and documented in the study records. The patient must sign an ICF for continued treatment beyond initial investigator-assessed progression.

If the decision is made to continue sorafenib treatment beyond initial progression, the patient will remain on the trial and will continue to be treated and monitored according to the schedule in

Appendix 1. Sorafenib treatment must be discontinued permanently upon documentation of further progression, either symptomatic or radiographic.

The primary reason for study drug discontinuation will be documented in the medical chart and on the appropriate eCRF. Patients who discontinue study drug prior to disease progression will not be replaced.

7.13.2. Study Termination and Study Site Closure

Study termination is defined as the time point when data collection will stop and the final analysis of the study will occur. The primary analyses will be conducted when the predefined death events have been observed (Section 9.5.1.1) for the efficacy and safety evaluation. The study will continue until the last patient has died, becomes lost to follow-up, or withdraws from study, or until sponsor decides to terminate the study.

The sponsor has the right to terminate this study at any time. Reasons for early termination of the study may include, but are not limited to, the following:

- The incidence or severity of AEs in this or other studies indicates a potential health hazard to patients
- Overall patient enrollment is unsatisfactory

The sponsor will notify each investigator if a decision is made to terminate the study. Should this be necessary, prematurely discontinued patients should be seen as soon as possible for an End of Treatment Visit and have end of treatment assessments performed.

The investigators may be informed of additional procedures to be followed to ensure that adequate consideration is given to the protection of the patient’s interests. The investigators will be responsible for informing IRBs/ IECs of the early termination of the trial.

Patients assigned to tislelizumab, who in the opinion of the investigator, continue to benefit from tislelizumab at study termination, will be offered the option to continue on treatment in a BeiGene clinical trial until it is commercially available in the country.

The sponsor has the right to close a site at any time. The decision will be notified to the site in advance. Reasons for closing a site may include, but are not limited to, the following:

- Excessively slow recruitment
- Poor protocol adherence
- Inaccurate, untimely, or incomplete data recording
- GCP noncompliance
- Study activity is completed (ie, all patients have completed and all obligations have been fulfilled)

8. SAFETY MONITORING AND REPORTING

The investigator is responsible for the monitoring and documentation of events meeting the criteria and definition of an AE or SAE as provided in this protocol

8.1. Risks Associated with Tislelizumab

Tislelizumab is an investigational agent that is currently in clinical development. Limited safety data are available in patients and the full safety profile has not been characterized. The following information is based on results from nonclinical and clinical studies with tislelizumab and published data on other molecules within the same biologic class.

The PD-L1/PD-1 pathway is involved in peripheral immune tolerance; therefore, such therapy may increase the risk of irAEs, specifically the induction or enhancement of autoimmune conditions. Immune-related AEs commonly associated with anti-PD-1 therapy are presented in Table 7.

The AEs believed to be immune-related and which have been observed in Phases 1a and 1b of the ongoing Study BGB-A317_001 are listed in Table 2.

Although most irAEs observed with immunomodulatory agents have been mild and self-limiting, such events should be recognized early and treated promptly to avoid potential major complications. Guidance for evaluation and management for suspected irAEs are provided in Section 8.8.2 and Appendix 10.

8.2. Risks Associated with Sorafenib

The most notable acute AEs observed with sorafenib have included diarrhea, fatigue, hand-foot skin reactions, and hypertension. The majority of these AEs have been mild to moderate in severity and manageable. Although not life-threatening, these AEs can severely impact the physical, psychological, and social well-being of patients receiving sorafenib and can lead to dose reductions and discontinuations.

Some toxicities are rare but can be fatal, including cardiovascular events, arterial thromboembolic events, and bleeding. See the local sorafenib product label for more information on toxicities (see Appendix 14).

8.3. General Plan to Manage Safety Concerns

8.3.1. Eligibility Criteria

Eligibility criteria were selected to guard the safety of patients in this trial. Results from the nonclinical toxicology studies and clinical data with tislelizumab, as well as the nonclinical/clinical data from other PD-L1/PD-1 inhibitors, were taken into account. Specifically, patients at risk for study-emergent active autoimmune diseases, or with a history of autoimmune diseases that may relapse, patients who have undergone allogenic stem cell or organ transplantation and patients who have received a live viral vaccine within 28 days before randomization are excluded from the study. Patients with contraindications for sorafenib treatment are also excluded from the study (see Section 4.1.2 for the full list of exclusion criteria).

8.3.2. Safety Monitoring Plan

Safety will be evaluated in this study through the monitoring of all serious and nonserious adverse events, defined and graded according to NCI-CTCAE v4.03. Patients will be assessed for safety (including laboratory values) according to the schedule in Appendix 1. Clinical laboratory results must be reviewed prior to the start of each cycle.

In this study, all enrolled patients will be evaluated clinically and with standard laboratory tests before and at regular intervals during their participation in this study. Safety evaluations will consist of medical interviews, recording of AEs, physical examinations, laboratory measurements (eg, hematology, chemistry) and other assessments. In addition, patients will be closely monitored for the development of any signs or symptoms of autoimmune conditions and infection.

Serum samples will be drawn from patients randomized to the tislelizumab arm for the determination of ADAs to tislelizumab. Administration of tislelizumab will be performed in a setting where emergency medical equipment and staff who are trained to respond to medical emergencies are available (see Section 5.2.1). Patients with contradictions for sorafenib treatment will not be enrolled on study.

All AEs will be recorded during the trial (AEs from the time of the first dose and SAEs from the time of signing of informed consent) and for up to 30 days after the last dose of study treatment (tislelizumab or sorafenib) or until the initiation of another anticancer therapy, whichever occurs first. At the end of treatment, ongoing AEs considered related to study treatment will be followed until the event has resolved to baseline or ≤ Grade 1, the event is assessed by the investigator as stable, the patient is lost to follow-up, the patient withdraws consent, or it has been determined that study treatment or participation is not the cause of the AE.

In the tislelizumab group, all irAEs will be recorded up to 90 days after the last dose of tislelizumab, whether or not the patient starts a new anticancer therapy. In both treatment groups, all drug-related SAEs will be recorded by the investigator after treatment discontinuation until patient death, withdrawal of consent, or loss to follow-up, whichever occurs first.

Investigators are instructed to report all events (including AEs and pregnancy-related AEs).

The potential safety issues anticipated in this trial, as well as measures intended to avoid or minimize such toxicities, are outlined in the following sections.

8.4. Adverse Events

8.4.1. Definition of Adverse Events

An AE is defined as any unfavorable and unintended sign (including an abnormal laboratory finding), symptom, or disease (new or exacerbated) temporally associated with the use of a study drug, whether considered related to study drug or not.

Examples of AEs include:

- Worsening of a chronic or intermittent pre-existing condition including an increase in severity, frequency, duration, and/or has an association with a significantly worse outcome

- New conditions detected or diagnosed after study drug administration, even though it may have been present before the start of the study
- Signs, symptoms, or the clinical sequelae of a suspected interaction
- Signs, symptoms, or the clinical sequelae of a suspected overdose of either study drug or a concurrent medication (overdose per se should not be reported as an AE or SAE)

When an AE or SAE occurs, it is the responsibility of the investigator to review all documentation (eg, hospital medical notes, laboratory results, and diagnostics reports) relative to the AE or SAE. The investigator will then record all relevant information regarding an AE or SAE in the eCRF. However, there may be instances when copies of medical records for certain cases are requested by the sponsor. In this instance, all patient identifiers will be blinded (redacted) on the copies of the medical records prior to submission to the sponsor.

8.4.2. Assessment of Severity

The investigator will make an assessment of severity for each AE and SAE reported during the study. All AEs and SAEs are to be assessed and graded based upon NCI-CTCAE v4.03.

Toxicities that are not specified in the NCI-CTCAE v4.03 will be defined as follows:

- Grade 1: Mild; asymptomatic or mild symptoms; clinical or diagnostic observations only; intervention not indicated
- Grade 2: Moderate; minimal, local or noninvasive intervention indicated; limiting age-appropriate instrumental ADL
- Grade 3: Severe or medically significant but not immediately life-threatening; hospitalization or prolongation of hospitalization indicated; disabling; limiting self-care ADL
- Grade 4: Life-threatening consequences; urgent intervention indicated
- Grade 5: Death related to AE

NOTE: The terms “severe” and “serious” are not synonymous. Severity is a measure of intensity (for example, grade of a specific AE, mild [Grade 1], moderate [Grade 2], severe [Grade 3], or life-threatening [Grade 4]), whereas seriousness is classified by the criteria based on the regulatory definitions. Seriousness serves as the guide for defining regulatory reporting obligations from the sponsor to applicable regulatory authorities as described in Section 8.7.2.3.

8.4.3. Assessment of Causality

The investigator is obligated to assess the relationship between the study drug and the occurrence of each AE or SAE, using best clinical judgement. Alternative causes, such as natural history of the underlying diseases, concomitant therapy, other risk factors, and the temporal relationship of the AE or SAE to the study drug should be considered and investigated. The investigator will also consult the IB and/or Product Information, for marketed products, in the determination of his/her assessment.

There may be situations when an SAE has occurred and the investigator has only limited information to include in the initial report to the sponsor. However, it is very important that the

investigator always assesses causality for every SAE prior to transmission of the SAE report to the sponsor, since the causality assessment is one of the criteria used when determining regulatory reporting requirements. The investigator may subsequently change his/her opinion of causality considering follow-up information and may amend the SAE report accordingly.

The causality of each AE should be assessed and classified by the investigator as “related” or “not related” based on all information available at the time of reporting. An AE is considered related if there is “a reasonable possibility” that the AE may have been caused by the study drug (ie, there are facts, evidence, or arguments to suggest possible causation). A number of factors should be considered in making this assessment, including:

- Temporal relationship of the AE to the administration of study treatment/study procedure
- Whether an alternative etiology has been identified
- Mechanism of action of the study drug
- Biological plausibility

An AE should be considered ‘related’ to study drug if any of the following criteria are met, otherwise the event should be assessed as not related:

- There is clear evidence to suggest a causal relationship, and other possible contributing factors can be ruled out
- There is evidence to suggest a causal relationship, and the influence of other factors is unlikely
- There is some evidence to suggest a causal relationship (eg, the AE occurred within a reasonable time after administration of the study drug). However, the influence of other factors may have contributed to the AE (eg, the patient’s clinical condition or other concomitant AEs).

8.4.4. Following Adverse Events

After the initial AE or SAE report, the investigator is required to proactively follow each patient and provide further information to the sponsor on the patient’s condition.

All AEs and SAEs documented at a previous visit/contact and designated as ongoing will be reviewed at subsequent visits/contacts.

All AEs and SAEs will be followed until resolution, the condition stabilizes or is considered chronic, the patient is lost to follow-up, or the patient withdraws consent. Once resolved, the appropriate AE or SAE eCRF page(s) will be updated. The investigator will ensure that follow-up includes any supplemental investigations as may be indicated to elucidate the nature and/or causality of the AE or SAE. This may include additional laboratory tests or investigations, histopathological examinations, radiographic imaging, or consultation with other health care professionals.

The sponsor may request that the investigator perform or arrange for the conduct of supplemental measurements and/or evaluations to elucidate as fully as possible the nature and/or causality of the AE or SAE. The investigator is obligated to assist. If a patient dies during participation in the

study or during a recognized follow-up period, the sponsor will be provided with a copy of any postmortem findings, including histopathology.

New or updated information should be reported to the sponsor according to the SAE instructions provided by the sponsor within the time frames outlined in Section 8.7.2.1.

8.4.5. Laboratory Test Abnormalities

Abnormal laboratory findings (eg, clinical chemistry, hematology, coagulation, or urinalysis) or other abnormal assessments (eg, ECGs, X-rays, or vital signs) that are judged by the investigator as clinically significant will be recorded as AEs or SAEs if they meet the definition of an AE (as defined in Section 8.4) or an SAE (as defined in Section 8.5). Clinically significant abnormal laboratory findings or other abnormal assessments that are detected during the study or are present at baseline and significantly worsen following the start of the study will be reported as AEs or SAEs. However, clinically significant abnormal laboratory findings or other abnormal assessments that are associated with the disease being studied, unless judged by the investigator as more severe than expected for the patient’s condition, or that are present or detected at the start of the study and do not worsen, will not be reported as AEs or SAEs.

8.5. Definition of a Serious Adverse Event

An SAE is any untoward medical occurrence that, at any dose:

- Results in death
- Is life-threatening

  NOTE: The term “life-threatening” in the definition of “serious” refers to an AE in which the patient was at risk of death at the time of the AE. It does not refer to an AE, which hypothetically might have caused death, if it were more severe.
- Requires hospitalization or prolongation of existing hospitalization

  NOTE: In general, hospitalization signifies that the patient was admitted (usually involving at least an overnight stay) to the hospital or emergency ward for observation and/or treatment that would not have been appropriate in the physician’s office or outpatient setting.
- Results in disability/incapacity

  NOTE: The term disability means a substantial disruption of a person’s ability to conduct normal life functions. This definition is not intended to include experiences of relatively minor medical significance, such as uncomplicated headache, nausea, vomiting, diarrhea, influenza, and accidental trauma (eg, sprained ankle), which may interfere or prevent everyday life functions, but do not constitute a substantial disruption.
- Is a congenital anomaly/birth defect
- Is considered a significant medical AE by the investigator based on medical judgement (eg, may jeopardize the patient or may require medical/surgical intervention to prevent one of the outcomes listed above)

The following are NOT considered SAEs:

- Hospitalization for elective treatment of a pre-existing condition that did not worsen from baseline
- Hospitalization for social/convenience considerations
- Scheduled therapy for the target disease of the study, including admissions for transfusion support or convenience

8.6. Suspected Unexpected Serious Adverse Reaction

A suspected unexpected serious adverse reaction (SUSAR) is a serious adverse reaction that is both unexpected (ie, not present in the product's Reference Safety Information [RSI]) and meets the definition of a serious adverse drug reaction, the specificity or severity of which is not consistent with those noted in the Investigator's Brochure.

8.7. Timing, Frequency, and Method of Capturing Adverse Events

8.7.1. Adverse Event Reporting Period

After informed consent has been signed but prior to the administration of the study drug, only SAEs should be reported.

After initiation of study drug, all AEs and SAEs, regardless of relationship to study drug, will be reported until either 30 days after last dose of study treatment (including sorafenib) or initiation of new anticancer therapy, whichever occurs first. In the tislelizumab arm, all irAEs should be reported until 90 days after the last dose of tislelizumab, regardless of whether or not the patient starts a new anticancer therapy. After this period, the investigator should continue to report any SAEs or AEs of special interest that are believed to be related to tislelizumab treatment.

8.7.2. Reporting Serious Adverse Events

8.7.2.1. Prompt Reporting of Serious Adverse Events

As soon as the investigator determines that an AE meets the protocol definition of an SAE, the event must be reported promptly (within 24 hours) to the sponsor or designee as described in Table 5.

Table 5: Timeframes and Documentation Methods for Reporting Serious Adverse Events to the Sponsor or Designee

	Timeframe for Making Initial Report	Documentation Method	Timeframe for Making Follow-up Report	Documentation Method	Reporting Method
All SAEs	Within 24 h of first knowledge of the SAE	SAE report	As expeditiously as possible	SAE report	Email or fax SAE form or Pregnancy form

Abbreviations: AE, adverse event; h, hours; SAE, serious adverse event.

8.7.2.2. Completion and Transmission of the Serious Adverse Event Report

Once an investigator becomes aware that an SAE has occurred in a patient, he/she is to report the information to the sponsor within 24 hours as outlined above in Section 8.7.2.1. The SAE report will always be completed as thoroughly as possible with all available details of the event, and forwarded to the sponsor or designee within the designated time frames.

If the investigator does not have all information regarding an SAE, he/she is not to wait to receive additional information before notifying the sponsor or designee of the SAE and completing the form. The form will be updated when additional information is received.

The investigator must always provide an assessment of causality for each SAE as described in Section 8.4.3.

The sponsor will provide contact information for SAE receipt.

8.7.2.3. Regulatory Reporting Requirements for Serious Adverse Events

The investigator will promptly report all SAEs to the sponsor in accordance with the procedures detailed in Section 8.7.2.1. The sponsor has a legal responsibility to notify, as appropriate, both the local regulatory authority and other regulatory agencies about the safety of a product under clinical investigation.

The investigator, or responsible person according to local requirements, will comply with the applicable local regulatory requirements related to the reporting of SAEs to regulatory authorities and the IRB/IEC.

All SUSARs (as defined in Section 8.6) will be submitted to all applicable regulatory authorities and investigators for tislelizumab studies.

When a study center receives an initial or follow-up safety report or other safety information (eg, revised Investigator’s Brochure) from the sponsor, the investigator or designated responsible person is required to promptly notify his/her IRB or IEC. The investigator should place copies of Safety Reports from the sponsor in the Investigator Site File.

8.7.3. Eliciting Adverse Event Information

The investigator or designee will ask about AEs by asking the patient the following standard questions:

- “How are you feeling?”
- “Have you had any medical problems since your last visit?”
- “Have you taken any new medicines (prescribed, over-the-counter, herbal or other supplements) since your last visit?”

8.7.4. Recording Diagnosis Versus Signs and Symptoms

If a diagnosis is known at the time of reporting, this should be recorded in the eCRF (and SAE report, as applicable), rather than the individual signs and symptoms (eg, record only “hepatitis” rather than “elevated transaminases, bilirubin” or “jaundice”).

However, if a constellation of signs and/or symptoms cannot be medically characterized as a single diagnosis or syndrome at the time of reporting, each individual AE should be recorded as an SAE or AE on the eCRF (and SAE report, if applicable). If a diagnosis is subsequently established, it should replace the individual signs and/or symptoms as the AE term on the eCRF (and SAE report, if applicable).

8.7.5. Recording Adverse Events Occurring Secondary to Other Events

In general, AEs occurring secondary to other AEs (eg, clinical sequelae or a cascade of AEs) should be identified by their primary cause. For example, if severe vomiting is known to result in dehydration, it is sufficient to record only vomiting as the SAE or AE on the eCRF (and SAE report, if applicable). However, if a patient initially has a nonserious AE, and it subsequently becomes an SAE, both AEs should be reported separately on the eCRF. The onset date of the nonserious AE should be recorded as the start date of the nonserious AE. The onset date of the SAE should be recorded as the start date when the nonserious AE becomes an SAE.

8.7.6. Recording Persistent or Recurring Adverse Events

A persistent AE is an AE that extends continuously, without resolution, between patient evaluation time points. Such AEs should only be recorded once with the worst CTCAE grading on the AE eCRF (and SAE report, if applicable).

A recurrent AE is an AE that occurs and resolves between patient evaluation time points, and subsequently recurs. All recurrent AEs should be recorded separately on the eCRF (and SAE report, if applicable).

8.7.7. Recording Disease Progression

Disease progression is expected in this study population, and the term “disease progression” should not be reported as an AE term. When disease progression is identified, the AE that identifies the disease progression should be reported as the AE term. For instance, a patient with pleural effusion presents with shortness of breath. The cause of the shortness of breath is a pleural effusion resulting from disease progression. The AE term should be reported as “pleural effusion” instead of disease progression or metastasis to lungs.

If a patient has a seizure that is determined to be associated with a brain metastasis, the term “seizure” should be recorded as the AE instead of disease progression or brain metastasis. If a patient experienced multi-organ failure due to disease progression, the term “multi-organ failure” should be reported as the AE instead of disease progression.

Deaths that are assessed by the investigator as solely due to disease progression should be recorded on Study Completion or Early Discontinuation eCRF as efficacy data. They should not be reported as an SAE. A patient death not solely due to disease progression as assessed by the investigator should be reported as an SAE immediately, regardless of relationship to study drug.

If there is any uncertainty regarding whether an AE is due to disease progression, it should be reported as an AE.

8.7.8. Recording Deaths

When recording a death as an SAE, the AE that caused or contributed to fatal outcome should be recorded as the single medical concept. If the cause of death is unknown and cannot be ascertained at the time of reporting, record "unexplained death."

8.7.9. Recording Pregnancies

If a female patient or the partner of a male patient becomes pregnant while receiving investigational therapy or within 120 days after the last dose of tislelizumab or within 30 days after the last dose of sorafenib, a pregnancy report form is required to be completed and expeditiously submitted to the sponsor to facilitate outcome follow-up. Information on the status of the mother and child will be forwarded to the sponsor. Generally, follow-up will be no longer than 6 to 8 weeks following the estimated delivery date. Any premature termination of the pregnancy will be reported.

While pregnancy itself is not considered to be an AE, any pregnancy complication or elective termination of a pregnancy for medical reasons will be recorded as an AE or SAE.

An abortion, whether accidental, therapeutic, or spontaneous, should be always reported as an SAE. Similarly, any congenital anomaly/birth defect in a child born to a patient exposed to the study drug should be recorded and reported as an SAE.

8.7.10. Recording Post-Study Adverse Events

A post-study AE or SAE is defined as any AE that occurs outside of the AE/SAE reporting period that is defined in Section 8.7.1.

Investigators are not obligated to actively seek AEs or SAEs in former patients. However, if the investigator learns of any SAE, including a death, at any time after a patient has been discharged from the study, and he/she considers the SAE related to the study drug, the investigator will notify the sponsor.

8.7.11. Expedited Reporting to Health Authorities, Investigators, Institutional Review Boards, and Independent Ethics Committees

The sponsor will promptly assess all SAEs against cumulative study drug experience to identify and expeditiously communicate new safety findings to regulatory authorities, investigators, IRBs, and IECs based on applicable legislation.

To determine the reporting requirements for individual SAEs, the sponsor will assess the expectedness of the SAEs using the following RSI documents:

- Tislelizumab Investigator's Brochure
- Local prescribing information for sorafenib

8.7.12. Assessing and Recording Immune-Related Adverse Events

Since treatment with anti-PD-1 therapy can cause autoimmune disorders, AEs considered by the investigator to be immune-related (see Section 8.8.2) should be classified as irAEs and identified as such in the eCRF AE page until Day 90, after treatment discontinuation.

Investigators should consult the guidance on diagnostic evaluation and management of irAEs, which are commonly seen with immune checkpoint inhibitors, in Appendix 10.

An extensive list of potential irAEs appears Section 8.8.2. All conditions similar to those listed should be evaluated to determine whether they are irAEs, based on a similar diagnostic process to those reactions that are presented in more detail in Appendix 10.

8.8. Management of Adverse Events of Special Interest

As a routine precaution, after infusion of tislelizumab on Day 1 of Cycle 1 and Cycle 2, patients must be monitored for at least 2 hours afterwards in an area with resuscitation equipment and emergency agents. From Cycle 3 onward, at least a 30-minute monitoring period is required in an area with resuscitation equipment and emergency agents.

The management of infusion-related reactions, severe hypersensitivity reactions, irAEs, and hepatic AEs are outlined below.

8.8.1. Infusion-Related Reactions

The symptoms of infusion-related reactions include fever, chills/rigor, nausea, pruritus, angioedema, hypotension, headache, bronchospasm, urticaria, rash, vomiting, myalgia, dizziness or hypertension. Severe reactions may include acute respiratory distress syndrome, myocardial infarction, ventricular fibrillation, and cardiogenic shock. Patients should be closely monitored for such reactions. Immediate access to an Intensive Care Unit (ICU) or equivalent environment and appropriate medical therapy (including epinephrine, corticosteroids, IV antihistamines, bronchodilators, and oxygen) must be available to treat infusion-related reactions.

Treatment modification for symptoms of infusion-related reactions due to tislelizumab are summarized in Table 6.

Table 6: Treatment Modification Guidelines for Symptoms of Infusion-Related Reactions Due to Tislelizumab

NCI-CTCAE Grade – severity	Guideline for Modification of Tislelizumab Treatment
**Grade 1 – mild**<br>Mild transient reaction; infusion interruption not indicated; intervention not indicated.	Decrease infusion rate by 50%. Any worsening is closely monitored. Medical management as needed.<br>Subsequent infusions should be given after premedication and at the reduced infusion rate.
**Grade 2 – moderate**<br>Therapy or infusion interruption indicated but responds promptly to symptomatic treatment (eg, antihistamines, NSAIDs, narcotics, IV fluids); prophylactic medications indicated for ≤ 24 hours.	Stop infusion. Infusion may be resumed at 50% of previous rate once infusion-related reactions has resolved or decreased to at least Grade 1 in severity. Any worsening is closely monitored. Proper medical management should be instituted as described below.<br>Subsequent infusions should be given after premedication and at the reduced infusion rate.

NCI-CTCAE Grade – severity	Guideline for Modification of Tislelizumab Treatment
**Grade 3 – severe**<br>Prolonged (eg, not rapidly responsive to symptomatic medication and/or brief interruption of infusion); recurrence of symptoms following initial improvement; hospitalization indicated for clinical sequelae.	Immediately stop the infusion. Proper medical management should be instituted as described below.<br>The patient should be withdrawn from study drug(s) treatment.
**Grade 4 – life-threatening**<br>Life-threatening consequences; urgent intervention indicated.	Immediately stop the infusion. Proper medical management should be instituted as described below.<br>The patient should be withdrawn from study drug(s) treatment.<br>Hospitalization is recommended.

Abbreviations: IV, intravenous; NCI-CTCAE, National Cancer Institute Common Terminology Criteria for Adverse Events; NSAID, nonsteroidal anti-inflammatory drug.

Once the tislelizumab infusion rate has been decreased by 50% or suspended due to an infusion-related reaction, it must remain decreased for all subsequent infusions with premedication. If the patient has a second infusion-related reaction (≥ Grade 2) on the slower infusion rate, infusion should be discontinued and the patient should be withdrawn from tislelizumab treatment.

NCI-CTCAE Grade 1 or 2 infusion reaction: Proper medical management should be instituted, as indicated per the type of reaction. This includes but is not limited to an antihistamine (eg, diphenhydramine or equivalent), antipyretic (eg, paracetamol or equivalent), and if considered indicated oral or IV glucocorticoids, epinephrine, bronchodilators, and oxygen. In the next cycle, patients should receive oral premedication with an antihistamine (eg, diphenhydramine or equivalent) and an antipyretic (eg, paracetamol or equivalent), and they should be closely monitored for clinical signs and symptoms of an infusion reaction.

NCI-CTCAE Grade 3 or 4 infusion reaction: Proper medical management should be instituted immediately, as indicated per type and severity of the reaction. This includes but is not limited to oral or IV antihistamine, antipyretic, glucocorticoids, epinephrine, bronchodilators, and oxygen.

Severe Hypersensitivity Reactions and Flu-Like Symptoms

If hypersensitivity reaction occurs, the patient must be treated according to the best available medical practice as described in the complete guideline for emergency treatment of anaphylactic reactions according to the Working Group of the Resuscitation Council (UK) (Soar, 2008). Patients should be instructed to report any delayed reactions to the investigator immediately.

In the event of a systemic anaphylactic/anaphylactoid reaction (typically manifested within minutes following administration of the drug/antigen, and characterized by: respiratory distress; laryngeal edema; and/or intense bronchospasm; and often followed by vascular collapse or shock without antecedent respiratory difficulty; cutaneous manifestations such as pruritus and urticaria with/without edema; and gastrointestinal manifestations such as nausea, vomiting, crampy abdominal pain, and diarrhea), the infusion must be immediately stopped and the patient discontinued from the study.

The patients will be administered epinephrine injection and dexamethasone infusion if hypersensitivity reaction is observed and then the patient should be placed on monitor immediately and ICU should be alerted for possible transfer if needed.

For prophylaxis of flu-like symptoms, a dose of 25 mg indomethacin or a comparable dose of nonsteroidal anti-inflammatory drugs (ie, 600 mg ibuprofen, 500 mg naproxen sodium) may be administered 2 hours before and 8 hours after the start of each dose of study drugs(s) infusion. Alternative treatments for fever (ie, paracetamol) may be given to patients at the discretion of the investigator.

8.8.2. Immune-Related Adverse Events

Immune-related AEs are of special interest in this study. If the events listed below or similar events occur, the investigator should exclude alternative explanations (eg, combination drugs, infectious disease, metabolic, toxin, disease progression or other neoplastic causes) with appropriate diagnostic tests, which may include but is not limited to serologic, immunologic, and histologic (biopsy) data. If alternative causes have been ruled out; the AE required the use of systemic steroids, other immunosuppressants, or endocrine therapy and is consistent with an immune mediated mechanism of action, the irAE indicator in the eCRF AE page should be checked.

A list of potential irAEs is shown below in Table 7. All conditions similar to those listed should be evaluated in patients receiving tislelizumab to determine whether they are immune-related.

Recommendation for diagnostic evaluation and management of irAEs is based on European Society for Medical Oncology (ESMO) and American Society of Clinical Oncology (ASCO) guidelines (Haanen 2017, Brahmer 2018) and common immune-related toxicities are detailed in Appendix 10.

For any AEs not included in Appendix 10, please refer to the ASCO Clinical Practice Guideline (Brahmer 2018) for further guidance on diagnostic evaluation and management of immune-related toxicities.

Table 7: Immune-Related Adverse Events Associated with Anti-PD-1 Drugs

Body System Affected	Events
Skin (mild-common):	pruritus or maculo-papular rash; vitiligo
Skin (moderate):	follicular or urticarial dermatitis; erythematous/lichenoid rash; Sweet's syndrome
Skin (severe-rare):	full-thickness necrolysis/Stevens-Johnson syndrome
Gastrointestinal:	colitis (includes diarrhea with abdominal pain or endoscopic/radiographic evidence of inflammation); pancreatitis; hepatitis; aminotransferase (ALT/AST) elevation; bowel perforation
Endocrine:	thyroiditis, hypothyroidism, hyperthyroidism; hypophysitis with features of hypopituitarism, eg, fatigue, weakness, weight gain; insulin-dependent diabetes mellitus; diabetic ketoacidosis; adrenal insufficiency
Respiratory:	pneumonitis/diffuse alveolitis
Eye:	episcleritis; conjunctivitis; iritis/uveitis

Body System Affected	Events
Neuromuscular:	arthritis; arthralgia; myalgia; neuropathy; Guillain-Barré syndrome; aseptic meningitis; myasthenic syndrome/myasthenia gravis; meningoencephalitis; myositis
Blood:	anemia; leukopenia; thrombocytopenia
Renal:	interstitial nephritis; glomerulonephritis; acute renal failure
Cardiac:	pericarditis; myocarditis; heart failure

Abbreviations: ALT, alanine aminotransferase; AST, aspartate aminotransferase; PD-1, programmed cell death protein-1.

Recommendations for managing irAEs are detailed in Appendix 10.

If a toxicity does not resolve to ≤ Grade 1 within 12 weeks, study drug(s) should be discontinued after consultation with the sponsor. Patients who experience a recurrence of any event at the same or higher severity grade with rechallenge should permanently discontinue treatment.

8.8.3. Hepatic and Renal Function Abnormalities

Patients with advanced HCC generally have underlying cirrhosis with decreased hepatic function. Special attention is needed because they may also have a concomitant chronic viral infection. Therefore, when a hepatic event, such as liver function laboratory abnormalities, is observed, the investigator must evaluate for re-activation of viral hepatitis, consider other drug-related toxicity, and exclude PD involving the liver. For diagnosis and management of patients with AST or ALT values ≤ Grade 1 at baseline, please see Section 8.8.2 and refer to Appendix 10.

In patients with Grade 2 AST/ALT abnormalities at baseline, therapeutic interventions with a steroid treatment may be required with rising AST and ALT laboratory abnormalities. The following algorithm is proposed for the use of steroid treatment:

- If AST or ALT increases by ≥ 50% relative to baseline and lasts for at least 1 week, start oral prednisolone 1 mg/kg/day and taper over at least 2-4 weeks; re escalate dose if liver function tests (LFTs) worsen, depending on clinical judgement (manage as per Appendix 10). Study treatment should be held until AST/ALT increase resolved/improved to baseline and prednisolone tapered to ≤ 10 mg.
- If any ALT or AST increases meets Grade 3 criteria, initiate steroid therapy promptly per Appendix 10. Study treatment will be held until AST or ALT improves to value ≤ Grade 2. Study drug may be reintroduced only after discussion with the sponsor.
- If any ALT or AST increases meets Grade 4 criteria, initiate steroid therapy promptly per Appendix 10. Study treatment will be discontinued permanently.

Renal Function Abnormalities

Patients with moderate renal dysfunction (estimated glomerular filtration rate > 30 mL/min/1.73 m^2 and < 60mL/min/1.73 m^2 by Chronic Kidney Disease Epidemiology Collaboration equation) may be enrolled into the study. The following algorithm is proposed for the use of steroid treatment in the management of irAEs:

- If the serum creatinine is normal at baseline, please see Section 8.8.2 and refer to Appendix 10 for diagnosis and management of patients with abnormal renal laboratory values.
- If the serum creatinine is Grade 1 at baseline and increase in serum creatinine meets criteria for serum creatinine increase ≥ Grade 2 after starting treatment with tislelizumab, refer to Appendix 10 for diagnosis and management of patients with abnormal renal laboratory values. Check the estimated GFR (eGFR) using Appendix 8 and the eGFR calculator link. In the setting of a Grade 2 serum creatinine increase only, study treatment can continue unless the serum creatinine increases by at least 50% from the baseline value OR the eGFR falls below 20 mL/min/1.73 m^2.
- If the serum creatinine is Grade 2 at baseline and increase in serum creatinine meets criteria for serum creatinine increase ≥ Grade 3 after starting treatment with tislelizumab, refer to Appendix 10 for diagnosis and management of patients with abnormal renal laboratory values. In the setting of a Grade 3 serum creatinine increase only, study treatment will be held until serum creatinine improves to baseline and treatment may resume only after discussion with sponsor medical monitor.

9. STATISTICAL METHODS AND ANALYSIS PLAN

The statistical analyses will be performed by the sponsor or designee after the data collection for the primary efficacy and safety analyses are completed and the database is locked and released. Data will be listed and summarized using SAS® Version 9.3 or higher (SAS Institute, Inc., Cary, North Carolina) per sponsor agreed reporting standards, where applicable. Details of the statistical analyses will be included in a separate Statistical Analysis Plan (SAP).

The statistical methods described in this section cover the summary of data collected from all randomized patients in the study.

9.1. Analysis Sets

Intent-to-Treat (ITT) Analysis Set: Includes all randomized patients. Patients will be analyzed according to their randomized treatment arms. This will be the primary analysis set for all efficacy analysis.

Per-Protocol (PP) Analysis Set: Includes randomized patients who received at least 1 dose of the assigned study drug and had no major protocol deviations. Major protocol deviations will be determined and documented before the database lock for the primary analysis.

Safety Analysis Set: Includes all patients who received at least 1 dose of study drug and is the primary analysis set used for all safety analyses.

9.2. Patient Disposition

The number of patients randomized, treated, discontinued from study drug and/or study and those with major protocol deviations will be counted. The primary reason for study drug and/or study discontinuation will be summarized according to the categories in the eCRF. The end of study status (alive, dead, withdrew consent, or lost to follow-up) at the data cutoff date will be summarized using the data from the eCRF.

Major protocol deviations will be summarized and listed by each category.

9.3. Demographic and Other Baseline Characteristics

Demographic and other baseline characteristics of the ITT analysis set will be summarized using descriptive statistics. Continuous variables include age, weight, vital signs, time since initial cancer diagnosis, and time since advanced disease diagnosis; categorical variables include gender, ECOG PS, geographical region, country, race, Child-Pugh classification, hepatitis virus infection status, BCLC staging, loco-regional procedures (present vs absent), metastatic site, macrovascular invasion, and extrahepatic spread status.

9.4. Prior and Concomitant Medications

Concomitant medications will be coded using the World Health Organization (WHO) Drug Dictionary drug codes. Concomitant medications will be further coded to the appropriate Anatomical Therapeutic Chemical (ATC) code indicating therapeutic classification. Prior and concomitant medications will be summarized and listed by drug and drug class in the clinical study report for this trial. Prior medications will be defined as medications that stopped before the first dose of study drug. Concomitant medications will be defined as medications that 1)

started before the first dose of study drug and were continuing at the time of the first dose of study drug, or 2) started on or after the date of the first dose of study drug up to 30 days after the patient's last dose. A listing of prior and concomitant medications will be included in the clinical study report (CSR) for this trial.

9.5. Efficacy Analyses

The primary endpoint is OS. The tests of OS and the secondary endpoints including the key secondary endpoint of ORR will be carried out sequentially. The order of testing for secondary efficacy endpoints will follow the order in which they are presented in Section 2.2.2.

No multiplicity adjustments will be needed for testing both noninferiority and superiority on OS due to closed testing principle. Treatment arm comparison for ORR and the secondary endpoints will be performed if noninferiority for the primary efficacy endpoint, OS, is declared. The testing will continue until the first nonsignificant outcome occurs. All tests will be performed at one-sided $\alpha = 0.025$ (or 2-sided $\alpha = 0.05$), unless otherwise specified.

9.5.1. Primary Efficacy Analyses

9.5.1.1. Overall Survival

The OS will be compared between tislelizumab (Arm A) and sorafenib (Arm B) via testing the null hypothesis of noninferiority:

H_0: $HR_{A/B} \geq 1.08$ against the alternative hypothesis of noninferiority:

H_a: $HR_{A/B} < 1.08$, where $HR_{A/B}$ denotes the true HR for tislelizumab versus sorafenib. A 1-sided type 1 error of 0.025 will be used in test of OS.

The 95% CI of $HR_{A/B}$ will be estimated in the ITT analysis set using a Cox proportional hazard model with treatment arm as a factor and stratified by the actual value of the pooled stratification factors at randomization (eg, region [Asia vs US/EU], etiology, macrovascular invasion and/or extrahepatic spread [present vs absent] and ECOG). Noninferiority will be declared if the upper limit of the 95% CI for $HR_{A/B}$ is < 1.08.

In the final analysis, superiority of tislelizumab over sorafenib will be tested for OS using a stratified log-rank test in the ITT analysis set only when noninferiority is demonstrated. Superiority will be declared if the one-sided p-value crosses the boundary specified in Table 8 in favor of Arm A in the stratified log-rank test at the final analysis.

The median OS and the cumulative probability of OS estimated at every 6 months will be calculated using Kaplan-Meier estimates for each treatment arm and presented with 2-sided 95% CIs.

<u>Noninferiority of Overall Survival Margin Justification:</u>

Data from 2 sorafenib trials (SHARP and Asia-Pacific) are used to derive the noninferiority margin. In the SHARP trial, the estimated HR (sorafenib/placebo) is 0.69 with a 95% CI of (0.55, 0.87). In the Asia-Pacific trial, the estimated HR (sorafenib/placebo) is 0.68 with a 95% CI of (0.50, 0.93). The pooled HR and its 95% CI is estimated as 0.6865 (0.5709, 0.8255) according to meta-analysis method (Parmar 1998). Using the 95% CI lower limit method on log HR

(Rothmann 2003), the noninferiority margin corresponding to 60% retention of sorafenib effect over placebo is calculated as 1.08.

Timing and Stopping Boundary in the Interim and Final Analyses of Overall Survival:

There will be 1 interim analysis of OS superiority. The interim analysis will be performed when approximately 403 deaths (80% of the expected number of 504 deaths) between the 2 treatment arms are observed. It is estimated that it will take approximately 33.9 months to observe 403 deaths.

The final analysis of OS will take place after approximately 504 deaths have been observed. The upper (efficacy) boundary is based on the O'Brien-Fleming boundary approximated by the Hwang-Shih-DeCani spending function by setting gamma parameter at -4.

Stopping boundaries (p-value and Z score) of superiority test for OS at the interim and final analyses are shown in Table 8. The boundaries will be updated from the actual death events observed at the time of the interim analysis and final planned number of deaths, using the alpha-spending function.

Table 8: Stopping Boundaries (p-value and Z score) and Approximate Hazard Ratio Threshold for Interim and Final Analyses of Superiority Test for Overall Survival

	Time (Months)	Number of Deaths	p-value (Z score) for Efficacy	Approximate Hazard Ratio for Efficacy
**Interim analysis**	33.9	403	< 0.0110 (> 2.29)	< 0.795
**Final analysis**	46.6	504	< 0.0221 (> 2.01)	< 0.8358

Subgroup Analyses:

To determine if the treatment effect is consistent across various subgroups, the HR estimates of OS and its 95% CI will be estimated and plotted within each category of the following variables: geography (Asia [excluding Japan] vs Japan vs Rest of World), macrovascular invasion (present or absent), extrahepatic spread (present or absent), age (≤ 65 vs > 65 years), gender (Female vs Male), ECOG PS (0 vs 1), hepatitis virus infection (HCV vs others [includes HBV]), and BCLC Stage (Stage B vs Stage C).

Country specific subgroups may also be summarized per local regulatory requirements. Other variables may be explored in subgroup analyses as needed.

9.5.2. Secondary Efficacy Analyses

9.5.2.1. Objective Response Rate:

The ORR by BIRC per RECIST v1.1 will be compared between tislelizumab and sorafenib via testing the null hypothesis:

H_0: ORR in Arm A = ORR in Arm B against the alternative:

H_a: ORR in Arm A > ORR in Arm B

The statistical significance of the difference in ORR between arms in the ITT analysis set will be evaluated using the Cochran-Mantel-Haenszel chi-square test with the actual value of the

stratification factors as strata, tested at an alpha level of 0.05 (2-sided). The 2-sided 95% CIs for the odds ratio and the difference in ORR will be calculated, as well as Clopper-Pearson 95% CIs for the ORR within each arm

9.5.2.2. Progression-Free Survival

Progression-free survival will be compared between the 2 arms in the ITT analysis set using a stratified log-rank test with actual value of the stratification factors as strata.

The median PFS and the cumulative probability of PFS at every 3 months will be calculated for each treatment arm and presented with 2 sided 95% CIs. PFS will be estimated using the Kaplan-Meier method. PFS censoring rule will follow the FDA Guidance for Industry *Clinical Trial Endpoints for the Approval of Cancer Drugs and Biologics* (2007) (FDA Guidance for Industry 2007).

The actual tumor assessment visit date will be used to calculate PFS. Data for patients without disease progression or death at the time of analysis will be censored at the time of the last valid tumor assessment. Data for patients who start to receive new anticancer therapy or are lost to follow-up will be censored at the last valid tumor assessment date prior to the introduction of new therapy or lost to follow-up. Patients who have a clinical determination of progression should undergo a CT/MRI, if possible, to correlate radiographic findings with the clinical findings. If a clinical determination of progression for a patient is confirmed, the date of the CT/MRI scan will be considered as the progression date for that patient. More details will be given in the SAP.

9.5.2.3. Duration of Response and Time to Progression

The DOR and TTP by BIRC per RECIST v1.1 will be analyzed similarly to PFS. The DOR will be summarized within responders.

9.5.2.4. Health-Related Quality of Life

The EORTC QLQ-HCC18 postbaseline index scores (Li 2017) will be compared between the 2 treatment arms using a mixed model with baseline score and time since the randomization as covariates. Significant interaction between treatment and time since randomization or quadratic term of time since randomization (p-value < 0.05) will also be included in the final model.

A sensitivity analysis of time to deterioration of EORTC QLQ-HCC18 index-score will be performed. Time to deterioration will be defined as time from randomization to a worsening of HCC 18 index-score from baseline according to minimum clinically meaningful difference which will be specified in the SAP prior to analysis. A stratified log-rank test, similar to that described above, will be used to compare the difference between the 2 treatment arms.

The EORTC QLQ C30 index-score will be analyzed similarly.

For each of the EORTC QLQ HCC18 and EORTC QLQ C30 domains or single items and EQ-5D-5L scales, summary statistics at each assessment time point and its change from baseline will be presented.

9.5.2.5. Secondary Endpoints Assessed by the Investigator

The ORR, PFS, DOR, and TTP assessed by investigator will be analyzed similarly to the approach used in assessment by BIRC.

The BOR is defined as the best response recorded from randomization until data cut or the start of new anticancer treatment. Patients with no postbaseline response assessment (due to any reason) will be considered non-responders for BOR. The proportion and its corresponding Clopper-Pearson 95% CI for each of the response categories (CR, PR, SD, and PD) will be presented by treatment arm.

Both DCR and CBR will be analyzed similarly to ORR in the ITT analysis set.

9.6. Safety Analyses

Safety will be assessed by monitoring and recording of all AEs graded by NCI-CTCAE v4.03. Laboratory values (eg, hematology, clinical chemistry), vital signs, ECGs, and physical examinations will also be used in determining safety. Descriptive statistics will be used to analyze all safety data in the Safety analysis set.

9.6.1. Extent of Exposure

Extent of exposure to each study drug will be summarized descriptively as the number of cycles received (number and percentage of patients), duration of exposure (days), cumulative total dose received per patient (mg), dose intensity, and relative dose intensity.

The number (percentage) of patients requiring dose reduction, interruption, dose delay, and drug discontinuation due to AEs will be summarized for each study drug. Frequency of the above dose adjustments and discontinuation will be summarized by category.

Patient data listings will be provided for all dosing records and for calculated summary statistics.

9.6.2. Adverse Events

The AE verbatim descriptions (investigator's description from the eCRF) will be classified into standardized medical terminology using Medical Dictionary for Regulatory Activities (MedDRA). The AEs will be coded to MedDRA (Version [v]20.0 or higher) lower level term closest to the verbatim term. The linked MedDRA System Organ Class and Preferred Term are also classified.

In this protocol, a TEAE is defined as any AE or SAE with either an onset date or a date of worsening in severity from baseline (ie, pretreatment) occurring on or after first dose of study drug and up to either 30 days following discontinuation from study drug or start of new anticancer therapy, whichever occurs first. The TEAE classification also applies to irAEs and related SAEs that are recorded up to 90 days after discontinuation from study drug, regardless of whether or not the patient starts a new anticancer therapy. Only those AEs that were treatment-emergent will be included in summary tables. All AEs, treatment-emergent or otherwise, will be presented in patient data listings.

The incidence of TEAEs will be reported as the number (percentage) of patients with TEAEs by system organ class (SOC) and Preferred Term. A patient will be counted only once by the highest severity grade per NCI-CTCAE v4.03 within an SOC and Preferred Term, even if the

patient experienced more than 1 TEAE within a specific SOC and Preferred Term. The number (percentage) of patients with TEAEs will also be summarized by relationship to the study drug. Treatment-related AEs include those events considered by the investigator to be related to study treatment or with missing assessment of the causal relationship. The SAEs, deaths, TEAEs with ≥ Grade 3 severity, irAE, treatment-related TEAEs, and TEAEs that led to treatment discontinuation, dose interruption, dose reduction, or dose delay will be summarized.

9.6.3. Laboratory Analyses

Clinical laboratory (eg, hematology, serum chemistry) values will be evaluated for each laboratory parameter as appropriate. Abnormal laboratory values will be flagged and identified as those outside (above or below) the normal range. Reference (normal) ranges for laboratory parameters will be included in the CSR for this trial. Descriptive summary statistics (eg, n, mean, standard deviation, median, minimum, maximum for continuous variables; n [%] for categorical variables) for laboratory parameters and their changes from baseline will be calculated. Laboratory values will be summarized by visit and by worst postbaseline visit.

Laboratory parameters that are graded in NCI-CTCAE v4.03 will be summarized by NCI-CTCAE grade. In the summary of laboratory parameters by NCI-CTCAE grade, parameters with NCI-CTCAE grading in both high and low directions (eg, glucose, potassium, sodium) will be summarized separately.

9.6.4. Vital Signs Analyses

Descriptive statistics for vital sign parameters (systolic and diastolic blood pressure, heart rate, temperature, and weight) and changes from baseline will be presented by visit. Vital signs will be listed by patient and visit.

9.6.5. Ophthalmologic Examination

Ophthalmologic examination results will be listed by patient.

9.7. Pharmacokinetic Analyses

Pharmacokinetic samples will be collected in this study as outlined in Appendix 1, and only from patients randomized to receive tislelizumab in sites that are able to adequately perform PK sampling, handling, and processing procedures as outlined in the Laboratory Manual.

Tislelizumab postdose and trough serum concentration (C_{trough}) data will be tabulated and summarized by visit/cycle at which these concentrations are collected. Descriptive statistics will include means, medians, ranges, and standard deviations, as appropriate.

Additional PK analyses, including population PK analyses and exposure-response (efficacy, safety endpoints) analyses may be conducted as appropriate and the results of such analysis may be reported separately from the CSR.

9.8. Immunogenicity Analyses

Samples to assess anti-tislelizumab antibodies will be collected only in patients randomized to receive tislelizumab and in sites that are able to adequately perform sampling, handling and processing procedures outlined in the Laboratory Manual.

The immunogenicity results will be summarized using descriptive statistics by the number and percentage of patients who develop detectable ADAs. The incidences of positive ADAs and neutralizing ADAs will be reported for evaluable patients. The effect of immunogenicity on PK, efficacy, and safety may be evaluated if data allow.

9.9. Other Exploratory Analyses

Distribution of PD-L1 expression will be examined in the ITT analysis set. Potential association between PD-L1 expression and tislelizumab treatment effect over sorafenib will be explored. Other potential predictive markers will be assessed.

Methodology for exploratory analyses will be described in the SAP.

9.10. Determination of Sample Size

The sample size calculation is based on the number of events required to demonstrate the noninferiority and superiority of tislelizumab arm (Arm A) to sorafenib arm (Arm B) in the comparison of OS.

Under the original OS HR assumption of 0.75 (13.3 months in Arm A versus 10.0 months in Arm B), approximately 504 deaths in total were planned to have approximately 89% power in the superiority test and 98.4% power in the noninferiority test with a 1.08 noninferiority margin. A total of 640 patients were to be randomized in a 1:1 ratio to Arms A and B over a 16-month period at a constant enrollment rate (40 patients/month). An interim analysis was planned after 75% of the targeted number of OS events (ie, approximately 378 deaths) occurred.

After reviewing recent data, the interim analysis was postponed until approximately 80% of the targeted number of OS events (ie, 403 deaths) were observed, with the planned number of deaths in the final analysis remaining at 504. At the time of protocol amendment 5.0, enrollment was completed with a total of 674 patients randomized. Using a more conservative HR assumption of 0.79 at the time of final analysis after an initial 7-month delayed treatment effect (ie, assuming HR = 1 in the first 7 months), the estimated powers for the superiority test are 44% in the interim analysis and 72% in the final analysis using a simulation. The targeted number of events are estimated to occur approximately 33.9 and 46.6 months after study initiation under the actual enrollment rates, updated HR assumptions, and a median OS of 13.5 months in Arm B. The power in the noninferiority test is 0.935 under the updated assumptions.

In the comparison of key secondary endpoint, ORR, the power of a Miettinen and test Nurminen (Miettinen and Nurminen 1985) comparing 2 binomial rates for superiority in 674 patients is approximately 99%, assuming an ORR of 0.015 and 0.05 in Arms A and B, respectively.

9.11. Interim Analyses

An interim analysis for OS superiority test (see Section 9.5.1.1) will be performed by an independent statistician external to the sponsor. The independent statistician will work with the blinded study statistician to provide statistical outputs to the IDMC as described in the IDMC Charter and perform any ad hoc analyses requested by the IDMC.

The aim of this interim analysis is to determine if there is convincing evidence of outstanding OS benefit or futility that the study is unlikely to demonstrate OS benefit. An IDMC will be

responsible for making the recommendation regarding stopping the study early based on predefined criteria for OS, as well as results from ORR and other secondary efficacy endpoints. More details will be given in the IDMC Charter. Patients will continue to be randomized if enrollment has not been completed and treated per-protocol at the time of interim analysis and until a final decision is made by the sponsor after considering the recommendation provided by the IDMC.

10. STUDY COMMITTEES AND COMMUNICATION

10.1. Blinded Independent Review Committee

A BIRC will be established to perform an independent review of all radiological images for the efficacy analysis, and to determine all instances of response and disease progression on the basis of the RECIST v1.1 criteria, in addition to the local investigator’s review of radiographs. The results from the investigator’s review of radiographic images will be used to determine whether patients should be enrolled or should continue on-study treatment. The tumor assessment by the BIRC will be used for the reporting of the study results.

All decisions made during the performance of the study will be based on the local investigator’s assessment of radiographic images, clinical status, and relevant examination of the patients. Sites will submit specific radiographic image files to the centralized data review facility during the study on an ongoing basis or at the sponsor’s request. Detailed rules and guidelines for radiographic imaging and tumor assessments by the BIRC are outlined separately in the Imaging Manual and BIRC Charter.

10.2. Independent Data Monitoring Committee

Regular safety monitoring (at least every 6 months) and efficacy monitoring will be performed by an IDMC. The IDMC may recommend modifications to the study including termination of the study due to safety and/or efficacy concerns. The function and membership of the IDMC will be described in the IDMC Charter.

In addition to the planned IDMC review(s), ad hoc reviews may take place based on new information.

Following IDMC review and discussion, the sponsor will make all final decisions regarding any change in study conduct. Please see the details in the IDMC Charter.

11. SOURCE DOCUMENTS AND ACCESS TO SOURCE DATA/DOCUMENTS

The investigator must maintain adequate and accurate records to ensure that the conduct of the study may be fully documented. Such records include, but are not limited to, the protocol, protocol amendments, ICFs, and documentation of IRB/IEC and governmental approvals. In addition, at the end of the study, the investigator will receive patient data, which will include an audit trail containing a complete record of all changes to such data.

11.1. Access to Information for Monitoring

In accordance with International Council for Harmonisation (ICH) GCP guidelines, the study monitor must have direct access to the investigator's source documentation to verify the data recorded in the eCRFs for consistency.

The monitor is responsible for routine review of the eCRFs at regular intervals throughout the study to verify adherence to the protocol and the completeness, consistency, and accuracy of the data being entered on them. The monitor should have access to any patient records needed to verify the entries on the eCRFs. The investigator agrees to cooperate with the monitor to ensure that any problems detected during these monitoring visits are resolved.

11.2. Access to Information for Auditing or Inspections

Representatives of regulatory authorities or of BeiGene may conduct inspections or audits any time during or after completion of this clinical study. If the investigator is notified of an inspection by a regulatory authority the investigator agrees to notify the sponsor or its designee immediately. The investigator agrees to provide to representatives of a regulatory agency or BeiGene access to records, facilities, and personnel for the effective conduct of any inspection or audit.

12. QUALITY ASSURANCE AND QUALITY CONTROL

12.1. Regulatory Authority Approval

The sponsor will obtain approval to conduct the study from the appropriate regulatory agency in accordance with any applicable country-specific regulatory requirements or file the protocol to the appropriate regulatory agency before the study is initiated at a study center in that country.

12.2. Quality Assurance

To ensure compliance with GCP and all applicable regulatory requirements, the sponsor may conduct a quality assurance audit. Regulatory agencies may also conduct a regulatory inspection of this study. Such audits/inspections can occur at any time during or after completion of the study. If an audit or inspection occurs, the investigator and institution agree to allow the auditor/inspector direct access to all relevant documents and to allocate his/her time and the time of his/her personnel to the auditor/inspector to discuss findings and any relevant issues.

12.3. Study Site Inspections

This study will be organized, performed, and reported in compliance with the protocol, standard operating procedures, working practice documents, and applicable regulations and guidelines. Site audits may be performed periodically by the sponsor's or the contract research organization's qualified compliance auditing team, which is an independent function from the study team responsible for conduct of the study.

Site visits will be conducted by the sponsor or an authorized representative to inspect study data, patients' medical records, and eCRFs. The investigator is to permit national and local health authorities; sponsor study monitors, representatives, and collaborators; and IRB/IEC members to inspect all facilities and records relevant to this study.

12.4. Drug Accountability

The investigator or designee (ie, pharmacist) is responsible for ensuring adequate accountability of all used and unused study drug. This includes acknowledgment of receipt of each shipment of study product (quantity and condition), patient drug dispensation records and returned or destroyed study product. Dispensation records will document quantities received from BeiGene's designated depot or its designee and quantities dispensed to patients, including lot number, date dispensed, patient identifier number, patient initials, and the initials of the person dispensing the medication.

At study initiation, the monitor will evaluate the site's standard operating procedure for study drug disposal/destruction to ensure that it complies with BeiGene's requirements specified in the Pharmacy Manual. At appropriate times during the conduct of the study or at the end of the study, following final drug inventory reconciliation by the monitor and after receiving written sponsor approval, the study site will dispose of and/or destroy all unused study drug supplies, including empty containers, according to these procedures. If the site cannot meet BeiGene's requirements specified in the Pharmacy Manual for disposal, arrangements will be made between the site and BeiGene or its representative for destruction or return of unused study drug supplies.

All drug supplies and associated documentation will be periodically reviewed and verified by the study monitor over the course of the study.

13. ETHICS/PROTECTION OF HUMAN SUBJECTS

13.1. Ethical Standard

This study will be conducted by the principal investigator and the study center in full conformance with the ICH E6 guideline for GCP and the principles of the Declaration of Helsinki or the laws and regulations of the country in which the research is conducted, whichever affords the greater protection to the individual. The study will comply with the requirements of the ICH E2A guideline (Clinical Safety Data Management: Definitions and Standards for Expedited Reporting).

13.2. Institutional Review Board/Independent Ethics Committee

This protocol, the ICFs, any information to be given to the patient, and relevant supporting information must be submitted to the IRB/IEC by the principal investigator and reviewed and approved by the IRB/IEC before the study is initiated. In addition, any patient recruitment materials must be approved by the IRB/IEC. Copies of the IRB/IEC correspondence and approval of the amended ICF/other information and the approved amended ICF/other information must be forwarded to the sponsor promptly.

The principal investigator is responsible for providing written summaries of the status of the study to the IRB/IEC annually or more frequently in accordance with the requirements, policies, and procedures established by the IRB/IEC. Investigators are also responsible for promptly informing the IRB/IEC of any protocol amendments. In addition to the requirements for reporting all AEs to the sponsor, investigators must comply with requirements for reporting SAEs to the local health authority and IRB/IEC. Investigators may receive written investigational new drug (IND) Safety Reports or other safety-related communications from the sponsor. Investigators are responsible for ensuring that such reports are reviewed and processed in accordance with health authority requirements and the policies and procedures established by their IRB/IEC and archived in the site’s study file.

13.2.1. Protocol Amendments

Any protocol amendments will be prepared by the sponsor. All protocol modifications must be submitted to competent authorities according to local requirements and to the IRB or IEC together with, if applicable, a revised model ICF in accordance with local requirements. Written documentation from competent authorities (according to local requirements) and from the IRB or IEC and required site approval must be obtained by the sponsor before changes can be implemented, except for changes necessary to eliminate an immediate hazard to patients or changes that involve logistical or administrative aspects only (eg, change in sponsor medical monitor or contact information).

Information on any change in risk and /or change in scope must be provided to patients already actively participating in the study, and they must read, understand and sign each revised ICF confirming their willingness to remain in the trial.

13.3. Informed Consent

The sponsor's sample ICF will be provided to each site. If applicable, it will be provided in a certified translation of the local language. The final IRB/IEC-approved ICFs must be provided to the sponsor for health authority submission purposes according to local requirements.

The ICFs must be signed and dated by the patient or the patient's legally authorized representative before his or her participation in the study. The case history or clinical records for each patient shall document the informed consent process and that written informed consent was obtained prior to participation in the study.

The ICFs will be revised whenever there are changes to study procedures or when new information becomes available that may affect the willingness of the patient to participate. The final revised IRB-/IEC-approved Consent Forms must be provided to the sponsor for health authority submission purposes.

Patients must be re-consented to the most current version of the ICFs (or to a significant new information/findings addendum in accordance with applicable laws and IRB/IEC policy) during their participation in the study. For any updated or revised ICFs, the case history or clinical records for each patient shall document the informed consent process and that written informed consent was obtained using the updated/revised ICFs for continued participation in the study.

A copy of each signed ICF must be provided to the patient or the patient's legally authorized representative. All signed and dated ICFs must remain in each patient's study file or in the site file and must be available for verification by study monitors at any time.

13.4. Patient and Data Confidentiality

The principal investigator and sponsor will maintain confidentiality and privacy standards by following applicable data privacy laws covering the collection, storage, transmission, and processing of patients' personal and medical information. The principal investigator will maintain confidentiality standards by coding each patient enrolled in the study through assignment of a unique patient identification number. This approach ensures that patients' names are not included in any data set transmitted to any sponsor location.

Patient medical information obtained during this study is confidential and may be disclosed only to third parties as permitted by the signed ICF (or a separate authorization for the use and disclosure of personal health information that has been signed by the patient), unless permitted or required by law. In the event of a breach of the confidentiality of a patient's personal and medical information, the principal investigator and sponsor, as appropriate, shall fulfill all remediation steps and reporting obligations under applicable data privacy laws.

Medical information may be given to a patient's personal physician or other appropriate medical personnel responsible for the patient's welfare, for treatment purposes.

Data generated during this study must be available for inspection upon request by representatives of the US Food and Drug Administration (FDA), the China FDA, and all other national and local health authorities; by sponsor monitors, representatives, and collaborators; and by the IRBs/IECs for each study site, as appropriate.

The investigator must assure that patients' anonymity will be strictly maintained and that their identities are protected from unauthorized parties. The investigator agrees that all information received from the sponsor, including but not limited to the Investigator's Brochure, this protocol, eCRFs, the IND, and any other study information, remain the sole and exclusive property of the sponsor during the conduct of the study and thereafter. This information is not to be disclosed to any third party (except employees or agents directly involved in the conduct of the study or as required by law) without prior written consent from the sponsor. The investigator further agrees to take all reasonable precautions to prevent the disclosure by any employee or agent of the study site to any third party or otherwise into the public domain.

If a written contract for the conduct of the study is executed, and that contract includes confidentiality provisions inconsistent with this section, that contract's provisions shall apply to the extent they are inconsistent with this section.

14. FINANCIAL DISCLOSURE

Investigators are required to provide the sponsor with sufficient accurate financial information in accordance with regulations to allow the sponsor to submit complete disclosure or certification to the absence of certain financial interest of the clinical investigators and/or disclose those financial interests, as required to the appropriate health authorities. This is intended to ensure financial interests and arrangements of the clinical investigators with BeiGene or Celgene that could affect reliability of data submitted to health authorities are identified and disclosed by the sponsor. Investigators are responsible for providing information about their financial interests before participation in the study, and to update this information if any relevant changes occur during the course of the study and for 1 year after completion of the study (ie, last patient, last visit).

15. DATA HANDLING AND RECORD KEEPING

15.1. Data Collection and Management Responsibilities

15.1.1. Data Collection

Data required by the protocol will be entered into the eCRFs in an electronic data capture (EDC) system that is compliant with all regulatory requirements. All study-related data collected or received by the investigator or study team shall be promptly entered into the eCRFs. In no event should the entry of the study data into the eCRF be later than what is stipulated in the site contract after the data is collected or received by the investigator or study team without prior communication with and approval by sponsor.

Data collection in the eCRF should follow the instructions described in the eCRF Completion Guidelines. The investigator has ultimate responsibility for the collection and reporting of all clinical data entered in the eCRF. The investigator or designee as identified on Form FDA 1572 must provide e-signature in the EDC system to attest to its accuracy, authenticity, and completeness.

Data contained in the eCRFs are the sole property of BeiGene and should not be made available in any form to third parties without written permission from BeiGene, except for authorized representatives of BeiGene or appropriate regulatory authorities.

15.1.2. Data Management/Coding

All final patient data, both eCRF and external data (eg, laboratory data), collected according to the protocol, will be stored by BeiGene at the end of the study.

Standard procedures (including following data review guidelines, computerized validation to produce queries and maintenance of an audit file that includes all database modifications) will be followed to support accurate data collection. Data will be reviewed for outliers, logic, data inconsistencies and completeness.

During the study, a study monitor (clinical research associate) will make site visits to review protocol compliance, compare eCRFs against individual patient's medical records and ensure that the study is being conducted according to pertinent regulatory requirements.

The eCRF entries will be verified with source documentation. The review of medical records will be performed in a manner to ensure that patient confidentiality is maintained. Checking the eCRFs for completeness, clarity and cross checking with source documents is required to monitor the progress of the study. Direct access to source data is also required for inspections and audits and will be carried out with due consideration given to data protection and medical confidentiality.

Adverse events will be coded using the MedDRA Version 20.0 or higher. Concomitant medications will be coded using the WHO Drug Dictionary. Concomitant diseases/medical history will be coded using the MedDRA Version 20.0 or higher.

15.2. Data Integrity

Due to the open-label design of the study, access to the patient level clinical data in the EDC and IRT systems will be assigned to predefined study personnel only. Functions/persons with access to the EDC system shall be prohibited from using the EDC system to generate unnecessary listings/summaries that may introduce unwanted bias, or share such outputs or the unblinded data from the EDC system with other functions/persons who do not have access to the EDC. Although the trial is open-label, analyses or summaries generated by randomized treatment assignment and actual treatment received will be limited and documented.

15.3. Study Records Retention

The investigator must maintain adequate and accurate records to enable the conduct of the study to be fully documented and the study data to be subsequently verified. These documents should be classified into at least 1 of the following categories: 1) investigator’s study file, and/or 2) patient clinical source documents.

The investigator’s study file will contain the protocol/amendments, eCRF and query forms, IRB/IEC, and governmental approval with correspondence, informed consent, drug records, staff curriculum vitae and authorization forms, and other appropriate documents and correspondence.

Patient clinical source documents (usually defined by the project in advance to record key efficacy/safety parameters independent of the eCRFs) would include documents such as (although not be limited to) the following: patient hospital/clinic records, physician’s and nurse’s notes, appointment book, original laboratory reports, ECG, electroencephalogram, X-ray, pathology and special assessment reports, consultant letters, Screening and enrollment log, etc.

Following closure of the study, the investigator must maintain all study records in a safe and secure location. The records must be maintained to allow easy and timely retrieval, when needed (eg, audit or inspection), and, whenever feasible, to allow any subsequent review of data in conjunction with assessment of the facility, supporting systems, and personnel. Where permitted by local laws/regulations or institutional policy, some or all of these records can be maintained in a format other than hard copy (eg, microfiche, scanned, electronic); however, caution needs to be exercised before such action is taken. The investigator must assure that all reproductions are legible, are a true and accurate copy of the original, and meet accessibility and retrieval standards, including re-generating a hard copy, if required. Furthermore, the investigator must ensure there is an acceptable back up of these reproductions and that an acceptable quality control process exists for making these reproductions.

The sponsor will inform the investigator of the time period for retaining these records to comply with all applicable regulatory requirements. The minimum retention time will meet the strictest standard applicable to that study center for the study, as dictated by any institutional requirements or local laws or regulations, or the sponsor’s standards/procedures; otherwise, the retention period will default to the 15 years.

The investigator must notify the sponsor of any changes in the archival arrangements, including, but not limited to, the following: archival at an off-site facility, or transfer of ownership of or responsibility for the records in the event the investigator leaves the study center.

If the investigator cannot guarantee this archiving requirement at the study site for any or all of the documents, special arrangements must be made between the investigator and BeiGene to store these in sealed containers outside of the site so that they can be returned sealed to the investigator in case of a regulatory audit. When source documents are required for the continued care of the patient, appropriate copies should be made for storage outside of the site.

Biological samples at the conclusion of this study may be retained as outlined in the agreement with the contract research organization managing the biological samples, for the shorter of: a period of up to 10 years or as allowed by your IRB/IEC.

16. PROTOCOL DEVIATIONS

The investigator is responsible for ensuring that the study is conducted in accordance with the procedures and evaluations described in this protocol. Investigators assert they will apply due diligence to avoid protocol deviations.

The investigator is to document and explain any deviations from the IRB/IEC approved protocol. The investigator must promptly report any major deviations that might impact patient safety and/or data integrity to the sponsor and to the IRB/IEC, in accordance with established IRB/IEC policies and procedures, and shall report all protocol deviations to the sponsor.

17. PUBLICATION AND DATA SHARING POLICY

A clinical study report will be prepared and provided to the regulatory agency(ies). BeiGene will ensure that the report meets the standards set out in the ICH Guideline for Structure and Content of Clinical Study Reports (ICH E3). Note that an abbreviated report may be prepared in certain cases.

The results of this study will be published or presented at scientific meetings in a timely, objective, and clinically meaningful manner that is consistent with good science, industry and regulatory guidance, and the need to protect the intellectual property of BeiGene (sponsor), regardless of the outcome of the trial. The data generated in this clinical trial are the exclusive property of the sponsor and are confidential. For multicenter studies, the first publication or disclosure of study results shall be a complete, joint multicenter publication or disclosure coordinated by the sponsor. Thereafter, any secondary publications will reference the original publication(s). Authorship will be determined by mutual agreement and all authors must meet the criteria for authorship established by the International Committee of Medical Journal Editors or stricter local criteria (International Committee of Medical Journal Editors).

After conclusion of the study and without prior written approval from BeiGene, investigators in this study may communicate, orally present, or publish in scientific journals or other scholarly media only after the following conditions have been met:

- The results of the study in their entirety have been publicly disclosed by or with the consent of BeiGene in an abstract, manuscript, or presentation form; or
- The study has been completed at all study sites for at least 2 years.
- No such communication, presentation, or publication will include BeiGene's confidential information.
- Each investigator agrees to submit all manuscripts or congress abstracts and posters/presentations to the sponsor prior to submission. This allows the sponsors to protect proprietary information, provide comments based on information from other studies that may not yet be available to the investigator, and ensure scientific and clinical accuracy. The details of the processes of producing and reviewing reports, manuscripts, and presentations based on the data from this trial will be presented in the investigator's clinical study agreement.

17.1. Study and Study Center Closure

Upon completion of the study, the monitor will conduct the following activities in conjunction with the investigator or study center personnel, as appropriate:

- Return of all study data to the sponsor
- Resolution and closure of all data queries
- Accountability, reconciliation, and arrangements for unused study drug(s)
- Review of study records for completeness
- Return of treatment codes to the sponsor

- Shipment of PK and ADA samples to assay laboratories

In addition, the sponsor reserves the right to suspend the enrollment or prematurely discontinue this study either at a single study center or at all study centers at any time for reasons including, but not limited to, safety or ethical issues or severe noncompliance. If the sponsor determines such action is needed, the sponsor will discuss this with the investigator (including the reasons for taking such action) at that time. When feasible, the sponsor will provide advance notification to the investigator of the impending action prior to it taking effect.

The sponsor will promptly inform all other investigators and/or institutions conducting the study if the study is suspended or terminated for safety reasons, and will also inform the regulatory authorities of the suspension or termination of the study and the reason(s) for the action. If required by applicable regulations, the investigator must inform the IEC/IRB promptly and provide the reason for the suspension or termination.

If the study is prematurely discontinued, all study data must be returned to the sponsor. In addition, arrangements will be made for the return or disposal of all unused study drug(s) in accordance with the applicable sponsor procedures for the study.

Financial compensation to the investigators and/or institutions will be in accordance with the agreement established between the investigator and the sponsor.

17.2. Information Disclosure and Inventions

All rights, title, and interests in any inventions, know-how or other intellectual or industrial property rights which are conceived or reduced to practice by the study center personnel during the course of or as a result of the study are the sole property of the sponsor, and are hereby assigned to the sponsor.

If a written contract for the conduct of the study which includes ownership provisions inconsistent with this statement is executed between the sponsor and the study center, that contract's ownership provisions shall apply rather than this statement.

All information provided by the sponsor and all data and information generated by the study center as part of the study (other than a patient's medical records) are the sole property of the sponsor and will be kept confidential by the investigator and other study center personnel.

This information and data will not be used by the investigator or other study center personnel for any purpose other than conducting the study without the prior written consent of the sponsor.

These restrictions do not apply to:

- Information which becomes publicly available through no fault of the investigator or study center personnel
- Information which is necessary to disclose in confidence to an IEC/IRB solely for the evaluation of the study
- Information which is necessary to disclose to provide appropriate medical care to a patient
- Study results which may be published as described in Section 17.

If a written contract for the conduct of the study which includes provisions inconsistent with this statement is executed, that contract's provisions shall apply rather than this statement.

18. REFERENCES

Abou-Alfa GK, et al. Phase II study of sorafenib in patients with advanced hepatocellular carcinoma. *J Clin Oncol*. 2006;24(26):4293-300.

AASLD/IDSA HCV Guidance Panel. Hepatitis C guidance: AASLD-IDSA recommendations for testing, managing, and treating adults infected with hepatitis C virus. Hepatology. 2015;62: 932–954.

Bai S, et al. A guide to rational dosing of monoclonal antibodies. *Clin Pharmacokinet* 2012;51(2):119-35.

Balogh J, et al., Hepatocellular carcinoma: a review. *J Hepatocell Carcinoma*. 2016; 3: 41–53.

Beers SA, et al. Influence of immunoglobulin isotype on therapeutic antibody function. *Blood*. 2016; 127(9):1097-1101.

BeiGene Investigator's Brochure, Tislelizumab. Edition 5.0, February 2018.

Bersanelli M, Buti S. From targeting the tumor to targeting the immune system: Transversal challenges in oncology with the inhibition of the PD-1/PD-L1 axis. *World J Clin Oncol*. 2017 Feb 10;8(1):37-53.

Bosch FX, et al. Epidemiology of Hepatocellular Carcinoma. *Clin Liver Dis*. 2005 May;9(2):191-211.

Brahmer JR, Lacchetti C, Schneider BJ, et al. Management of immune-related adverse events in patients treated with immune checkpoint inhibitor therapy: American Society of Clinical Oncology Clinical Practice Guideline. J Clin Oncol. 2018. [E-pub ahead of print]

Bruix J, et al. Efficacy and safety of sorafenib in patients with advanced hepatocellular carcinoma: subanalyses of a Phase 12 trial. *J Hepatol*. 2012 Oct; 57(4):821-9.

Cheng AL, et al. Efficacy and safety of sorafenib in patients in the Asia-Pacific region with advanced hepatocellular carcinoma: a Phase 12 randomized, double-blind, placebo controlled trial. *Lancet Oncol*. 2009;10(1):25–34.

Chiba K, et al. A comprehensive review of the pharmacokinetics of approved therapeutic monoclonal antibodies in Japan: Are Japanese phase I studies still needed? *J Clin Pharmacol*. 201454(5):483-94.

Clinical Trials Facilitation Group (CTFG). Recommendations related to contraception and pregnancy testing in clinical trials. September 15, 2014. http://www.hma.eu/fileadmin/dateien/Human_Medicines/01-About_HMA/Working_Groups/CTFG/2014_09_HMA_CTFG_Contraception.pdf.

Choo SP, et al. Comparison of hepatocellular carcinoma in Eastern versus Western populations. *Cancer* 2016;122:3430–3446.

Dahan R, et al. FcgRs Modulate the Antitumor Activity of Antibodies Targeting the PD-1/PD-L1 Axis. *Cancer Cell*. 2015; 28: 285–295.

Dolgin M, Association NYH, Fox AC, Gorlin R, et al. Nomenclature and criteria for diagnosis of diseases of the heart and great vessels. 9th ed. Boston, MA: Lippincott Williams and Wilkins; March 1, 1994

Eisai Co, Ltd. News release, http://www.eisai.com/news/news201706.html, January 25, 2017.

Eisenhauer EA, et al. New response evaluation criteria in solid tumours: revised RECIST guideline (Version 1.1). *Eur J Cancer*. 2009;45:228-247.

El-Khoueiry AB, et al. Nivolumab in patients with advanced hepatocellular carcinoma (CheckMate 040): an open-label, non-comparative, phase 1/2 dose escalation and expansion trial. *Lancet* 2017 Apr 20. pii: S0140-6736(17)31046-2.

El-Serag HB, Rudolph KL. Hepatocellular carcinoma: epidemiology and molecular carcinogenesis. *Gastroenterology*. 2007 Jun;132(7):2557-76.

El-Serag H.B. Epidemiology of viral hepatitis and hepatocellular carcinoma. *Gastroenterology*. 2012 May;142(6):1264-1273.

Finn RS. Treatment of Intermediate-Stage Hepatocellular Carcinoma. *Gastroenterol Hepatol (N Y)*. 2015 Aug;11(8):545-7.

Forner Al, Reig ME, de Lope CR, Bruix J. Current strategy for staging and treatment: the BCLC update and future prospects. *Semin Liver Dis*. 2010 Feb;30(1):61-74.

Gong W, et al. Paclitaxel induced B7-H1 expression in cancer cells via the MAPK pathway. *J Chemother*. 2011;23(5):295–9.

Gul N, van Egmond M. Antibody-Dependent Phagocytosis of Tumor Cells by Macrophages: A Potent Effector Mechanism of Monoclonal Antibody Therapy of Cancer. *Cancer Res*; 2015; 75(23): 5008–13.

Haanen JBAG, et al. Management of Toxicities from Immunotherapy: ESMO Clinical Practice Guidelines for Diagnosis, Treatment and Follow-up. *Annals of Oncology*. 2017; 28 (Supplement 4): iv119–iv142, 2017doi:10.1093/annonc/mdx225

Han K, Kim JH. Transarterial chemoembolization in hepatocellular carcinoma treatment: Barcelona clinic liver cancer staging system. *World J Gastroenterol*. 2015 Sep 28;21(36):10327-35.

Heimbach J, et al. AASLD guidelines for the treatment of hepatocellular carcinoma. Hepatology, 2017 Jan 28. doi: 10.1002/hep.29086. [Epub ahead of print].

Iavarone M, et al. Field-practice study of sorafenib therapy for hepatocellular carcinoma: a prospective multicenter study in Italy. *Hepatology*. 2011;54(6):2055–2063.

International Committee of Medical Journal Editors. Recommendations for the Conduct, Reporting, Editing, and Publication of Scholarly Work in Medical Journals. 2016. Available online: http://www.icmje.org. Accessed 08 August 2017.

Jin Z, Yoon HH. The promise of PD-1 inhibitors in gastro-esophageal cancers: microsatellite instability vs PD-L1. *J Gastrointest Oncol*. 2016;7:771-788.

Keating G.M. Sorafenib: A Review in Hepatocellular Carcinoma. *Target Oncol*. 2017 Apr;12(2):243-253.

Kloth JSL., et al. Incidence and relevance of QTc-interval prolongation caused by tyrosine kinase inhibitors. *Br J Cancer*. 2015 Mar 17; 112(6): 1011–1016.

Labrijn AF, et al. Therapeutic IgG4 antibodies engage in Fab-arm exchange with endogenous human IgG4 in vivo. *Nature Biotechnology*. 2009; 27(8): 767-771

Lee JM, Han, KH. Positioning and indication of sorafenib in the treatment algorithm and real practice setting: Western and eastern approach--Asian perspective. *Oncology*. 2010 Jul; 78 Suppl 1:167-71.

Levey AS, Stevens LA, Schmid CH, et al. A new equation to estimate glomerular filtration rate. Ann Intern Med. 2009;150(9):604-12.

Li L, et al. Prognostic values of EORTC QLQ-C30 and QLQ-HCC18 index-scores in patients with hepatocellular carcinoma – clinical application of health-related quality-of-life data. *BMC Cancer*. 2017; 17:8. Published online 2017 Jan 4. doi: 10.1186/s12885-016-2995-5.

Llovet JM, et al. The Barcelona approach: diagnosis, staging, and treatment of hepatocellular carcinoma. *Liver Transpl*. 2004:10(2 Suppl. 1), S115–S120.

Llovet JM, et al. Sorafenib in advanced hepatocellular carcinoma. *N Engl J Med*. 2008;359(4):378–390.

Lucey MR, et al. Minimal criteria for placement of adults on the liver transplant waiting list: a report of a national conference organized by the American Society of Transplant Physicians and the American Association for the Study of Liver Diseases. *Liver Transpl Surg*. 1997;3(6):628-637.

Makarov-Rusher OV, et al. The yin and yang of evasion and immune activation in HCC. *J Hepatol*. 2015; vol.62:1420-1429.

Marrero JA, et al. Observational registry of sorafenib use in clinical practice across Child-Pugh subgroups: The GIDEON study. *J Hepatol*. 2016 Dec;65(6):1140-1147.

Mazzaferro V, et al. Liver transplantation for the treatment of small hepatocellular carcinomas in patients with cirrhosis. *N Engl J Med*. 1996; 334: 693–699.

McDaniel AS, et al. Expression of PDL1 (B7-H1) before and after neoadjuvant chemotherapy in urothelial carcinoma. *Euro Urol Focus*. 2016;1(3):265–8.

Miettinen O, Nurminen M. Comparative analysis of two rates. *Stat Med*. 1985 Apr-Jun;4(2):213-26.

National Cancer Institute Common Terminology Criteria for Adverse Events, Version 4.03. Cancer Therapy Evaluation Program. June 2010.

National Comprehensive Cancer Network Clinical Practice Guidelines. Subcommittee Hepatobiliary Cancers. Available online: https://www.nccn.org/professionals/physician_gls/pdf/hepatobiliary.pdf Version I. 2017 February 2017.

Oikonomopoulos G, et al. Lenvatinib: a potential breakthrough in advanced hepatocellular carcinoma? *Future Oncol*. 2016 Feb;12(4):465-76.

Ono Pharmaceutical Co, Ltd. Available online: https://www.ono.co.jp/eng/news/pdf/sm_cn161227.pdf. Accessed 01 February 2017.

Parmar MK, et al. Extracting summary statistics to perform meta-analyses of the published literature for survival endpoints. *Stat Med*. 1998 Dec 30;17(24):2815-34.

Patel SP, Kurzrock, R. PD-L1 Expression as a Predictive Biomarker in Cancer Immunotherapy. *Mol Cancer Ther*. 2015;14(4):847–56.

Prieto J, et al. Immunological landscape and immunotherapy of hepatocellular carcinoma. Nat. Rev. *Gastroenterol. Hepatol*. 2015 Dec;12(12):681-700.

Pugh RN, et al. Transection of the oesophagus for bleeding oesophageal varices. *Br J Surg*. 1973;60:646-649.

Rothmann M, et al. Design and analysis of non-inferiority mortality trials in oncology. *Stat Med*. 2003 Jan 30;22(2):239-64.

Soar J, Pumphrey R, Cant A, et al. Emergency treatment of anaphylactic reactions--guidelines for healthcare providers. Resuscitation. 2008;77(2):157-69.

Terrault N A, Bzowej N H, Chang K-M Jonas MM, and Murad MH. AASLD guidelines for treatment of chronic hepatitis B. *Hepatology*. 2016;63: 261–283.

Topalian SL, et al. Safety, activity, and immune correlates of anti−PD-1 antibody in cancer. *N Engl J Med* 2012;366:2443−54.

Torre LA, et al. Global Cancer Statistics, 2012. *CA Cancer J Clin*. 2015 Mar;65(2):87-108.

Trey C, et al. Treatment of hepatic coma by exchange blood transfusion. *N Engl J Med*. 1966;274(9):473-481.

U.S. Food and Drug Administration. Product labels for FDA approved drug products are available online: http://www.accessdata.fda.gov/scripts/cder/daf/index.cfm. Accessed 03 February 2017.

U.S. Food and Drug Administration Center for Drug Evaluation Research (CDER) and Center for Biologics Evaluation and Research. FDA Guidance for Industry: Clinical Trial Endpoints for the Approval of Cancer Drugs and Biologics. 2007.

Van Der Kraak L, et al. 5-Fluorouracil upregulates cell surface B7-H1 (PD-L1) expression in gastrointestinal cancers. *Journal for Immunotherapy of Cancer*. 2016, 4(1):65.

Wang C, et al. In vitro characterization of the anti-PD-1 antibody nivolumab, BMS-936558, and in vivo toxicology in non-human primates. *Cancer Immunol Res* 2014;2:846-56.

Worobec A, Rosenberg AS. A risk-based approach to immunogenicity concerns of therapeutic protein products, Part 1: considering consequences of the immune response to a protein. *BioPharm International*. 2004; Volume 17, Issue 11.

Yamamoto N, et al. Phase I study of Nivolumab, an anti-PD-1 antibody, in patients with malignant solid tumors. Investigational New Drugs. 35.2 (2017): 207–216.

Yamazaki N, et al. Phase 1b Study of Pembrolizumab (MK-3475; Anti-PD-1 Monoclonal Antibody) in Japanese Patients with Advanced Melanoma (KEYNOTE-041). Cancer Chemotherapy and Pharmacology 79.4 (2017): 651-660.

19. APPENDICES

APPENDIX 1. SCHEDULE OF ASSESSMENTS

**Assessment**	**Screening[1]**	**Treatment Cycles**					**Safety Follow-up[3]**	**Survival Follow-up[4]**
		**Cycles 1 to 3 (every 21 days)**			**Cycle 4 and Subsequent Cycles (Every 21 Days)**	**End of Treatment Visit [2]**		
**Days (Window)**	**-28 to ~ -1**	**1 (±3 for C2 and C3)**	**8 (±2)**	**15 (± 2)**	**1 (± 3)**	**0 to 7 Days**	**30 ± 7 Days After Last Dose**	**Every 3 Months (± 14 days)**
Informed consent	x							
Inclusion/exclusion criteria	x							
Randomization		x[5]						
Demographics/medical history/prior medications[6]	x							
Child-Pugh classification score	x[7]							
Vital signs/ height and weight[8]	x	x	x[8]	x[8]	x	x	x	
Physical examination[27]	x	x			x	x	x	
ECOG Performance Status	x	x			x	x	x	
12-lead ECG[9]	x	As clinically indicated					x	
Adverse events[10]	x	x	x[28]	x[28]	x	x	x	x
Concomitant medications	x	x	x[28]	x[28]	x	x	x	
Hematology[11]	x[1]	x	x	x	x	x[2]	x	
Serum chemistry[11]	x[1]	x	x	x	x	x[2]	x	
Coagulation parameters [11, 12]	x	x			x	x[2]	x	
Urinalysis[11]	x	As clinically indicated						

Assessment	Screening[1]	Treatment Cycles					Safety Follow-up[3]	Survival Follow-up[4]
		Cycles 1 to 3 (every 21 days)			Cycle 4 and Subsequent Cycles (Every 21 Days)	End of Treatment Visit [2]		
**Days (Window)**	**-28 to ~ -1**	**1 (±3 for C2 and C3)**	**8 (±2)**	**15 (± 2)**	**1 (± 3)**	**0 to 7 Days**	**30 ± 7 Days After Last Dose**	**Every 3 Months (± 14 days)**
Pregnancy test[13]	x	x			x			
Thyroid function[14]	x[1]				x		x	
Pharmacokinetics[15]		x			x		x	
Pulmonary function tests[16]	x							
Anti-Tislelizumab antibodies[17]		x			x		x	
HBV/HCV tests[18]	x	As clinically indicated						
AFP[11]	x	x			x	x		
Tumor assessment[19]	x				x	x[2]		x
Archival tumor tissue[20]	x							
Fresh tumor tissue (optional) [21]	x							
Tislelizumab administration[22]		x			x			
Sorafenib administration[23]		Daily						
EQ-5D-5L [24]	x	x			x	x		
EORTC QLQ-C30[24]	x	x			x	x		
EORTC QLQ-HCC18[24]	x	x			x	x		
Survival status								x
Optical coherence tomography (or equivalent diagnostic test) and visual acuity tests[25]	x				x	x[26]	x[26]	

1. Written informed consent is required prior to performing any study-specific tests or procedures. Results of standard of care tests or examinations performed prior to obtaining informed consent and within 28 days prior to randomization may be used for Screening assessments rather than repeating such tests.
2. The End of Treatment Visit is conducted when the investigator determines that tislelizumab or sorafenib will no longer be used. If routine laboratory tests (eg, hematology, serum chemistry) are completed within 7 days before the End of Treatment Visit, tests need not be repeated. Tumor assessment is not required at the End of Treatment Visit provided that fewer than 6 weeks have passed since the last assessment.
3. The Safety Follow-up Visit is required to be conducted 30 days (± 7 days) after the last dose of tislelizumab or sorafenib, or before the initiation of a new anticancer treatment, whichever occurs first. In the situation where study treatment is withheld/interrupted for 30 days or more prior to permanent discontinuation of study treatment (End of Treatment Visit), the Safety Follow-up Visit should be conducted at the same time as the End of Treatment Visit. Common procedures to both visits should be performed only once.
4. Survival Follow-up information will be collected via telephone calls, patient medical records, and/or clinic visits approximately every 3 months (± 14 days) after the Safety Follow-up Visit until death, loss to follow-up, withdrawal of consent, or study termination by sponsor. All patients will be followed for survival and subsequent anticancer therapy information unless a patient requests to be withdrawn from follow-up.
5. Patients will be randomized into either the tislelizumab or sorafenib arms via IRT. All patients are required to receive study treatment within 2 days of randomization.
6. Includes age or date of birth, gender, and self-reported race/ethnicity; history of treatment for the primary diagnosis, including prior medication, loco-regional treatment(s), and surgical treatment(s). Information on radiographic studies performed prior to study entry may be collected for review by the investigator.
7. Patients are required to have Child-Pugh A classification for liver function assessed within 7 days before randomization.
8. Vital signs collected on study include temperature, pulse rate, and blood pressure (systolic and diastolic) while the patient is in a seated position after resting for 10 minutes. The patient's vital signs are required to be recorded within 60 minutes before; during; and 30 minutes after the first infusion of tislelizumab. For subsequent infusions, vital signs will be collected within 60 minutes before infusion and if clinically indicated, during and 30 minutes (up to + 15 minutes) after the infusion.
   For patients randomized to the sorafenib arm, monitor blood pressure weekly during the first 6 weeks after the first dose. Thereafter, monitor blood pressure at Day 1 of every cycle. Investigator may perform additional or more frequent blood pressure assessments if clinically indicated.
9. The ECG recordings will be obtained during Screening, the Safety Follow-up Visit, and as clinically indicated at other time points. Patients should be resting for at least 10 minutes prior to each ECG collection. Additional ECG monitoring should be performed following local guidance based on the sorafenib labeling. Abnormal ECGs should be repeated in triplicate.
10. The AEs and laboratory abnormalities will be graded per NCI-CTCAE v4.03. All AEs will also be evaluated for seriousness. After the informed consent form has been signed, but prior to the administration of study drug, only SAEs should be reported. After the first dose of study drug, all AEs and SAEs, regardless of relationship to study drug, will be reported until either 30 days after the last dose of study treatment (including sorafenib) or the initiation of new anticancer therapy, whichever occurs first. Immune-related AEs (serious and nonserious) will be reported for 90 days after the last dose of tislelizumab, regardless of whether or not the patient starts a new anticancer therapy. All drug-related SAEs will be recorded by the investigator after treatment discontinuation until patient death or loss to follow-up, whichever occurs first.
    After a patient has been discontinued from the study drug(s), investigators are not obligated to actively seek AEs or SAEs in former patients. However, if the investigator learns of any SAE, including a death, and he/she considers the SAE related to the study drug, the investigator will notify the sponsor as described in Table 5 in Section 8.7.2.1.
11. Local or central laboratory assessments on serum chemistry, hematology, coagulation, and urinalysis will be conducted, of which certain elements will be collected as specified in Appendix 5. Investigators in regions where central labs are required may also use results from local laboratories for assessing eligibility, safety monitoring and dosing decision. If laboratory tests at screening (hematology, chemistry, coagulation, urinalysis, and alpha fetoprotein) are not performed within 7 days of randomization, these tests should be repeated and reviewed before randomization. Patients with electrolyte deficiencies,

including magnesium, potassium, and calcium, must have these clinically corrected in the screening period before enrollment in the study. Hematology and serum chemistry (including liver function tests) will be performed weekly for the first 3 cycles and then at the beginning of subsequent cycles (data collected as specified in Appendix 5). After Cycle 1, results are to be reviewed within 48 hours before study drug administration. Urinalysis is to be conducted during the treatment period only if clinically warranted. Refer to Section 8.4.5 for additional information regarding clinical assessment and management of clinical laboratory abnormalities.

12. Includes international normalized ratio, prothrombin time, and activated partial thromboplastin time.
13. Urine or serum pregnancy test (for women of childbearing potential, including women who have had a tubal ligation) must be performed and documented as negative within 7 days prior to randomization. If screening pregnancy test is > 7 days prior to randomization, it must be repeated at Cycle 1 Day 1, prior to dosing. Urine pregnancy tests will be performed at each visit prior to dosing. A serum pregnancy test must be performed if the urine pregnancy test is positive or equivocal.
14. Analysis of FT3, FT4, and TSH will be performed by a central laboratory or the local study site laboratory. Thyroid function tests will be performed at Screening and every 3 cycles (ie, Day 1 of Cycles 4, 7, 10, et cetera), and at the Safety Follow-up Visit.
15. Only for patients randomized to tislelizumab treatment. Procedures for collection of PK samples are described in the Laboratory Manual. Predose (within 60 minutes before starting infusion) samples are required to be collected at Day 1 of Cycles 1, 2, 5, 9 and 17; A postdose (within 30 minutes after completing tislelizumab infusion) sample is required to be collected at Day 1 of Cycles 1 and 5. An additional PK sample is required to be collected at the Safety Follow-up. Should a patient present with any ≥ Grade 3 irAE, an additional blood PK sample may be taken to determine the serum concentration of tislelizumab. These tests are required when it is allowed by local regulations/IRBs/ECs.
16. Patients who are suspected or known to have serious/severe respiratory conditions or exhibit significant respiratory symptoms unrelated to the underlying cancer will have pulmonary function testing which may include, but is not limited to, spirometry and assessment of diffusion capacity done during the Screening period to assist the determination of suitability on the study. At least 50% of performance expected by age is required for spirometry (including FVC and FEV_1). Assessment of diffusion capacity during Screening is required in patients with a history of thoracic radiotherapy or restrictive lung disease. When DLCO is performed, at least 60% of performance expected by age is required.
17. Blood used to test for anti-tislelizumab antibodies should be collected within 60 minutes before beginning the Day 1 infusion of Cycles 1, 2, 5, 9, and 17 and at the mandatory Safety Follow-up Visit. All samples should be drawn at the same time as blood collection for predose PK analysis. These tests are required when it is allowed by local regulations/IRBs/ECs.
18. Testing will be performed by a central laboratory and/or the local laboratory at Screening and will include HBV/HCV serology (HBsAg, HBsAb, HBcAb, and HCV antibody) and viral load assessment (HBV DNA and HCV RNA). Patients who have detectable HBV DNA or HCV RNA at Screening will perform the respective viral load test every 4 cycles (ie, Day 1 of Cycle 5, 9, 13, etc).
19. Radiological images captured as standard of care prior to obtaining written informed consent and within 28 days of randomization may be used rather than repeating tests. All measurable and evaluable lesions are required to be assessed and documented at the Screening Visit. An MRI (or CT scan if MRI is contraindicated or not readily available) of the head may be required at screening based on clinical judgement; bone scan or ^{18}F-NaF PET is required if clinically indicated. The same radiographic procedure must be used throughout the study for each patient.
    The investigator must review radiograph results before dosing at the following cycle. Patients will undergo tumor assessments approximately every 9 weeks (± 7 days) during Year 1 and every 12 weeks (± 7 days) from Year 2 onwards (based on RECIST v1.1 assessment). The investigator may perform additional scans or more frequent assessments if clinically indicated. See Section 7.4 for more information. Patients who discontinue study treatment early for reasons other than disease progression (eg, toxicity) will continue to undergo tumor assessments following the original plan until the patient experiences disease progression, withdraws consent, dies, or until the study terminates, whichever occurs first. Patients who continue tislelizumab/sorafenib treatment beyond radiographic disease progression (Section 7.13.1) will be monitored with a follow-up scan no more than 6 to 8 weeks beyond the initial diagnosis of radiographic PD before discontinuation of tislelizumab/sorafenib treatment.

20. Patients are required to provide archival tumor tissues (FFPE blocks or approximately 10 [≥ 5] unstained slides) for biomarker analysis (except if not allowed by local regulations/IRBs/IECs).
21. Fresh biopsy: In the absence of archival tumor tissues, a fresh biopsy of a tumor lesion at baseline is optional (written informed consent is required prior to fresh tumor biopsies). See Section 7.6 for more information.
22. Tislelizumab will be given IV Q3W. The initial infusion (Cycle 1, Day 1) will be delivered over 60 minutes, and then can be administered over 30 minutes for subsequent infusions if well tolerated. Patients must be monitored for 2 hours after infusion of tislelizumab on Day 1 of Cycle 1 and Cycle 2, from Cycle 3 onward, at least a 30-minute monitoring period is required. The first dose will be given on Cycle 1 Day 1 and subsequent dosing will continue on the original 21-day treatment interval schedule. Each cycle has a (±) 3-day window, however in case of dose delays there should be a minimum of 14 days between tislelizumab dosing days.
23. Sorafenib will be self-administered PO at the recommended dose of 400 mg (2 tablets of 200 mg) BID, without food (at least 1 hour before or 2 hours after a meal).
24. To be completed prior to any clinical activities that are performed that day during on-study site visits. EQ5D-5L, EORTC QLQ-C30, and EORTC QLQ-HCC18 will be completed at Screening and/or baseline, at every other cycle through Cycle 12, then every 4 cycles thereafter, and at EOT.
25. Eye exam, visual acuity test, and optical coherence tomography (or equivalent diagnostic test for retinal examination) captured as standard of care prior to obtaining written informed consent and within 28 days of randomization may be used rather than repeating tests. Eye exam, visual acuity test, and optical coherence tomography (or equivalent diagnostic test) will be assessed by an ophthalmologist at the Screening Visit in all patients. Patients treated with tislelizumab will undergo repeat assessments approximately every 15 weeks (± 7 days)
26. Patients treated with tislelizumab: The ophthalmologic assessments including eye exam, visual acuity test, and optical coherence tomography (or equivalent diagnostic test) should only be performed once at either the EOT or during safety follow-up, within 30 days of study treatment end.
27. Investigators should solicit patients regarding changes in vision, visual disturbance, or ocular inflammation at each scheduled study visit during tislelizumab treatment. For any change in vision, referral to an ophthalmologist will be made for further management guidance. New or worsened clinically significant abnormalities are to be recorded as AEs on the eCRF.
28. Review of AEs and concomitant medications may be conducted by telephone on Days 8 and 15.

Abbreviations: AE, adverse event; AFP, alpha fetoprotein; BID, twice daily; DLCO, diffusing capacity for carbon monoxide; DNA, deoxyribonucleic acid; ECG, electrocardiogram; ECOG, Eastern Cooperative Oncology Group; EORTC QLQ-HCC18, European Organisation for Research and Treatment of Cancer Quality of Life Questionnaire-Hepatocellular Carcinoma-18 Questions; EORTC QLQ-C30, European Organisation for Research and Treatment of Cancer Quality of Life Questionnaire-Core 30; EQ-5D-5L, 3-level European Quality of Life 5-Dimensions (health questionnaire); FEV_1, forced expiratory volume in the first second of expiration; FFPE, formalin-fixed paraffin-embedded; FVC, forced vital capacity; HBcAb, hepatitis B core antibody; HBsAg, hepatitis B surface antigen; HBV, hepatitis B virus; HCV, hepatitis C virus; HBsAb, hepatitis B surface antibody; irAE, immune-related adverse event; IRT, interactive response technology; IV, intravenous; MRI, magnetic resonance imaging; ^{18}F-NaF PET, 18F-sodium fluoride position emission tomography; NCI-CTCAE, National Cancer Institute Common Terminology Criteria for Adverse Events; PK, pharmacokinetic; PO, orally; Q3W, once every 3 weeks; RECIST, Response Evaluation Criteria in Solid Tumors; RNA, ribonucleic acid; SAE, serious adverse event; TEAE, treatment-emergent adverse event; TSH, thyroid stimulating hormone; v, version.

APPENDIX 2. CHILD-PUGH CLASSIFICATION SCORING SYSTEM

The information presented here has been obtained from the Washington University Medical Center, with sources as follows:

- Lucey MR, Brown KA, Everson GT, et al. Minimal criteria for placement of adults on the liver transplant waiting list: a report of a national conference organized by the American Society of Transplant Physicians and the American Association for the Study of Liver Diseases. Liver Transpl Surg. 1997;3(6):628-37.
- Pugh RN, Murray-Lyon IN, Dawson DL, et al. Transection of the oesophagus for bleeding oesophageal varices. Br J Surgery. 1973;60:646-9.
- Trey C, Burns DG, Saunders SJ. Treatment of hepatic coma by exchange blood transfusion. N Engl J Med. 1966;274(9):473-81.

Child-Pugh classification is either Grade A (mild: score 5 to 6 points), B (moderate: from 7 to 9 points), or C (severe: from 10 to 15 points) and is determined by both clinical and biochemical parameters (as shown below).

**Clinical/Biochemical Parameter**	**Score (Anomaly Severity)**		
	**1**	**2**	**3**
Hepatic encephalopathy (NCI-CTCAE Grade)[a]	0[b]	1[c] or 2[d]	3[e] or 4[f]
Ascites (presence and severity)	None	Mild	Moderate
Total bilirubin (mg/dL)	< 2.0	2.0 to 3.0	> 3.0
Serum albumin (g/dL)	> 3.5	2.8 to 3.5	< 2.8
Prothrombin time (seconds prolonged)<br>or<br>(INR[g])	< 4<br>or<br>< 1.7	4 to 6<br>or<br>1.7 to 2.3	> 6<br>or<br>> 2.3

a. Trey C, Burns DG, Saunders SJ. Treatment of hepatic coma by exchange blood transfusion. N Engl J Med. 1966;274(9):473-81.
b. Grade 0: Consciousness, personality, neurological examination, and electrocardiogram are all normal.
c. Grade 1: Restlessness, sleep disorders, irritability/anxiety, hand tremor, writing disorders, 5CPS waves.
d. Grade 2: Lethargy, time barrier, discomfort, asterixis, ataxia, three-phase slow wave.
e. Grade 3: Drowsiness, coma, orientation disorder, over-reflection, stiff/slow wave.
f. Grade 4: Cannot wake up from coma, no independent personality/behavior, irrational, slow 2-3CPS Delta activity.
g. Lucey MR, Brown KA, Everson GT, et al. Minimal criteria for placement of adults on the liver transplant waiting list: a report of a national conference organized by the American Society of Transplant Physicians and the American Association for the Study of Liver Diseases. Liver Transpl Surg. 1997;3(6):628-37.

Abbreviations: INR, international normalized ratio; NCI-CTCAE, National Cancer Institute Common Terminology Criteria for Adverse Events.

APPENDIX 3. THE RESPONSE EVALUATION CRITERIA IN SOLID TUMORS (RECIST) GUIDELINES, VERSION 1.1

The text below was obtained from the following reference:

- Eisenhauer EA, Therasse P, Bogaerts J, Schwartz LH, Sargent D, Ford R, et al. New response evaluation criteria in solid tumours: revised RECIST guideline (Version 1.1). Eur J Cancer. 2009;45:228-247

DEFINITIONS

Response and progression will be evaluated in this trial using the international criteria proposed by the Response Evaluation Criteria in Solid Tumors (RECIST) Committee (Version 1.1). Changes in only the largest diameter (uni-dimensional measurement) of the tumor lesions are used in the RECIST criteria.

Note: Lesions are either measurable or non-measurable using the criteria provided below. The term “evaluable” in reference to measurability will not be used because it does not provide additional meaning or accuracy.

Measurable Disease

Tumor lesions: Must be accurately measured in at least 1 dimension (longest diameter in the plane of measurement is to be recorded) with a minimum size of:

- 10 mm by CT scan (irrespective of scanner type) and MRI (no less than double the slice thickness and a minimum of 10 mm).
- 10 mm caliper measurement by clinical exam (when superficial).
- 20 mm by chest X-ray (if clearly defined and surrounded by aerated lung).

Malignant lymph nodes: To be considered pathologically enlarged and measurable, a lymph node must be ≥ 15 mm in short axis when assessed by CT scan (CT scan slice thickness recommended to be no greater than 5 mm). At baseline and in follow-up, only the short axis will be measured and followed.

Non-measurable Disease

All other lesions (or sites of disease), including small lesions (longest diameter ≥ 10 to < 15 mm with conventional techniques or < 10 mm using spiral CT scan), are considered non-measurable disease. Leptomeningeal disease, ascites, pleural, or pericardial effusion, inflammatory breast disease, lymphangitic involvement of skin or lung, abdominal masses/abdominal organomegaly identified by physical examination that is not measurable by reproducible imaging techniques are all non-measurable.

Bone lesions:

- Bone scan, position emission tomography scan, or plain films are not considered adequate imaging techniques to measure bone lesions. However, these techniques can be used to confirm the presence or disappearance of bone lesions.
- Lytic bone lesions or mixed lytic-blastic lesions, with identifiable soft tissue components, that can be evaluated by cross sectional imaging techniques such as CT

or MRI can be considered as measurable lesions if the soft tissue component meets the definition of measurability described above.

- Blastic bone lesions are non-measurable.

Cystic lesions:

- Lesions that meet the criteria for radiographically defined simple cysts should not be considered as malignant lesions (neither measurable nor non-measurable) since they are, by definition, simple cysts.
- Cystic lesions thought to represent cystic metastases can be considered as measurable lesions, if they meet the definition of measurability described above. However, if non-cystic lesions are present in the same patient, these are preferred for selection as target lesions.

Lesions with prior local treatment:

- Tumor lesions situated in a previously irradiated area, or in an area subjected to other loco-regional therapy, are usually not considered measurable unless there has been demonstrated progression in the lesion. Trial protocols should detail the conditions under which such lesions would be considered measurable.

Target Lesions

All measurable lesions up to a maximum of 2 lesions per organ and 5 lesions in total, should be identified as target lesions and recorded and measured at baseline prior to randomization. Target lesions should be selected on the basis of their size (lesions with the longest diameter), be representative of all involved organ, but in addition should be those that lend themselves to reproducible repeated measurements.

Lymph nodes merit special mention since they are normal anatomical structures which may be visible by imaging even if not involved by tumor. Pathological nodes which are defined as measurable and may be identified as target lesions must meet the criterion of a short axis of ≥ 15 mm by CT scan. Only the short axis of these nodes will contribute to the baseline sum. The short axis of the node is the diameter normally used by radiologists to judge if a node is involved by solid tumor. Nodal size is normally reported as 2 dimensions in the plane in which the image is obtained (for CT scan, this is almost always the axial plane; for MRI the plane of acquisition may be axial, sagittal, or coronal). The smaller of these measures is the short axis. For example, an abdominal node which is reported as being 20 mm × 30 mm has a short axis of 20 mm and qualifies as a malignant, measurable node. In this example, 20 mm should be recorded as the node measurement. All other pathological nodes (those with short axis ≥ 10 mm but < 15 mm) should be considered non-target lesions. Nodes that have a short axis < 10 mm are considered non-pathological and should not be recorded or followed.

A sum of the diameters (longest for non-nodal lesions, short axis for nodal lesions) for all target lesions will be calculated and reported as the baseline sum diameters. If lymph nodes are to be included in the sum, then as noted above, only the short axis is added into the sum. The baseline sum diameters will be used as reference to further characterize any objective tumor regression in the measurable dimension of the disease.

Non-target Lesions

All other lesions (or sites of disease) including pathological lymph nodes should be identified as non-target lesions and should also be recorded at baseline. Measurements are not required and these lesions should be followed as “present”, “absent”, or in rare cases “unequivocal progression” (more details to follow). In addition, it is possible to record multiple non-target lesions involving the same organ as a single item on the case record form (e.g. “multiple enlarged pelvic lymph node” or “multiple liver metastases”).

GUIDELINES FOR EVALUATION OF MEASURABLE DISEASE

All measurements should be recorded in metric notation, using calipers if clinically assessed. All baseline evaluations should be performed as close as possible to the treatment start and never more than 4 weeks before the beginning of the treatment.

The same method of assessment and the same technique should be used to characterize each identified and reported lesion at baseline and during follow-up. Imaging based evaluation should always be done rather than clinical examination unless the lesion(s) being followed cannot be imaged but are accessible by clinical examination.

Clinical lesions: Clinical lesions will only be considered measurable when they are superficial and P10 mm diameter as assessed using calipers (e.g. skin nodules). For the case of skin lesions, documentation by color photography including a ruler to estimate the size of the lesion is suggested. As noted above, when lesions can be evaluated by both clinical examination and imaging, imaging evaluation should be undertaken since it is more objective and may also be reviewed at the end of the trial.

- Chest X-ray: Chest CT is preferred over chest X-ray, particularly when progression is an important endpoint, since CT is more sensitive than X-ray, particularly in identifying new lesions. However, lesions on chest X-ray may be considered measurable if they are clearly defined and surrounded by aerated lung.
- CT, MRI: CT is the best currently available and reproducible method to measure lesions selected for response assessment. This guideline has defined measurability of lesions on CT scan based on the assumption that CT slice thickness is 5 mm or less. When CT scans have slice thickness greater than 5 mm, the minimum size for a measurable lesion should be twice the slice thickness. MRI is also acceptable in certain situations (e.g. for body scans).
- Ultrasound: Ultrasound is not useful in assessment of lesion size and should not be used as a method of measurement. Ultrasound examinations cannot be reproduced in their entirety for independent review at a later date and, because they are operator dependent, it cannot be guaranteed that the same technique and measurements will be taken from one assessment to the next. If new lesions are identified by ultrasound in the course of the study, confirmation by CT or MRI is advised. If there is concern about radiation exposure at CT, MRI may be used instead of CT in selected instances.
- Endoscopy, laparoscopy: The utilization of these techniques for objective tumor evaluation is not advised. However, they can be useful to confirm complete pathological response when biopsies are obtained or to determine relapse in trials where recurrence following complete response or surgical resection is an endpoint.

- Tumor markers: Tumor markers alone cannot be used to assess objective tumor response. If markers are initially above the upper normal limit, however, they must normalize for a patient to be considered in complete response. Because tumor markers are disease specific, instructions for their measurement should be incorporated into protocols on a disease specific basis. Specific guidelines for both CA-125 response (in recurrent ovarian cancer) and prostate-specific antigen response (in recurrent prostate cancer), have been published. In addition, the Gynecologic Cancer Intergroup has developed CA-125 progression criteria which are to be integrated with objective tumor assessment for use in first-line trials in ovarian cancer.
- Cytology, histology: These techniques can be used to differentiate between PR and CR in rare cases if required by protocol (for example, residual lesions in tumor types such as germ cell tumors, where known residual benign tumors can remain). When effusions are known to be a potential adverse effect of treatment (e.g. with certain taxane compounds or angiogenesis inhibitors), the cytological confirmation of the neoplastic origin of any effusion that appears or worsens during treatment can be considered if the measurable tumor has met criteria for response or stable disease (SD) in order to differentiate between response (or SD) and progressive disease.

RESPONSE CRITERIA

Evaluation of Target Lesions

- Complete Response (CR): Disappearance of all target lesions. Any pathological lymph nodes (whether target or non-target) must have reduction in short axis to < 10 mm.
- Partial Response (PR): At least a 30% decrease in the sum of diameters of target lesions, taking as reference the baseline sum diameters.
- Progressive Disease (PD): At least a 20% increase in the sum of diameters of target lesions, taking as reference the smallest sum on study (this includes the baseline sum if that is the smallest on study). In addition to the relative increase of 20%, the sum must also demonstrate an absolute increase of at least 5 mm. (Note: the appearance of one or more new lesions is also considered progression).
- Stable Disease (SD): Neither sufficient shrinkage to qualify for PR nor sufficient increase to qualify for PD, taking as reference the smallest sum diameters while on study.
- Lymph nodes: Lymph nodes identified as target lesions should always have the actual short axis measurement recorded (measured in the same anatomical plane as the baseline examination), even if the nodes regress to below 10 mm on study. This means that when lymph nodes are included as target lesions, the “sum” of lesions may not be zero even if complete response criteria are met, since a normal lymph node is defined as having a short axis of < 10 mm. Case report recorded in a separate section where, in order to qualify for CR, each node must achieve a short axis < 10 mm. For PR, SD, and PD, the actual short axis measurement of the nodes is to be included in the sum of target lesions.

- Target lesions that become "too small to measure". While on study, all lesions (nodal and non-nodal) recorded at baseline should have their actual measurements recorded at each subsequent evaluation, even when very small (e.g. 2 mm). However, sometimes lesions or lymph nodes which are recorded as target lesions at baseline become so faint on CT scan that the radiologist may not feel comfortable assigning an exact measure and may report them as being "too small to measure". When this occurs, it is important that a value be recorded on the eCRF. If it is the opinion of the radiologist that the lesion has likely disappeared, the measurement should be recorded as 0 mm. If the lesion is believed to be present and is faintly seen but too small to measure, a default value of 5 mm should be assigned (Note: It is less likely that this rule will be used for lymph nodes since they usually have a definable size when normal and are frequently surrounded by fat such as in the retroperitoneum; however, if a lymph node is believed to be present and is faintly seen but too small to measure, a default value of 5 mm should be assigned in this circumstance as well). This default value is derived from the 5 mm CT slice thickness (but should not be changed with varying CT slice thickness). The measurement of these lesions is potentially non-reproducible, therefore providing this default value will prevent false responses or progressions based upon measurement error. To reiterate, however, if the radiologist is able to provide an actual measure, that should be recorded, even if it is below 5 mm.
- Lesions that split or coalesce on treatment: When non-nodal lesions "fragment", the longest diameters of the fragmented portions should be added together to calculate the target lesion sum. Similarly, as lesions coalesce, a plane between them may be maintained that would aid in obtaining maximal diameter measurements of each individual lesion. If the lesions have truly coalesced such that they are no longer separable, the vector of the longest diameter in this instance should be the maximal longest diameter for the "coalesced lesion".

Evaluation of Non-target Lesions

While some non-target lesions may actually be measurable, they need not be measured and instead should be assessed only qualitatively at the time points specified in the protocol.

- CR: Disappearance of all non-target lesions and normalization of tumor marker level. All lymph nodes must be non-pathological in size (< 10 mm short axis).
- PD: Unequivocal progression (as detailed below) of existing non-target lesions. (Note: the appearance of one or more new lesions is also considered progression).
- Non-CR/Non-PD: Persistence of one or more non-target lesion(s) and/or maintenance of tumor marker level above the normal limits.
- When the patient also has measurable disease: In this setting, to achieve "unequivocal progression" on the basis of the non-target disease, there must be an overall level of substantial worsening in non-target disease such that, even in presence of SD or PR in target disease, the overall tumor burden has increased sufficiently to merit discontinuation of therapy. A modest "increase" in the size of one or more non-target lesions is usually not sufficient to qualify for unequivocal progression status. The

designation of overall progression solely on the basis of change in non-target disease in the face of SD or PR of target disease will therefore be extremely rare.

- When the patient has only non-measurable disease: This circumstance arises in some Phase 3 trials when it is not a criterion of trial entry to have measurable disease. The same general concept apply here as noted above, however, in this instance there is no measurable disease assessment to factor into the interpretation of an increase in non-measurable disease burden. Because worsening in non-target disease cannot be easily quantified (by definition: if all lesions are truly non-measurable) a useful test that can be applied when assessing patients for unequivocal progression is to consider if the increase in overall disease burden based on the change in non-measurable disease is comparable in magnitude to the increase that would be required to declare PD for measurable disease: i.e., an increase in tumor burden representing an additional 73% increase in “volume” (which is equivalent to a 20% increase diameter in a measurable lesion).

Examples include an increase in a pleural effusion from “trace” to “large”, an increase in lymphangitic disease from localized to widespread, or may be described in protocols as “sufficient to require a change in therapy”. If “unequivocal progression” is seen, the patient should be considered to have had overall PD at that point. While it would be ideal to have objective criteria to apply to non-measurable disease, the very nature of that disease makes it impossible to do so, therefore the increase must be substantial.

New Lesions

The appearance of new malignant lesions denotes disease progression; therefore, some comments on detection of new lesions are important. There are no specific criteria for the identification of new radiographic lesions; however, the finding of a new lesion should be unequivocal: i.e. not attributable to differences in scanning technique, change in imaging modality or findings thought to represent something other than tumor (for example, some “new” bone lesions may be simply healing or flare of pre-existing lesions). This is particularly important when the patient’s baseline lesions show partial or complete response. For example, necrosis of a liver lesion may be reported on a CT scan report as a “new” cystic lesion, which it is not.

A lesion identified on a follow-up trial in an anatomical location that was not scanned at baseline is considered a new lesion and will indicate disease progression. An example of this is the patient who has visceral disease at baseline and while on trial has a CT or MRI brain ordered which reveals metastases. The patient’s brain metastases are considered to be evidence of PD even if he/she did not have brain imaging at baseline.

If a new lesion is equivocal, for example because of its small size, continued therapy and follow-up evaluation will clarify if it represents truly new disease. If repeat scans confirm there is definitely a new lesion, then progression should be declared using the date of the initial scan.

While fluorodeoxyglucose-position emission tomography (FDG-PET) response assessments need additional study, it is sometimes reasonable to incorporate the use of FDG-PET scanning to complement CT scanning in assessment of progression (particularly possible “new” disease). New lesions on the basis of FDG-PET imaging can be identified according to the following algorithm:

- Negative FDG-PET at baseline, with a positive FDG-PET at follow-up: This is a sign of PD based on a new lesion.
- No FDG-PET at baseline and a positive FDG-PET at follow-up: If the positive FDG-PET at follow-up corresponds to a new site of disease confirmed by CT, this is PD. If the positive FDG-PET at follow-up is not confirmed as a new site of disease on CT, additional follow-up CT scans are needed to determine if there is truly progression occurring at that site (if so, the date of PD will be the date of the initial abnormal FDG-PET scan). If the positive FDG-PET at follow-up corresponds to a pre-existing site of disease on CT that is not progressing on the basis of the anatomic images, this is not PD.

Evaluation of Best Overall Response

The best overall response is the best response recorded from the start of the study drug treatment until the end of treatment taking into account any requirement for confirmation. On occasion a response may not be documented until after the end of therapy so protocols should be clear if post-treatment assessments are to be considered in determination of best overall response. Protocols must specify how any new therapy introduced before progression will affect best response designation. The patient’s best overall response assignment will depend on the findings of both target and non-target disease and will also take into consideration the appearance of new lesions. Furthermore, depending on the nature of the trial and the protocol requirements, it may also require confirmatory measurement. Specifically, in non-randomized trials where response is the primary endpoint, confirmation of PR or CR is needed to deem either one the “best overall response”.

The best overall response is determined once all the data for the patient is known. Best response determination in trials where confirmation of complete or partial response IS NOT required: Best response in these trials is defined as the best response across all time points (for example, a patient who has SD at first assessment, PR at second assessment, and PD on last assessment has a best overall response of PR). When SD is believed to be best response, it must also meet the protocol specified minimum time from baseline. If the minimum time is not met when SD is otherwise the best time point response, the patient’s best response depends on the subsequent assessments. For example, a patient who has SD at first assessment, PD at second and does not meet minimum duration for SD, will have a best response of PD. The same patient lost to follow-up after the first SD assessment would be considered inevaluable.

Target Lesions	Non-target Lesions	New Lesions	Overall Response
CR	CR	No	CR
CR	Non-CR/non-PD	No	PR
CR	Not evaluated	No	PR
PR	Non-PD or not all evaluated	No	PR
SD	Non-PD or not all evaluated	No	SD
Not all evaluated	Non-PD	No	NE
PD	Any	Yes or No	PD
Any	PD	Yes or No	PD
Any	Any	Yes	PD

Abbreviations: CR, complete response; NE, not evaluable; PD, progressive disease; PR, partial response; SD, stable disease.

When nodal disease is included in the sum of target lesions and the nodes decrease to "normal" size (< 10 mm), they may still have a measurement reported on scans. This measurement should be recorded even though the nodes are normal in order not to overstate progression should it be based on increase in size of the nodes. As noted earlier, this means that patients with CR may not have a total sum of "zero" on the eCRF.

In trials where confirmation of response is required, repeated 'NE' time point assessments may complicate best response determination. The analysis plan for the trial must address how missing data/assessments will be addressed in determination of response and progression. For example, in most trials it is reasonable to consider a patient with time point responses of PR-NE-PR as a confirmed response.

Patients with a global deterioration of health status requiring discontinuation of treatment without objective evidence of disease progression at that time should be reported as "symptomatic deterioration". Every effort should be made to document objective progression even after discontinuation of treatment. Symptomatic deterioration is not a descriptor of an objective response: it is a reason for stopping trial therapy.

Conditions that define "early progression, early death, and inevaluability" are trial specific and should be clearly described in each protocol (depending on treatment duration, treatment periodicity).

In some circumstances it may be difficult to distinguish residual disease from normal tissue. When the evaluation of complete response depends upon this determination, it is recommended that the residual lesion be investigated (fine needle aspirate/biopsy) before assigning a status of complete response. FDG-PET may be used to upgrade a response to a CR in a manner similar to a biopsy in cases where a residual radiographic abnormality is thought to represent fibrosis or scarring. The use of FDG-PET in this circumstance should be prospectively described in the protocol and supported by disease specific medical literature for the indication. However, it must be acknowledged that both approaches may lead to false positive CR due to limitations of FDG-PET and biopsy resolution/ sensitivity.

For equivocal findings of progression (e.g. very small and uncertain new lesions; cystic changes, or necrosis in existing lesions), treatment may continue until the next scheduled assessment. If at the next scheduled assessment, progression is confirmed, the date of progression should be the earlier date when progression was suspected.

CONFIRMATORY MEASUREMENT/DURATION OF RESPONSE

Confirmation

In non-randomized trials where response is the primary endpoint, confirmation of PR and CR is required to ensure responses identified are not the result of measurement error. This will also permit appropriate interpretation of results in the context of historical data where response has traditionally required confirmation in such trials. However, in all other circumstances, i.e. in randomized trials (Phase 2 or 3) or trials where SD or progression are the primary endpoints, confirmation of response is not required since it will not add value to the interpretation of trial results. However, elimination of the requirement for response confirmation may increase the importance of central review to protect against bias, in particular in trials which are not blinded.

In the case of SD, measurements must have met the SD criteria at least once after trial entry at a minimum interval (in general not less than 6 weeks).

Duration of Overall Response

The duration of overall response is measured from the time measurement criteria are first met for CR/PR (whichever is first recorded) until the first date that recurrent or progressive disease is objectively documented (taking as reference for progressive disease the smallest measurements recorded on study).

The duration of overall complete response is measured from the time measurement criteria are first met for CR until the first date that recurrent disease is objectively documented.

Duration of Stable Disease

Stable disease is measured from the start of the treatment (in randomised trials, from date of randomization) until the criteria for progression are met, taking as reference the smallest sum on study (if the baseline sum is the smallest, this is the reference for calculation of PD).

The clinical relevance of the duration of SD varies in different studies and diseases. If the proportion of patients achieving SD for a minimum period of time is an endpoint of importance in a particular trial, the protocol should specify the minimal time interval required between 2 measurements for determination of stable disease.

Note: The duration of response and stable disease as well as the progression-free survival are influenced by the frequency of follow-up after baseline evaluation. It is not in the scope of this guideline to define a standard follow-up frequency. The frequency should take into account many parameters including disease types and stages, treatment periodicity, and standard practice. However, these limitations of the precision of the measured endpoint should be taken into account if comparisons between trials are to be made.

APPENDIX 4. PRE-EXISTING IMMUNE DEFICIENCIES OR AUTOIMMUNE DISEASES

Prospective patients should be carefully questioned to determine whether they have any history of an acquired or congenital immune deficiency or autoimmune disease.

Please contact the sponsor Medical Monitor regarding any uncertainty about immune deficiency/autoimmune disease exclusions.

Acute disseminated encephalomyelitis	Addison's disease
Ankylosing spondylitis	Antiphospholipid antibody syndrome
Aplastic anemia	Autoimmune hemolytic anemia
Autoimmune hepatitis	Autoimmune hypoparathyroidism
Autoimmune hypophysitis	Autoimmune myocarditis
Autoimmune oophoritis	Autoimmune orchitis
Autoimmune thrombocytopenic purpura	Behcet's disease
Bullous pemphigoid	Chronic inflammatory demyelinating polyneuropathy
Chung-Strauss syndrome	Crohn's disease
Dermatomyositis	Dysautonomia
Epidermolysis bullosa acquisita	Gestational pemphigoid
Giant cell arteritis	Goodpasture's syndrome
Granulomatosis with polyangiitis	Graves' disease
Guillain-Barré syndrome	Hashimoto's disease
Immunoglobulin A (IgA) neuropathy	Interstitial cystitis
Inflammatory bowel disease	Lambert-Eaton myasthenia syndrome
Kawasaki's disease	Lyme disease (chronic)
Lupus erythematosus	Morphea
Mooren's ulcer	Myasthenia gravis
Multiple sclerosis	Opsoclonus myoclonus syndrome
Neuromyotonia	Ord's thyroiditis
Optic neuritis	Pernicious anemia
Pemphigus	Polyarthritis
Polyarteritis nodusa	Primary biliary cirrhosis
Polyglandular autoimmune syndrome	Reiter's syndrome
Psoriasis	Sarcoidosis
Rheumatoid arthritis	Stiff person syndrome
Sjögren's syndrome	Ulcerative colitis
Takayasu's arteritis	Vogt-Kovangai-Harada disease

APPENDIX 5. CLINICAL LABORATORY ASSESSMENTS

Serum Chemistry	Hematology	Coagulation	Urinalysis
Alkaline phosphatase<br>Alanine aminotransferase<br>Aspartate aminotransferase<br>Albumin<br>Direct bilirubin<br>Total bilirubin<br>Blood urea nitrogen or urea<br>Creatinine<br>Glucose<br>Lactate dehydrogenase<br>Total protein<br>Potassium<br>Sodium<br>Calcium<br>Magnesium<br>Phosphate[4]<br>Creatine kinase[2]<br>Creatine kinase cardiac muscle isoenzyme (CK-MB)[2,3]	Hemoglobin<br>Hematocrit<br>Platelet count<br>WBC count<br>Neutrophil count<br>Lymphocyte count	Prothrombin time<br>Activated Partial Thromboplastin Time<br>International Normalized Ratio	Glucose<br>Protein<br>Ketones<br>Blood<br>24 hour protein[1] Random urine protein to creatinine ratio

Abbreviations: WBC=white blood cell.

Note: Additional laboratory assessments may be conducted if required for clinical management; relevant data from those assessments will be collected by the sponsor.

1. On dipstick urinalysis, if urine protein is ≥2+, then obtain a 24-hour urine sample for total protein and a random urine sample for total protein and creatinine to determine a protein to creatinine ratio
2. For all patients during screening visits, and for all patients randomized to receive tislelizumab at scheduled visits during the first 3 treatment cycles, all predose assessments from Cycle 4 onwards, and at the end of treatment and safety follow-up visits.
3. In event CK-MB fractionation is not available, please assess troponin I and/or troponin T instead
4. Measured with a serum phosphorus or serum phosphate test depending on the laboratory.

APPENDIX 6. CONTRACEPTION GUIDELINES AND DEFINITIONS OF "WOMEN OF CHILDBEARING POTENTIAL", "NO CHILDBEARING POTENTIAL"

Contraception Guidelines

The Clinical Trials Facilitation Group's recommendations related to contraception and pregnancy testing in clinical trials include the use of highly effective forms of birth control. These methods include the following (availability of contraception specified below will vary depending upon approval status in each country):

- Combined (estrogen- and progestogen-containing) hormonal contraception associated with the inhibition of ovulation (oral, intravaginal, or transdermal)
    - Note: The following oral medications are available in Japan: Ortho M-21, Marvelon, Favoir, Ortho 777, Synphase T28, Triquilar 21/28, Ange 21/28, Labellefille 21/28, Marvelon 28
- Progestogen-only hormonal contraception associated with the inhibition of ovulation (oral, injectable, or implantable) Note: Progesterone-only hormonal contraception is not available in Japan
- Intrauterine device (IUD) Note: Multiload CU250R & NovaT is available in Japan
- Intrauterine hormone-releasing system (IUS) Note: Mirena is available in Japan
- Bilateral tubal occlusion
- Vasectomized male partner provided that the vasectomized partner is the sole sexual partner of the woman of childbearing potential study participant and that the vasectomized partner has received medical assessment of surgical success.
- Sexual abstinence (defined as refraining from heterosexual intercourse during the entire period of exposure associated with the study treatment).
    - NOTE: Total sexual abstinence should only be used as a contraceptive method if it is in line with the patient's usual and preferred lifestyle. Periodic abstinence (eg, calendar, ovulation, symptothermal, post-ovulation methods), declaration of abstinence for the duration of exposure to study drug, and withdrawal are not acceptable methods of contraception.

Of note, barrier contraception (including male and female condoms with or without spermicide) is not considered a highly effective method of contraception and if used, this method must be combined with a highly effective form of birth control listed above.

Definitions of "Women of Childbearing Potential," "Women of No Childbearing Potential"

As defined in this protocol, "women of childbearing potential" are female patients who are physiologically capable of becoming pregnant (applicable to both study participants and sexual partners of male participants).

Conversely, "women of no childbearing potential" are defined as female patients meeting any of the following criteria:

- Surgically sterile (ie, through bilateral salpingectomy, bilateral oophorectomy, or hysterectomy)
- Postmenopausal, for no other medical reasons, defined as:
  - ≥ 55 years of age with no spontaneous menses for ≥ 12 months OR
  - < 55 years of age with no spontaneous menses for ≥ 12 months AND with postmenopausal
    follicle stimulating hormone concentration > 30 IU/mL and all alternative medical causes for the lack of spontaneous menses for ≥ 12 months have been ruled out, such as polycystic ovarian syndrome, hyperprolactinemia, etc.
  - If an FSH measurement is required to confirm postmenopausal state, concomitant use of hormonal contraception or hormonal replacement therapy should be excluded.

Definitions of "Sterile Male"

A sterile male is defined as one for whom azoospermia, in a semen sample examination, has been demonstrated as definitive evidence of infertility. Males with 'low sperm counts' (consistent with 'sub-fertility) are not to be considered sterile for purposes of this study.

Adapted from Clinical Trials Facilitation Group (CTFG). Recommendations related to contraception and pregnancy testing in clinical trials. September 15, 2014. http://www.hma.eu/fileadmin/dateien/Human_Medicines/01-About_HMA/Working_Groups/CTFG/2014_09_HMA_CTFG_Contraception.pdf.

APPENDIX 7. NEW YORK HEART ASSOCIATION FUNCTIONAL CLASSIFICATION

Class	Symptoms
I	No limitation of physical activity. Ordinary physical activity does not cause undue fatigue, palpitation, dyspnea (shortness of breath).
II	Slight limitation of physical activity. Comfortable at rest, but ordinary physical activity results in fatigue, palpitation, dyspnea (shortness of breath).
III	Marked limitation of physical activity. Comfortable at rest, but less than ordinary activity causes fatigue, palpitation, or dyspnea.
IV	Unable to carry on any physical activity without discomfort. Symptoms of heart failure at rest. If any physical activity is undertaken, discomfort increases.

Adapted from Dolgin M, Association NYH, Fox AC, Gorlin R, Levin RI, New York Heart Association. Criteria Committee. Nomenclature and criteria for diagnosis of diseases of the heart and great vessels. 9th ed. Boston, MA: Lippincott Williams and Wilkins; March 1, 1994.
Original source: Criteria Committee, New York Heart Association, Inc. Diseases of the Heart and Blood Vessels. Nomenclature and Criteria for diagnosis, 6th edition Boston, Little, Brown and Co. 1964, p 114.

APPENDIX 8. CHRONIC KIDNEY DISEASE EPIDEMIOLOGY COLLABORATION (CKD-EPI) EQUATION

In adults, the most widely-used equations for estimating glomerular filtration rate (GFR) from serum creatinine are the Chronic Kidney Disease Epidemiology Collaboration (CKD-EPI) equation and the Modification of Diet in Renal Disease (MDRD) Study equation[1]. The National Kidney Disease Education Program (NKDEP) calculators rely on creatinine determinations which are isotope dilution mass spectrometry (IDMS) traceable. All laboratories should be using creatinine methods calibrated to be IDMS traceable.

The CKD-EPI equation calculator should be used when serum creatinine (S_{cr}) reported in mg/dL. This equation is recommended when eGFR values above 60 mL/min/1.73 m^2 are desired.

GFR = $141 \times \min(S_{cr}/\kappa, 1)^{\alpha} \times \max(S_{cr}/\kappa, 1)^{-1.209} \times 0.993^{Age} \times 1.018$ [if female] $\times 1.159$ [if black]

where:

S_{cr} is serum creatinine in mg/dL,

κ is 0.7 for females and 0.9 for males,

α is -0.329 for females and -0.411 for males,

min indicates the minimum of S_{cr}/κ or 1, and

max indicates the maximum of S_{cr}/κ or 1.

The equation does not require weight because the results are reported normalized to 1.73 m^2 body surface area, which is an accepted average adult surface area.

The online calculator for CKD-EPI can be found here: https://www.niddk.nih.gov/health-information/health-communication-programs/nkdep/laboratory-evaluation/glomerular-filtration-rate-calculators/Pages/gfr-calculators.aspx

Levey AS, Stevens LA, Schmid CH, et al. A new equation to estimate glomerular filtration rate. Ann Intern Med. 2009;150(9):604-12

APPENDIX 9. DOSE MODIFICATION GUIDELINE FOR SORAFENIB

Dose modifications for sorafenib will be performed according to the investigator's clinical judgement and consistent with the prescribing information, using the details in this appendix as guidance. Also see Section 5.5.2 for additional guidelines on dose modifications for sorafenib.

For dermatologic toxicities, dose modifications are recommended by following the actions in the table below.

Recommended Dose Modifications for Dermatologic Toxicities of Sorafenib

Dermatologic Toxicity Grade	Occurrence	Suggested Dose Modification
Grade 1: Numbness, dysesthesia, paresthesia, tingling, painless swelling, erythema or discomfort of the hands or feet which does not disrupt the patient's normal activities	Any occurrence	Continue treatment with sorafenib and consider topical therapy for symptomatic relief
Grade 2: Painful erythema and swelling of the hands or feet and/or discomfort affecting the patient's normal activities	1st occurrence	Continue treatment with sorafenib and consider topical therapy for symptomatic relief<br>If no improvement within 7 days, see below
	No improvement within 7 days or 2nd or 3rd occurrence	Interrupt sorafenib treatment until toxicity resolves to Grade 0–1<br>When resuming treatment, decrease sorafenib dose by 1 dose level (400 mg daily or 400 mg every other day)
	4th occurrence	Discontinue sorafenib treatment
Grade 3: Moist desquamation, ulceration, blistering or severe pain of the hands or feet, or severe discomfort that causes the patient to be unable to work or perform activities of daily living	1st or 2nd occurrence	Interrupt sorafenib treatment until toxicity resolves to Grade 0–1<br>When resuming treatment, decrease sorafenib dose by 1 dose level (400 mg daily or 400 mg every other day)
	3rd occurrence	Discontinue sorafenib treatment

For toxicities not listed above, dose modifications are permitted per local standards after discussion and agreement with sponsor medical monitor.

Dose modifications or delays may occur in the setting of a lower grade adverse event if the investigator, in consultation with sponsor medical monitor, believes that it is in the interest of a patient's safety.

For toxicity except dermatologic toxicity, dose modifications are recommended by following the actions in the table below.

Recommended Dose Modifications for Toxicities except for Dermatologic Toxicities of Sorafenib

Grade	Dose Delay/Interruption	Dose Modification
Hematological toxicities		
Grade 0-2	Continue treatment	No change
Grade 3	Continue treatment	Decrease 1 dose level
Grade 4	Delay[1] until ≤ Grade 2	Decrease 1 dose level
Non-hematological toxicities (except skin toxicity[2])		
Grade 0-2	Continue treatment	No change
Grade 3	Delay[1] until ≤ Grade 2	Decrease 1 dose level[3]
Grade 4	Permanently discontinue	Permanently discontinue

1. If no recovery after 30-day delay, treatment will be discontinued unless patient is deriving clinical benefit
2. Also excludes nausea/vomiting that has not been premedicated, and diarrhea
3. If more than 2 dose reductions are required, treatment will be discontinued.

CRITERIA FOR DISCONTINUING SORAFENIB:

Discontinuation of sorafenib is required for the following:

- Cardiac ischemia or infarction
- Hemorrhage requiring medical intervention
- Severe or persistent hypertension despite adequate antihypertensive therapy
- Gastrointestinal perforation
- QT interval corrected for heart rate (QTc) is greater than 500 milliseconds or for an increase from baseline of 60 milliseconds or greater
- Severe drug-induced liver injury, defined as elevated transaminase levels (above 10 times the upper limit of normal [ULN]) or transaminase elevations with EITHER elevated bilirubin (above 5 times the ULN) OR international normalized ratio > 4
- Undergoing major surgical procedures

For toxicities not listed above, the investigator should use their medical judgement to determine whether sorafenib should be discontinued, in accordance with patient’s well-being and local standards.

APPENDIX 10. IMMUNE-RELATED ADVERSE EVENT EVALUATION AND MANAGEMENT

The recommendations below for the diagnosis and management of any irAE are intended as a guidance. This document should be used in conjunction with expert clinical judgement (by specialist physicians experienced in the treatment of cancer using immunological agents), and individual institutional guidelines or policies.

Criteria used to diagnose irAEs include blood tests, diagnostic imaging, histopathology, and microbiology assessments to exclude alternative causes such as infection, disease progression, and adverse effects of concomitant drugs. In addition to the results of these tests, the following factors should be considered when making an irAE diagnosis:

- What was the temporal relationship between initiation of tislelizumab and the adverse event?
- How did the patient respond to withdrawal of tislelizumab?
- Did the event recur when tislelizumab was reintroduced?
- Was there a clinical response to corticosteroids?
- Is the event an autoimmune endocrinopathy?
- Is disease progression or an alternative diagnosis a more likely explanation?

When alternative explanations to autoimmune toxicity have been excluded, the irAE field, associated with the AE in the eCRF should be checked.

**Recommended Diagnostic Tests in the Management of Possible Immune-related Adverse Events**	
**Immune-related Toxicity**	**Diagnostic Evaluation Guideline**
Thyroid Disorders	Scheduled and repeat thyroid function tests (TSH and T4).
Hypophysitis	Check visual fields and consider pituitary endocrine axis blood profile. Perform pituitary and whole brain MRI in patients with headache, visual disturbance, unexplained fatigue, asthenia, weight loss and unexplained constitutional symptoms.<br>Consider consultation with an endocrinologist if an abnormality is detected.
Pneumonitis	All patients presenting with new or worsened pulmonary symptoms or signs, such as an upper respiratory infection, new cough, shortness of breath or hypoxia should be assessed by high-resolution CT. Consider pulmonary function test including DLCO.<br>Radiographic appearance is often nonspecific. Depending on the location of the abnormality, bronchoscopy and bronchoalveolar lavage or lung biopsy may be considered. Consult with a respiratory medicine physician for cases of uncertain cause.
Neurological Toxicity	Perform a comprehensive neurological examination and brain MRI for all CNS symptoms; review alcohol history and other medications. Conduct a diabetic screen, and assess blood B12/folate, HIV status, TFTs, and consider autoimmune serology. Consider the need for brain/spine

Recommended Diagnostic Tests in the Management of Possible Immune-related Adverse Events	
**Immune-related Toxicity**	**Diagnostic Evaluation Guideline**
	MRI/MRA and nerve conduction study for peripheral neuropathy. Consult with a neurologist if there are abnormal findings.
Colitis	Review dietary intake and exclude steatorrhea. Consider comprehensive testing, including the following: FBC, UEC, LFTs, CRP, TFTs, stool microscopy and culture, viral PCR, Clostridium difficile toxin, cryptosporidia (drug-resistant organism).<br>In case of abdominal discomfort, consider imaging, eg, X-ray, CT scan. If a patient experiences bleeding, pain or distension, consider colonoscopy with biopsy and surgical intervention, as appropriate.
Eye Disorders	If patients experience acute, new onset, or worsening of eye inflammation, blurred vision or other visual disturbance, refer the patient urgently to an ophthalmologist for evaluation and management.
Hepatitis	Check ALT/AST/total bilirubin, INR/albumin; the frequency will depend on severity of the AE (eg, daily if Grade 3-4; every 2-3 days if Grade 2, until recovering). Review medications (eg, statins, antibiotics) and alcohol history. Perform liver screen including Hepatitis A/B/C serology, Hepatitis E PCR and assess anti-ANA/SMA/LKM/SLA/LP/LCI, iron studies. Consider imaging, eg, ultrasound scan for metastases or thromboembolism. Consult with a hepatologist and consider liver biopsy.
Renal toxicity	Review hydration status, and medication history. Test and culture urine. Consider renal ultrasound scan, protein assessment (dipstick/24-hour urine collection), or phase-contrast microscopy. Refer to nephrology for further management assistance.
Dermatology	Consider other causes by conducting a physical examination, consider dermatology referral for skin biopsy
Joint or muscle inflammation	Conduct musculoskeletal history and perform complete musculoskeletal examination. Consider joint X-ray and other imaging as required to exclude metastatic disease. Perform autoimmune serology and refer to rheumatology for further management assistance.<br>For suspected myositis/rhabdomyolysis/myasthenia include: CK, ESR, CRP, troponin and consider a muscle biopsy.
Myocarditis	Perform ECG, CK/CK-MB, echocardiogram, troponin (I and/or T), and refer to a cardiologist.

Abbreviations: AE, adverse event; ALT, alanine aminotransferase; ANA, antinuclear antibody; AST, aspartate aminotransferase; CK, creatine kinase; CK-MB, creatine kinase cardiac muscle isoenzyme; CNS, central nervous system; CRP, C-reactive protein; CT, computed tomography; DLCO, diffusing capacity for carbon monoxide; ECG, electrocardiogram, ESR, erythrocyte sedimentation rate; FBC, full blood count; HIV, human immunodeficiency virus; INR, international normalized ratio; LCI, liver cytosolic antigen; LFT, liver function test; LKM, liver kidney microsomal antibody; LP, liver pancreas antigen; MRA, magnetic resonance angiogram; MRI, magnetic resonance imaging; PCR, polymerase chain reaction; SLA, soluble liver antigen; SMA, smooth muscle antibody; T4, thyroxine; TFT, thyroid function tests; TSH, thyroid-stimulating hormone; UEC, urea electrolytes and creatinine.

Treatment of Immune-related Adverse Events

- Immune-related AEs can escalate quickly; Study treatment interruption, close monitoring, timely diagnostic work-up and treatment intervention, as appropriate, with patients is required
- Immune-related AEs should improve promptly after introduction of immunosuppressive therapy. If this does not occur, review the diagnosis, seek further specialist advice and contact the study medical monitor
- For some Grade 3 toxicities that resolve quickly, rechallenge with study drug may be considered if there is evidence of a clinical response to study treatment, after consultation with the study medical monitor
- Steroid dosages in the table below are for oral or intravenous (methyl)prednisolone. Equivalent dosages of other corticosteroids can be substituted. For steroid-refractory irAEs, consider steroid-sparing agents (eg, mycophenolate mofetil [MMF])
- Consider prophylactic antibiotics for opportunistic infections if the patient is receiving long-term immunosuppressive therapy

Autoimmune Toxicity	Grade	Treatment Guidelines (Subject to Clinical Judgement)	Study Drug Management
**Thyroid Disorders**	**1-2**<br>Asymptomatic TFT abnormality or mild symptoms	Replace thyroxine if hypothyroid, until TSH/T4 levels return to normal range.<br>Thyrotoxic patients should be referred to an endocrinologist. In cases with systemic symptoms: withhold study treatment, treat with a beta blocker and consider oral prednisolone 0.5 mg/kg/day for thyroid pain. Taper corticosteroids over 2-4 weeks. Monitor thyroid function regarding the need for hormone replacement.	Continue study treatment or withhold treatment in cases with systemic symptoms.
	**3-4**<br>Severe symptoms, hospitalization required	Refer patient to an endocrinologist.<br>If hypothyroid, replace with thyroxine 0.5-1.6 µg/kg/day (for the elderly or those with co-morbidities, the suggested starting dose is 0.5 µg/kg/day). Add oral prednisolone 0.5 mg/kg/day for thyroid pain. Thyrotoxic patients require treatment with a beta blocker and may require carbimazole until thyroiditis resolves.	Hold study treatment; resume when resolved/improved to Grade 0-1.

Autoimmune Toxicity	Grade	Treatment Guidelines (Subject to Clinical Judgement)	Study Drug Management
**Hypophysitis**	**1-2**<br>Mild-moderate symptoms	Refer patient to an endocrinologist for hormone replacement. Add oral prednisolone 0.5-1 mg/kg/day for patients with pituitary inflammation. Taper corticosteroids over at least 1 month. If there is no improvement in 48 hours, treat as Grade 3-4.	Continue study treatment.
	**3-4**<br>Severe or life-threatening symptoms	Refer patient to an endocrinologist for assessment and treatment. Initiate pulse IV methylprednisolone 1 mg/kg for patients with headache/visual disturbance due to pituitary inflammation. Convert to oral prednisolone and taper over at least 1 month. Maintain hormone replacement according to endocrinology advice.	Hold study treatment for patients with headache/visual disturbance due to pituitary inflammation until resolved/improved to Grade 2 or less. Discontinuation is usually not necessary.
**Pneumonitis**	**1**<br>Radiographic changes only	Monitor symptoms every 2-3 days.<br>If appearance worsens, treat as Grade 2.	Consider holding study treatment until appearance improves and cause is determined.
	**2**<br>Symptomatic: exertional breathlessness	Commence antibiotics if infection suspected. Add oral prednisolone 1 mg/kg/day if symptoms/appearance persist for 48 hours or worsen. Consider Pneumocystis infection prophylaxis. Taper corticosteroids over at least 6 weeks.<br>Consider prophylaxis for adverse steroid effects: eg, blood glucose monitoring, vitamin D/calcium supplement.	Hold study treatment. Retreatment is acceptable if symptoms resolve completely or are controlled on prednisolone ≤ 10 mg/day. Discontinue study treatment if symptoms persist with corticosteroid treatment.
	**3-4**<br>Severe or life-threatening symptoms<br>Breathless at rest	Admit to a hospital and initiate treatment with IV methylprednisolone 2-4 mg/kg/day. If there is no improvement, or worsening after 48 hours, add infliximab 5 mg/kg (if no hepatic involvement). Convert to oral prednisolone and taper over at least 2 months. Cover with empiric antibiotics and consider prophylaxis for Pneumocystis infection and other adverse steroid effects, eg, blood glucose monitoring, vitamin D/calcium supplement.	Discontinue study treatment.
**Neurological Toxicity**	**1**<br>Mild symptoms	--	Continue study treatment.

Autoimmune Toxicity	Grade	Treatment Guidelines (Subject to Clinical Judgement)	Study Drug Management
	**2**<br>Moderate symptoms	Treat with oral prednisolone 0.5-1 mg/kg/day. Taper over at least 4 weeks. Obtain neurology consultation.	Hold study treatment; resume when resolved/improved to Grade 0-1.
	**3-4**<br>Severe/life-threatening symptoms	Initiate treatment with oral prednisolone or IV methylprednisolone 1-2 mg/kg/day, depending on symptoms. Taper corticosteroids over at least 4 weeks.<br>Consider azathioprine, MMF, cyclosporine if no response within 72- 96 hours.	Discontinue study treatment.
**Colitis/ Diarrhea**	**1**<br>Mild symptoms: < 3 liquid stools per day over baseline and feeling well	Symptomatic management: fluids, loperamide, avoid high fiber/lactose diet.<br>If Grade 1 persists for > 14 days manage as a Grade 2 event	Continue study treatment.
	**2**<br>Moderate symptoms: 4-6 liquid stools per day over baseline, or abdominal pain, or blood in stool, or nausea, or nocturnal episodes	Oral prednisolone 0.5 mg/kg/day (non-enteric coated).<br>Do not wait for any diagnostic tests to start treatment. Taper steroids over 2-4 weeks, consider endoscopy if symptoms are recurring.	Hold study treatment; resume when resolved/improved to baseline grade.
	**3**<br>Severe symptoms: > 6 liquid stools per day over baseline, or if episodic within 1 hour of eating	Initiate IV methylprednisolone 1-2 mg/kg/day.<br>Convert to oral prednisolone and taper over at least 4 weeks. Consider prophylaxis for adverse steroid effects, eg, blood glucose monitoring, vitamin D/calcium supplement.<br>If no improvement in 72 hours or symptoms worsen, consider infliximab 5 mg/kg if no perforation, sepsis, TB, hepatitis, NYHA Class III/IV CHF or other immunosuppressive treatment: MMF or tacrolimus.<br>Consult gastroenterologist to conduct colonoscopy/ sigmoidoscopy.	Hold study treatment; retreatment may be considered when resolved/improved to baseline grade and after discussion with the study medical monitor.
	**4**<br>Life-threatening symptoms		Discontinue study treatment.
**Skin reactions**	**1**<br>Skin rash, with or without symptoms, < 10% BSA	Avoid skin irritants and sun exposure; topical emollients recommended.	Continue study treatment.

Autoimmune Toxicity	Grade	Treatment Guidelines (Subject to Clinical Judgement)	Study Drug Management
	**2**<br>Rash covers 10%-30% of BSA	Avoid skin irritants and sun exposure; topical emollients recommended.<br>Topical steroids (moderate strength cream once a day or potent cream twice a day) ± oral or topical antihistamines for itch. Consider a short course of oral steroids.	Continue study treatment.
	**3**<br>Rash covers > 30% BSA or Grade 2 with substantial symptoms	Avoid skin irritants and sun exposure; topical emollients recommended.<br>Initiate steroids as follows based on clinical judgement:<br>For moderate symptoms: oral prednisolone 0.5-1 mg/kg/day for 3 days then taper over 2-4 weeks.<br>For severe symptoms: IV methylprednisolone 0.5-1 mg/kg/day; convert to oral prednisolone and taper over at least 4 weeks.	Hold study treatment.<br>Retreat when AE is resolved or improved to mild rash (Grade 1-2) after discussion with the study medical monitor.
	**4**<br>Skin sloughing > 30% BSA with associated symptoms (eg, erythema, purpura, epidermal detachment)	Initiate IV methylprednisolone 1-2 mg/kg/day. Convert to oral prednisolone and taper over at least 4 weeks.<br>Admit to a hospital and seek urgent dermatology consultation.	Discontinue study treatment.
**Hepatitis**	**1**<br>ALT or AST > ULN to 3X ULN	Check LFTs within 1 week and before the next dose check LFTs to verify that there has been no worsening.<br>If LFTs are worsening, recheck every 48-72 hours until improvement is seen.	Continue study treatment if LFTs are unchanged or improving.<br>Hold study treatment if LFTs are worsening until improvement is seen.
	**2**<br>ALT or AST 3-5X ULN	Recheck LFTs every 48-72 hours:<br>For persistent ALT/AST elevation: consider oral prednisolone 0.5-1 mg/kg/day for 3 days then taper over 2-4 weeks.<br>For rising ALT/AST: start oral prednisolone 1 mg/kg/day and taper over 2-4 weeks; re-escalate dose if LFTs worsen, depending on clinical judgement.	Hold study treatment, treatment may be resumed when resolved/improved to baseline grade and prednisolone tapered to ≤ 10 mg.

Autoimmune Toxicity	Grade	Treatment Guidelines (Subject to Clinical Judgement)	Study Drug Management
	**3**<br>ALT or AST 5-20X ULN	ALT/AST < 400 IU/L and normal bilirubin/INR/albumin: Initiate oral prednisolone 1 mg/kg and taper over at least 4 weeks.<br>ALT/AST > 400 IU/L or raised bilirubin/INR/low albumin: Initiate IV (methyl)prednisolone 2 mg/kg/day. When LFTs improve to Grade 2 or lower, convert to oral prednisolone and taper over at least 4 weeks.	Hold study treatment until improved to baseline grade; reintroduce only after discussion with the study medical monitor.
	**4**<br>ALT or AST > 20X ULN	Initiate IV methylprednisolone 2 mg/kg/day. Convert to oral prednisolone and taper over at least 6 weeks.	Discontinue study treatment.
	**Worsening LFTs despite steroids:**<br>• If on oral prednisolone change to pulsed IV methylprednisolone<br>• If on IV add mycophenolate mofetil (MMF) 500-1000 mg twice a day<br>• If worsens on MMF, consider addition of tacrolimus<br>Duration and dose of steroid required will depend on severity of event		
**Nephritis**	**1**<br>Creatinine 1.5X baseline<br>or > ULN to 1.5X ULN	Repeat creatinine weekly.<br>If symptoms worsen, manage as per criteria below.	Continue study treatment.
	**2**<br>Creatinine > 1.5-3X baseline or > 1.5-3X ULN	Ensure hydration and review creatinine in 48-72 hours; if not improving, consider creatinine clearance measurement by 24-hour urine collection. Discuss with nephrologist the need for kidney biopsy.<br>If attributed to study drug, initiate oral prednisolone 0.5-1 mg/kg and taper over at least 2 weeks.<br>Repeat creatinine/U&E every 48-72 hours.	Hold study treatment.<br>If not attributed to drug toxicity, restart treatment.<br>If attributed to study drug and resolved/improved to baseline grade: Restart study drug if tapered to < 10 mg prednisolone.
	**3**<br>Creatinine > 3X baseline or > 3-6X ULN	Hospitalize patient for monitoring and fluid balance; repeat creatinine every 24 hours; refer to a nephrologist and discuss need for biopsy. If worsening, initiate IV (methyl)prednisolone 1-2 mg/kg. Taper corticosteroids over at least 4 weeks.	Hold study treatment until the cause is investigated.<br>If study drug suspected: Discontinue study treatment.

Autoimmune Toxicity	Grade	Treatment Guidelines (Subject to Clinical Judgement)	Study Drug Management
	**4**<br>Creatinine > 6X ULN	As per Grade 3, patient should be managed in a hospital where renal replacement therapy is available.	Discontinue study treatment.
**Diabetes/ Hyperglycemia**	**1**<br>Fasting glucose value ULN to 160 mg/dL; ULN to 8.9 mmol/L	Monitor closely and treat according to local guideline. Check for C-peptide and antibodies against glutamic acid decarboxylase and islet cells are recommended	Continue study treatment.
	**2**<br>Fasting glucose value 160-250 mg/dL; 8.9-13.9 mmol/L	Obtain a repeat blood glucose level at least every week. Manage according to local guideline.	Continue study treatment or hold treatment if hyperglycemia is worsening. Resume treatment when blood glucose is stabilized at baseline or Grade 0-1.
	**3**<br>Fasting glucose value 250-500 mg/dL; 13.9-27.8 mmol/L	Admit patient to hospital and refer to a diabetologist for hyperglycemia management. Corticosteroids may exacerbate hyperglycemia and should be avoided.	Hold study treatment until patient is hyperglycemia symptom-free, and blood glucose has been stabilized at baseline or Grade 0-1.
	**4**<br>Fasting glucose value > 500 mg/dL; > 27.8 mmol/L	Admit patient to hospital and institute local emergency diabetes management. Refer the patient to a diabetologist for insulin maintenance and monitoring.	
**Ocular Toxicity**	**1**<br>Asymptomatic eye examination/test abnormality	Consider alternative causes and prescribe topical treatment as required.	Continue study treatment.
	**2**<br>Anterior uveitis or mild symptoms	Refer patient to an ophthalmologist for assessment and topical corticosteroid treatment. Consider a course of oral steroids.	Continue study treatment or hold treatment if symptoms worsen or if there are symptoms of visual disturbance.
	**3**<br>Posterior uveitis/ panuveitis or significant symptoms	Refer patient urgently to an ophthalmologist. Initiate oral prednisolone 1-2 mg/kg and taper over at least 4 weeks.	Hold study treatment until improved to Grade 0-1; reintroduce only after discussion with the study medical monitor.
	**4**<br>Blindness (at least 20/200) in the affected eyes	Initiate IV (methyl)prednisolone 2 mg/kg/day. Convert to oral prednisolone and taper over at least 4 weeks.	Discontinue study treatment
**Pancreatitis**	**2**<br>Asymptomatic, blood test abnormalities	Monitor pancreatic enzymes.	Continue study treatment

Autoimmune Toxicity	Grade	Treatment Guidelines (Subject to Clinical Judgement)	Study Drug Management
	**3**<br>Abdominal pain, nausea and vomiting	Admit to hospital for urgent management. Initiate IV (methyl)prednisolone 1-2 mg/kg/day. Convert to oral prednisolone when amylase/lipase improved to Grade 2, and taper over at least 4 weeks	Hold study treatment; reintroduce only after discussion with the study medical monitor.
	**4**<br>Acute abdominal pain, surgical emergency	Admit to hospital for emergency management and appropriate referral.	Discontinue study treatment.
**Arthritis**	**1**<br>Mild pain with inflammation, swelling	Management per local guideline.	Continue study treatment.
	**2**<br>Moderate pain with inflammation, swelling, limited instrumental (fine motor) activities	Management as per local guideline. Consider referring patient to a rheumatologist. If symptoms worsen on treatment manage as a Grade 3 event.	Continue treatment or, if symptoms continue worsens, hold study treatment until symptoms improve to baseline or Grade 0-1.
	**3**<br>Severe pain with inflammation or permanent joint damage, daily living activity limited	Refer patient urgently to a rheumatologist for assessment and management. Initiate oral prednisolone 0.5-1 mg/kg and taper over at least 4 weeks.	Hold study treatment unless improved to Grade 0-1; reintroduce only after discussion with the study medical monitor.
**Mucositis/ stomatitis**	**1**<br>Test findings only or minimal symptoms	Consider topical treatment or analgesia as per local guideline.	Continue study treatment.
	**2**<br>Moderate pain, reduced oral intake, limited instrumental activities	As per local guidelines, treat with analgesics, topical treatments and oral hygiene care. Ensure adequate hydration. If symptoms worsen or there is sepsis or bleeding, manage as a Grade 3 event.	Continue study treatment
	**3**<br>Severe pain, limited food and fluid intake, daily living activity limited	Admit to hospital for appropriate management. Initiate IV (methyl)prednisolone 1-2 mg/kg/day. Convert to oral prednisolone when symptoms improved to Grade 2 and taper over at least 4 weeks	Hold study treatment until improved to Grade 0-1.
	**4**<br>Life-threatening complications or dehydration	Admit to hospital for emergency care. Consider IV corticosteroids if not contraindicated by infection	Discontinue study treatment

Autoimmune Toxicity	Grade	Treatment Guidelines (Subject to Clinical Judgement)	Study Drug Management
**Myositis/ Rhabdomyolysis**	**1** Mild weakness with/without pain	Prescribe analgesics. If CK is significantly elevated and patient has symptoms, consider oral steroids and treat as Grade 2	Continue study treatment.
	**2** Moderate weakness with/without pain	If CK is 3 X ULN or worse initiate oral prednisolone 0.5-1 mg/kg and taper over at least 4 weeks.	Hold study treatment until improved to Grade 0-1.
	**3-4** Severe weakness, limiting self-care	Admit to hospital and initiate oral prednisolone 1 mg/kg. Consider bolus IV (methyl)prednisolone and1-2 mg/kg/day maintenance for severe activity restriction or dysphagia. If symptoms do not improve add immunosuppressant therapy. Taper oral steroids over at least 4 weeks.	Hold study treatment until improved to Grade 0-1. Discontinue if any evidence of myocardial involvement.
**Myocarditis**	**<2** Asymptomatic but significantly increased CK-MB, or increased troponin or clinically significant intraventricular conduction delay	Initiate cardiac evaluation under close monitoring with repeat serum testing and including ECG, cardiac echo/MUGA, and/or other interventions per institutional guidelines; consider referral to a cardiologist. If diagnosis of myocarditis is confirmed, treat as Grade 2	Hold study treatment. If a diagnosis of myocarditis is confirmed and considered immune-related, permanently discontinue study treatment in patients with moderate or severe symptoms. Patients with no symptoms or mild symptoms may not restart tislelizumab unless cardiac parameters have returned to baseline and after discussion with the study medical monitor.
	**2** Symptoms on mild-moderate exertion	Admit to hospital and initiate oral prednisolone or IV (methyl)prednisolone at 1-2 mg/kg/day. Consult with a cardiologist and manage symptoms of cardiac failure according to local guidelines. If no immediate response change to pulsed doses of (methyl)prednisolone 1g/day and add MMF, infliximab or anti-thymocyte globulin	
	**3** Severe symptoms with mild exertion		
	**4** Life-threatening		

Abbreviations: AE, adverse event; ALT, alanine aminotransferase; AST, aspartate aminotransferase; BSA, body surface area; CHF, congestive heart failure; CK, creatine kinase; CK-MB, creatine kinase cardiac muscle isoenzyme; ECG, electrocardiogram; INR, international normalized ratio; IV, intravenous; LFT, liver function test; MMF, mycophenolate mofetil; MUGA, multigated acquisition scan; NYHA, New York Heart Association; T4, thyroxine; TB, tuberculosis; TFT, thyroid function test; TSH, thyroid-stimulating hormone; U&E, urea and electrolytes; ULN, upper limit of normal

APPENDIX 11. LIST OF CHINESE HERBAL MEDICINE OR CHINESE PATENT MEDICINES WHICH HAVE EFFECT OF CONTROL CANCER OR BOOST IMMUNITY

The following table lists those medications that require a 14-day wash-out and should be prohibited during the study:

Drug Name (Chinese)	Drug Name (English)
Rg3 参一胶囊	Ginsenoside-Rg3 capsule
养正消积胶囊	Yangzheng Xiaoji Jiaonang
化癥回生口服液	Huazheng Huisheng Koufuye
十全大补汤	Juzentaihoto
华蟾素注射液	Cinobufacini/Huachansu injection
华蟾素片/胶囊	Cinobufacini/Huachansu Pian/Capsules
博尔宁胶囊	Boerning capsule
去甲斑蝥素片	Norcantharidin Pian
参丹散结胶囊	Shendan Sanjie Jiaonang
参芪扶正注射液	Shengqi Fuzheng Zhusheye
参莲胶囊/颗粒	Shen Lian Jiao Nang/Ke Li
吗特灵注射液	Ma Te Ling injection
回生口服液	Hui Sheng Kou Fu Ye
复方斑蝥胶囊	Fufang Banmao Jiaonang
复方红豆杉胶囊	Fufang Hongdoushan Jiaonang
复方苦参注射液	Fufang Kushen Zhusheye
天仙胶囊	Tian Xian capsule
奇宁注射液	Qining injection
威麦宁胶囊	Weimaining Jiao Nang
安尔欣注射液	Anerxin/Ginseng polysaccharide injection
安康欣胶囊	Ankangxin Jiaonang
安替可胶囊	Antike capsule
岩舒注射液	Yanshu injection
平消片/胶囊	Ping Xiao Pian/Jiao Nang
康力欣胶囊	Kanglixin Jiaonang

Drug Name (Chinese)	Drug Name (English)
康艾注射液	Kang'ai Zhusheye
康莱特注射液	Kanglaite Injection
康莱特软胶囊	Kanglaite Soft Capsules
慈丹胶囊	CIDAN Capsule
槐耳颗粒	Huaer Keli
海生素注射液	Haishengsu injection
消癌平丸/片/胶囊/颗粒	Xiaoaiping Wan/Pian/Jiao Nang/Ke Li
消癌平注射液	Xiaoaiping Zhusheye
牛黄醒消丸	Niuhuang Xingxiao pill
猪苓多糖注射液	Polyporus polysaccharide injection
白花蛇舌草注射液	Hedyotis Dissusa wild injection
紫龙金片	Zi Long jin pian
肝复乐片/胶囊	Ganfule Jiaonang / GFL tablet
肿节风片	Zhongjiefeng tablet
胃复春片	Weifuchun pill
艾迪注射液	Ai Di Zhu She Ye
芪珍胶囊	Qizhen Jiaonang
莪术油注射液	Zedoary turmeric oil injection
金复康口服液	Kanglixin Jiaonang
金蒲胶囊	Jinpu capsule
金龙胶囊	Jinlong Capsules
香菇多糖	Lentinan
鸦胆子油乳注射液	Yadanzi/Brucea javanica Youru Zhusheye
鸦胆子油软胶囊/口服乳液	Yadanziyou Ruan jiao nang/Kou Fu Ru Ye

Terminology list: Pian = tablet, Jiao Nang/Jiaonang = capsule, Ke Li/Keli = granules, Zhue She ye/Zhusheye = injections, Kou Fu Ye/koufuye = oral liguid, Wan = Pill/bolus, He Ji/Heji = mixture, Gao = ointment
NOTE: These list of Chinese herbal medicines or Chinese patent medicines are provided as examples and are not intended to be all-inclusive.

APPENDIX 12. LIST OF COUNTRY SPECIFIC PROTOCOL AMENDMENTS

Protocol Amendment 3.1 Date: 18 December 2017 (Japan)

Protocol Amendment 3.2 Date: 19 December 2017 (United Kingdom)

Protocol Amendment 3.3 Date: 29 March 2018 (France)

Protocol Amendment 3.4 Date: 04 April 2018 (Germany)

Protocol Amendment 4.1 03 July 2018 (France)

Protocol Amendment 4.2 05 July 2018 (United Kingdom)

APPENDIX 13. SAFETY RUN-IN SUBSTUDY INVESTIGATING SAFETY, TOLERABILITY, PHARMACOKINETICS AND PRELIMINARY ANTITUMOR ACTIVITY OF ANTI-PD-1 MONOCLONAL ANTIBODY TISLELIZUMAB IN JAPANESE PATIENTS WITH UNRESECTABLE HEPATOCELLULAR CARCINOMA

SYNOPSIS FOR SUBSTUDY

**Name of Sponsor: BeiGene, Ltd.**
**Investigational Product: tislelizumab**
**Title of Study:** Safety Run-in Substudy Investigating Safety, Tolerability, Pharmacokinetics and Preliminary Antitumor Activity of Anti-PD-1 Monoclonal Antibody tislelizumab in Japanese Patients with Unresectable Hepatocellular Carcinoma
**Protocol Identifier: BGB-A317-301 Substudy**
**Number of Patients:** Six patients will be enrolled firstly to assess dose-limiting toxicity (DLT), with enrollment of approximately 10 patients to adequately assess pharmacokinetic (PK) profile. (Up to 20 patients will be enrolled if more than 1 dose level is explored.)
**Study Centers:** To be determined
**Study Objectives:**<br>Primary:<br>• To assess the safety and tolerability of tislelizumab in Japanese patients with hepatocellular carcinoma (HCC)<br>• To confirm the pivotal Phase 3 dose of tislelizumab in Japanese patients<br>• To characterize the pharmacokinetics of tislelizumab in Japanese patients<br>Secondary:<br>• To assess the preliminary antitumor activity of tislelizumab<br>• To assess host immunogenicity to tislelizumab<br>Exploratory:<br>• To explore correlations between drug exposure and response
**Study Endpoints:**<br>Primary:<br>• Tislelizumab safety and tolerability: The safety of tislelizumab will be assessed throughout the study by monitoring adverse events (AEs) and serious adverse events (SAEs) per National Cancer Institute Common Terminology Criteria for Adverse Events (NCI-CTCAE) Version (v)4.03, relevant physical examination, electrocardiograms (ECGs) and laboratory assessments as needed<br>• The pivotal Phase 3 dose for tislelizumab will be confirmed based on safety, tolerability, and other available data<br>• Pharmacokinetic (PK) evaluations: Individual tislelizumab concentrations and PK parameters will be tabulated by visit/cycle and/or dose cohort<br>Secondary:<br>• Efficacy evaluations: Objective response rate (ORR), progression-free survival (PFS), and duration of response (DOR) will be determined by investigators based on Response Evaluation Criteria in Solid Tumors (RECIST) Version (v)1.1. Overall survival (OS) will be evaluated.<br>• Anti-tislelizumab antibody: Assessments of immunogenicity of tislelizumab to determine the incidence of antidrug antibodies (ADAs)<br>Exploratory:<br>• Assessments of the correlations between drug exposure and response (efficacy, safety end points)

Study Assessments:

Dose-limiting toxicities will be assessed among evaluable patients after 28 days on study. An evaluable patient is defined as the patient who has received at least 80% of the dose and completed all safety assessments required during the first 28 days, or any patient who has experienced a DLT within 28 days. The first 6 patients will be hospitalized for the first week of Cycle 1.

Tumor response will be evaluated by the investigator every 9 weeks during Year 1 and every 12 weeks from Year 2 onwards, in accordance with RECIST v1.1. If a patient discontinues study treatment due to any reason other than disease progression or death, tumor assessments will continue as scheduled until, disease progression, death, loss to follow-up, or withdrawal of consent, whichever occurs first.

Patients will be evaluated for any AEs and serious adverse events (SAEs) occurring up to 30 days after the last dose of study drug (all severity grades, per NCI-CTCAE v4.03) or initiation of new anticancer therapy, whichever occurs first, and immune-related AEs (irAEs) occurring up to 90 days after the last dose of study drug, regardless of whether or not the patient starts a new anticancer therapy. All study drug-related SAEs will be recorded by the investigator after treatment discontinuation until patient death or loss to follow-up, whichever occurs first.

Safety and efficacy monitoring will be performed by an Independent Data Monitoring Committee (IDMC) as per the main protocol (Section 10.2). The IDMC will additionally evaluate and confirm the Phase 3 dose based on the safety and tolerability of tislelizumab, and decide whether or not to add unscheduled dose levels for trial. The IDMC may recommend modifications to the study, including termination due to safety and/or efficacy concerns.

Dose Level

Based on the safety results of the Phase 1 trials of tislelizumab (Studies BGB-A317-001 and BGB-A317-102), this study will initially assess the safety and PK characteristics of fixed dose 200 mg once every 3 weeks (Q3W) in Japanese patients with unresectable HCC. Other dose levels may be further explored based on the safety result.

Among the 6 patients in the 200-mg Q3W cohort, if 2 or more patients experience DLT in the first 28 days on treatment, such starting dose will be considered as exceeding the maximum tolerated dose (MTD), and a lower dose, such as 150 mg Q3W, will be assessed subsequently in 3-6 patients.

If the 200-mg Q3W regimen passes the DLT assessment the cohort at such dose level can be expanded to approximately 10 patients to further assess the safety, tolerability, pharmacokinetics and preliminary pharmacodynamic characteristics of tislelizumab. Up to 20 patients may be enrolled if a lower dose level is evaluated (such as 150 mg Q3W).

Dose-limiting Toxicity

All toxicities or AEs will be graded according to the NCI-CTCAE v4.03. The occurrence of any of the following toxicities during the first 28 days on treatment will be considered DLT, if judged by the investigator as related to study drug administration:

Hematologic Dose-limiting Toxicities:

1. Grade 4 neutropenia lasting > 7 days
2. Febrile neutropenia (defined as absolute neutrophil count [ANC] < 1000/mm^3 with a single temperature of 38.3°C or a sustained temperature of 38°C for > 1 hour)
3. Grade 3 neutropenia with infection
4. Grade 3 thrombocytopenia with bleeding or transfusion requirement
5. Grade 4 thrombocytopenia with or without transfusion
6. Grade 4 anemia (life-threatening) or Grade 3 anemia requiring transfusion

Non-hematologic Dose-limiting Toxicities:

1. Grade 4 or above toxicity
2. Grade 3 toxicity lasting more than 7 days despite optimal supportive care

Note: The following AEs will not be considered as DLTs:

- Grade 3 endocrinopathy that is adequately controlled by hormonal replacement
- Grade 3 AE of tumor flare (defined as local pain, irritation, or rash localized at sites of known or suspected tumors)
- Grade 3 rash
- Grade 3 to Grade 4 laboratory abnormalities that are not associated with clinical sequelae (eg, LDH)

In addition, AEs leading to temporary or permanent discontinuation of tislelizumab dosing, with the exception of infusion-related reactions should be considered a DLT and any clinically important or persistent toxicities (including infusion-related reactions) that are not included above may also be considered a DLT following review by the IDMC.

Patients who received <80% of the assigned dose of tislelizumab (eg, because the infusion had to be discontinued due to an infusion reaction) and did not experience a DLT will not be taken into account in the assessment of the overall DLT rate for the particular dose level.

Key Eligibility Criteria:

The population under study is adult Japanese patients (≥ 20 years of age on the day the patient voluntarily agrees to participate in the study) with histologically confirmed HCC that is classified as Barcelona Clinic Liver Cancer (BCLC) Stage C disease or BCLC Stage B disease that has been previously treated with standard of care treatment or for whom standard treatment is not available, not tolerated, or refused. All patients are also required to have a Child-Pugh A classification assessed within 7 days before enrollment, ≥ 1 measurable lesion per RECIST v1.1, and an Eastern Cooperative Oncology Group (ECOG) Performance Status (PS) score of ≤ 1.

Test Product, Dose, and Mode of Administration:

Tislelizumab will be administered at a dose of 200 mg intravenously (IV) Q3W. The proposed dose level can be further adjusted according to the safety, and tolerability observed in the dose verification stage, and a lower dose level may be added if necessary.

Statistical Methods:

Descriptive statistics will be used to summarize the demographic, disease characteristic, efficacy and safety data. No statistical hypotheses are planned in this exploratory study.

All patients who have received tislelizumab will be included in the Safety analysis set. All patients with valid tislelizumab PK sampling after treatment will be included in the PK analysis set. For other parameters, all evaluable data will be included in the summaries.

Efficacy Analysis:

The efficacy per RECIST 1.1 (ie, ORR, PFS and DOR) will be summarized to explore the preliminary anticancer activities in Japanese patients.

The ORR is defined as the proportion of patients who had confirmed complete response (CR) or partial response (PR) assessed by the investigator using RECIST v1.1.

The DOR is defined as the time from the first determination of a confirmed objective response by investigator according to RECIST v1.1 until the first documentation of progression or death, whichever comes first.

The PFS is defined as the time from the date of first dose of study drug to the date of first documentation of disease progression assessed by investigator using RECIST v1.1 or death, whichever occurs first.

The ORR and its 95% confidence interval (CI) will be summarized in the Safety analysis set. PFS and DOR will be estimated using the Kaplan-Meier method. Waterfall plot of maximum tumor shrinkage

per patient will be presented.

Safety Analysis:

Safety will be determined by the reporting of AEs and by laboratory values (hematology, clinical chemistry, coagulation, and urinalysis). Vital signs, physical examinations, and ECG findings will also be used in determining safety. The severity of AEs will be graded according to the NCI-CTCAE v4.03. The incidence of DLT events and treatment-emergent adverse events (TEAEs) will be reported as the number (percentage) of patients with TEAEs by System Organ Class and Preferred Term. Descriptive summary statistics (eg, n, mean, standard deviation, median, minimum, maximum for continuous variables; n [%] for categorical variables) and changes from baseline will be determined for laboratory parameters and vital signs.

Statistical methods will be described in detail in the Statistical Analysis Plan.

1. INTRODUCTION

Before initiating this Phase 3 study in Japan, a substudy investigating the safety, tolerability, pharmacokinetics (PK), and preliminary efficacy is planned. This study is a dose-validation clinical study of the monoclonal antibody tislelizumab in Japanese patients with previously treated, unresectable hepatocellular carcinoma (HCC), aiming to explore safety, tolerability, and pharmacokinetics (PK). This study is carried out on the basis of Phase 1 studies (Studies BGB-A317_001 and BGB-A317-102). According to the preliminary results of the Phase 1A Study BGB-A317_001, 0.5, 2, 5 and 10 mg/kg once every 2 weeks (Q2W) are all tolerable doses. In addition, 2 and 5 mg/kg once every 3 weeks (Q3W) and 200 mg Q3W have also been confirmed as tolerable. A 200-mg Q3W fixed dose was selected as the pivotal dose. In a Phase 1 clinical study in China (BGB-A317-102), 6 patients have been administered the 200-mg Q3W fixed dose, which PK analysis indicated to be equivalent to 2.4 – 3.8 mg/kg based on body weight. The comparison results of dose normalized maximum observed plasma concentration (C_{max}) and area under the concentration-time curve from Day 0 to Day 14 (AUC_{0-14d}) showed that the PK is consistent in Chinese and Caucasian patients. The available safety and PK data suggests that the proposed Phase 1 dose of 200 mg Q3W tislelizumab is expected to be a safe dose to investigate in Japanese patients.

The background on hepatocellular carcinoma (HCC) and on the monoclonal antibody, tislelizumab, is provided in the main study, Section 1, Introduction.

2. STUDY OBJECTIVES AND ENDPOINTS

2.1. Objectives

2.1.1. Primary Objectives

- To assess the safety and tolerability of tislelizumab in Japanese patients with unresectable HCC who have received prior standard of care treatment
- To confirm the pivotal Phase 3 dose of tislelizumab in Japanese patients
- To characterize the pharmacokinetics of tislelizumab in Japanese patients

2.1.2. Secondary Objectives:

- To assess the preliminary antitumor activity of tislelizumab
- To assess host immunogenicity to tislelizumab

2.1.3. Exploratory Objectives

- To explore correlations between drug exposure and response

2.2. Endpoints

2.2.1. Primary Endpoints

- Tislelizumab safety and tolerability: The safety of tislelizumab will be assessed throughout the study by monitoring adverse events (AEs) and serious adverse events (SAEs) per National Cancer Institute Common Terminology Criteria for Adverse Events (NCI-CTCAE Version [v]4.03), relevant physical examination, electrocardiograms (ECGs) and laboratory assessments as needed
- The pivotal Phase 3 dose for tislelizumab will be determined based on safety and tolerability, and other available data (which may include pharmacokinetics, preliminary efficacy)
- Pharmacokinetic evaluations: Individual tislelizumab concentrations and PK parameters will be tabulated by visit/cycle and/or dose cohort

2.2.2. Secondary Endpoints

- Efficacy evaluations: Objective response rate (ORR), progression-free survival (PFS), and duration of response (DOR) will be determined by investigators based on Response Evaluation Criteria in Solid Tumors (RECIST) Version (v)1.1. Overall survival (OS) will be evaluated.
- Anti-tislelizumab antibody: Assessments of immunogenicity of tislelizumab to determine the incidence of antidrug antibodies (ADAs)

2.2.3. Exploratory Endpoints

- Assessments of the correlations between drug exposure and response (efficacy, safety end points)

3. STUDY DESIGN

3.1. Summary of Study Design

This is an open-label, multicenter, non-randomized Phase 1 clinical trial in Japanese patients with unresectable HCC who have previously received standard of care treatment. Screening can last up to 28 days and treatment can continue until the investigator considers the patient is no longer benefiting from tislelizumab, toxicity, or voluntary withdrawal of study treatment. Six patients will be enrolled to assess dose-limiting toxicities (DLTs), with enrollment of approximately 10 patients to adequately address the PK profile of tislelizumab in Japanese patients. Up to 20 patients may be enrolled if more than 1 dose level is explored. A Safety Follow-up phase will occur up to 30 days following last study treatment or initiation of new cancer therapy, whichever occurs first, for any AEs, and up to 90 days following last dose of tislelizumab for irAEs, regardless of whether or not the patient starts a new anticancer therapy. Survival follow-up information will be collected approximately every 3 months (± 14 days) after the Safety Follow-up Visit until death, loss to follow-up, withdrawal of consent, or study

termination by sponsor. The first 6 patients will remain hospitalized during the first week of the first cycle of treatment.

3.2. Schedule of Study Assessments

A schedule of efficacy and safety assessments is presented in Appendix 13 Table 1.

Appendix 13 Table 1: Japan Substudy Schedule of Assessments

Assessment	Screening[1]	Treatment Cycles					Safety Follow-up[3]	Survival Follow-up[4]
		Cycles 1 to 3			Cycle 4 and Subsequent Cycles (Every 21 Days)	End of Treatment Visit[2]		
**Days (Window)**	**-28 to ~ -1**	**1 (±3 for C2 and C3)**	**8 (±2)**	**15 (± 2)**	**1 (± 3)**	**0 to 7 Days**	**30 ± 7 Days After Last Dose**	**Every 3 Months (± 14 days)**
Informed consent	x							
Inclusion/exclusion criteria	x							
Demographics/ Medical history/ Prior medications[5]	x							
Child-Pugh classification score	x[6]							
Vital signs/ Height and Weight[7]	x	x			x	X	x	
Physical examination[24]	x	x			x	X	x	
ECOG Performance Status	x	x			x	X	x	
12-lead ECG[7]	x	As clinically indicated					x	
Adverse events[8]	x	x	x[25]	x[25]	x	X	x	x
Concomitant medications	x	x	x[25]	x[25]	x	X	x	
Hematology[9]	x[1]	x	x	x	x	x[2]	x	
Serum chemistry[9]	x[1]	x	x	x	x	x[2]	x	
Coagulation parameters[9, 10]	x[1]	x			x	x[2]	x	
Urinalysis[9]	x	As clinically indicated						
Pregnancy test[11]	x	x			x			

Assessment	Screening[1]	Treatment Cycles					Safety Follow-up[3]	Survival Follow-up[4]
		Cycles 1 to 3			Cycle 4 and Subsequent Cycles (Every 21 Days)	End of Treatment Visit[2]		
Days (Window)	-28 to ~ -1	1 (±3 for C2 and C3)	8 (±2)	15 (± 2)	1 (± 3)	0 to 7 Days	30 ± 7 Days After Last Dose	Every 3 Months (± 14 days)
Thyroid function[12]	x[1]				x		x	
Pulmonary function tests[13]	x							
Pharmacokinetics[14]		x			x		x	
Anti-Tislelizumab antibodies[15]		x			x		x	
HBV/HCV tests[16]	x	As clinically indicated						
AFP[9]	x	x			x	x		
Tumor assessment[17]	x				x	x[2]		x
Archival tumor tissue [18]	x							
Fresh tumor tissues (optional)[19]	x							
Tislelizumab administration[20]		x			x			
EQ-5D-5L[21]	x	x			x	x		
EORTC QLQ-C30[21]	x	x			x	x		
EORTC QLQ-HCC18[21]	x	x			x	x		
Survival status								x
Optical coherence tomography (or equivalent diagnostic test) and visual acuity tests[22]	x				x	x[23]	x[23]	

Assessment	Screening[1]	Treatment Cycles					Safety Follow-up[3]	Survival Follow-up[4]
		Cycles 1 to 3			Cycle 4 and Subsequent Cycles (Every 21 Days)	End of Treatment Visit[2]		
**Days (Window)**	**-28 to ~ -1**	**1 (±3 for C2 and C3)**	**8 (±2)**	**15 (± 2)**	**1 (± 3)**	**0 to 7 Days**	**30 ± 7 Days After Last Dose**	**Every 3 Months (± 14 days)**
Digital pulse oximetry (to determine SpO_2)	x	x			x	x		

1. Written informed consent is required prior to performing any study-specific tests or procedures. Results of standard of care tests or examinations performed prior to obtaining informed consent and within 28 days prior to enrollment may be used for Screening assessments rather than repeating such tests.
2. The End of Treatment Visit is conducted when the investigator determines that tislelizumab will no longer be used. If routine laboratory tests (eg, hematology and serum chemistry) are completed within 7 days before the End of Treatment Visit, tests need not be repeated. Tumor assessment is not required at the End of Treatment Visit provided that fewer than 6 weeks have passed since the last assessment.
3. The Safety Follow-up Visit is required to be conducted 30 days (± 7 days) after the last dose of tislelizumab or before the initiation of a new anticancer treatment, whichever occurs first. In the situation where study treatment is withheld/interrupted for 30 days or more prior to permanent discontinuation of study treatment (End of Treatment Visit), the Safety Follow-up Visit should be conducted at the same time as the End of Treatment Visit. Common procedures to both visits should be performed only once.
4. Survival Follow-up information will be collected via telephone calls, patient medical records, and/or clinic visits approximately every 3 months (± 14 days) after the Safety Follow-up Visit until death, loss to follow-up, withdrawal of consent, or study termination by sponsor. All patients will be followed for survival and subsequent anticancer therapy information unless a patient requests to be withdrawn from follow-up.
5. Includes age or date of birth, gender, and self-reported race/ethnicity; history of treatment for the primary diagnosis, including prior medication, loco-regional treatment(s), and surgical treatment(s). Information on radiographic studies performed prior to study entry may be collected for review by the investigator.
6. Patients are required to have Child-Pugh A classification for liver function assessed within 7 days of enrollment.
7. Vital signs include temperature, pulse rate, and blood pressure (systolic and diastolic) while the patient is in a seated position after resting for 10 minutes. The patient's vital signs are required to be recorded within 60 minutes before; during; and 30 minutes after the first infusion of tislelizumab. For subsequent infusions, vital signs will be collected within 60 minutes before infusion and if clinically indicated, during and 30 minutes (up to + 15 minutes) after the infusion.
   ECG recordings will be obtained during Screening, the Safety Follow-up Visit, and as clinically indicated at other time points. Patients should be resting for at least 10 minutes prior to each ECG collection.
8. The AEs and laboratory abnormalities will be graded per NCI-CTCAE v4.03. All AEs will also be evaluated for seriousness. After the informed consent form has been signed, but prior to the administration of study drug, only SAEs should be reported. After the first dose of study drug, all AEs and SAEs, regardless of relationship to study drug, will be reported until either 30 days after the last dose of study treatment or the initiation of new anticancer therapy, whichever occurs first. Immune-related AEs (serious and nonserious) will be reported for 90 days after the last dose of tislelizumab, regardless of whether or not the patient starts a new anticancer therapy. All drug-related SAEs will be recorded by the investigator after treatment discontinuation until patient death or loss to follow-up, whichever occurs first.

After a patient has been discontinued from the study drug(s), investigators are not obligated to actively seek AEs or SAEs in former patients. However, if the investigator learns of any SAE, including a death, and he/she considers the SAE related to the study drug, the investigator will notify the sponsor as described in Table 5 in Section 8.7.2.1 of the main protocol.

9. Local or central laboratory assessments on serum chemistry, hematology, coagulation, and urinalysis will be conducted, of which certain elements will be collected as specified in Appendix 5. If laboratory tests at screening (hematology, chemistry, coagulation, urinalysis, and alpha fetoprotein) are not performed within 7 days prior to the administration of study drug on Cycle 1 Day 1, these tests should be repeated and reviewed before study drug administration. Patients with electrolyte deficiencies, including magnesium, potassium, and calcium, must have these clinically corrected in the screening period before enrollment in the study. Hematology and serum chemistry (including liver function tests) will be performed weekly through the end of the 28-day DLT assessment period. After the DLT assessment period, hematology and serum chemistry (including liver function tests) will be performed weekly for Cycle 2 and Cycle 3 and then at the beginning of subsequent cycles from Cycle 4 and beyond (data collected as specified in Appendix 5). After Cycle 1, results are to be reviewed within 48 hours before study drug administration. Urinalysis is to be conducted during the treatment period only if clinically warranted. Refer to Section 8.4.5 of the main protocol for additional information regarding clinical assessment and management of clinical laboratory abnormalities.
10. Includes international normalized ratio, prothrombin time, and activated partial thromboplastin time.
11. Urine or serum pregnancy test (for women of childbearing potential, including women who have had a tubal ligation) must be performed and documented as negative within 7 days prior to enrollment. If screening pregnancy test is > 7 days prior to enrollment, it must be repeated at Cycle 1 Day 1, prior to dosing. Urine pregnancy tests will be performed at each visit prior to dosing. A serum pregnancy test must be performed if the urine pregnancy test is positive or equivocal.
12. Analysis of FT3, FT4, and TSH will be performed by a central laboratory or local study site laboratory. Thyroid function tests will be performed at Screening and every 3 cycles (ie, Day 1 of Cycles 4, 7, 10, et cetera), and at the Safety Follow-up Visit.
13. Patients who are suspected or known to have serious/severe respiratory conditions or exhibit significant respiratory symptoms unrelated to the underlying cancer will have pulmonary function testing which may include but is not limited to spirometry and assessment of diffusion capacity done during the Screening period to assist the determination of suitability on the study. At least 50% of performance expected by age is required for spirometry (including FVC and FEV_1). Assessment of diffusion capacity during Screening is required in patients with a history of thoracic radiotherapy or restrictive lung disease. When DLCO is performed, at least 60% of performance expected by age is required.
14. Refer to Appendix 13 Table 2 for details on timing of pharmacokinetics sample collection. Procedures for collection of PK samples are described in the Lab Manual. All predose samples should be drawn at the same time as blood collection for anti-tislelizumab antibodies. Intensive PK at Cycle 1 and Cycle 5; Sparse PK at Cycle 2, 3, 7, 9, 13, 17, 25 and 33, and every 8 cycles thereafter. An additional PK sample is required to be collected at the Safety Follow-up Visit. Should a patient present with any ≥ Grade 3 immune-related AE, an additional blood PK sample may be taken to determine the serum concentration of tislelizumab.
15. Blood used to test for anti-tislelizumab antibodies should be collected within 60 minutes before beginning the Day 1 infusion of Cycles 1, 2, 3, 5, 7, 9, 13, 17, 25, 33, every 8 cycles thereafter, and at the mandatory Safety Follow-up. All samples should be drawn at the same time as blood collection for predose PK analysis.
16. Testing will be performed by a central laboratory and/or the local laboratory at Screening and will include HBV/HCV serology (HBsAg, HBsAb, HBcAb, and HCV antibody) and viral load assessment (HBV DNA and HCV RNA). Patients who have detectable HBV DNA or HCV RNA at Screening will perform the respective viral load test every 4 cycles (ie, Day 1 of Cycle 5, 9, 13, et cetera).
17. Radiological images captured as standard of care prior to obtaining written informed consent and within 28 days of enrollment may be used rather than repeating tests. All measurable and evaluable lesions are required to be assessed and documented at the Screening Visit. An MRI (or CT scan if MRI is contraindicated or not readily available) of the head may be required at screening based on clinical judgement; bone scan or ^{18}F-NaF PET is required if clinically indicated. The same radiographic procedure must be used throughout the study for each patient. The investigator must review radiograph results

before dosing at the following cycle. Patients will undergo tumor assessments every 9 weeks (± 7 days) during Year 1 and every 12 weeks (± 7 days) from Year 2 onwards (based on RECIST v1.1 assessment). The investigator may perform additional scans or more frequent assessments if clinically indicated. See Section 7.4 of the main protocol for more information.
Patients who discontinue study treatment early for reasons other than disease progression (eg, toxicity) will continue to undergo tumor assessments following the original plan until the patient experiences disease progression, withdraws consent, dies, or until the study terminates, whichever occurs first.
Patients who continue treatment with tislelizumab beyond radiographic disease progression will be monitored with a follow-up scan no more than 6 to 8 weeks beyond the initial diagnosis of radiographic PD before discontinuation of tislelizumab treatment

18. Patients are required to provide archival tumor tissues (FFPE blocks or approximately 10 [≥ 5] unstained slides) for biomarker analysis (except if not allowed by local regulations/IRBs/IECs).
19. Fresh biopsy: In the absence of archival tumor tissues, a fresh biopsy of a tumor lesion at baseline is optional (written informed consent is required prior to fresh tumor biopsies). See Section 7.6 of the main protocol for more information.
20. Tislelizumab will be given IV Q3W. The initial infusion (Cycle 1, Day 1) will be delivered over 60 minutes, and then can be administered over 30 minutes for subsequent infusions if well tolerated. Patients must be monitored for 2 hours after infusion of tislelizumab on Day 1 of Cycle 1 and Cycle 2, from Cycle 3 onward, at least a 30-minute monitoring period is required. The first 6 patients will remain hospitalized during the first week of the first cycle of treatment. The first dose will be given on Cycle 1 Day 1 and subsequent dosing will continue on the original 21-day treatment interval schedule. Each cycle has a (±) 3-day window, however in case of dose delays there should be a minimum of 14 days between tislelizumab dosing days.
21. To be completed prior to any clinical activities that are performed that day during on-study site visits. EQ5D-5L, EORTC QLQ-C30, and EORTC QLQ-HCC18 will be completed at Screening, and/or baseline, at every other cycle through Cycle 12, then every 4 cycles thereafter, and at EOT.
22. Eye exam, visual acuity test, and optical coherence tomography (or equivalent diagnostic test for retinal examination) captured as standard of care prior to obtaining written informed consent and within 28 days of randomization may be used rather than repeating tests. Eye exam, visual acuity test, and optical coherence tomography (or equivalent diagnostic test) will be assessed by an ophthalmologist at the Screening Visit. Patients will undergo repeat assessments approximately every 15 weeks (± 7 days)
23. The ophthalmologic assessments including eye exam, visual acuity test, and optical coherence tomography (or equivalent diagnostic test) should only be performed once at either the EOT or during safety follow-up within 30 days of study treatment end.
24. Investigators should solicit patients regarding changes in vision, visual disturbance, or ocular inflammation at each scheduled study visit during tislelizumab treatment. For any change in vision, referral to an ophthalmologist will be made for further management guidance.
25. Review of AEs and concomitant medications may be conducted by telephone on Days 8 and 15

Abbreviations: ADA, antidrug antibody; AE, adverse event; AFP, alpha fetoprotein; DLCO, diffusing capacity for carbon monoxide; DNA, deoxyribonucleic acid; ECG, electrocardiogram; ECOG, Eastern Cooperative Oncology Group; EORTC QLQ-HCC18, European Organisation for Research and Treatment of Cancer Quality of Life Questionnaire–Hepatocellular Carcinoma 18 Questions; EORTC QLQ-C30, European Organisation for Research and Treatment of Cancer Quality of Life Questionnaire-Core 30; EQ-5D-5L, 5-level European Quality of Life 5-Dimensions (health questionnaire); FEV_1, forced expiratory volume in the first second of expiration; FFPE, formalin-fixed paraffin-embedded; FVC, forced vital capacity; HBV, hepatitis B virus; HCV, hepatitis C virus; HBcAb, hepatitis B core antibody; HBsAb, hepatitis B surface antibody; HBsAg, hepatitis B surface antigen; irAE, immune-related adverse event; IV, intravenously; MRI, magnetic resonance imaging; ^{18}F-NaF PET, 18F-sodium fluoride position emission tomography; NCI-CTCAE, National Cancer Institute Common Terminology Criteria for Adverse Events; PK, pharmacokinetic(s); Q3W, once every 3 weeks; RECIST, Response Evaluation Criteria in Solid Tumors; SAE, serious adverse event; SpO_2, peripheral oxygen saturation RNA, ribonucleic acid; TEAE, treatment-emergent adverse event; TSH, thyroid stimulating hormone; v, version.

Appendix 13 Table 2: Schedule of Japan Substudy Pharmacokinetic and Anti-Tislelizumab Antibody Sampling

Japan Substudy		Days	Timing in relation to dose	Time Points	Anti-Tislelizumab Antibody Samples
Treatment Period	Cycle 1 (21 days)	1	Predose	-60 min to 0 h	X
			Postdose	End infusion to 30 min[1]	—
		2	--	24 h[2]	—
		4	--	72 h[2]	—
		8 ± 2	--	any time in the window	—
		15 ± 2	--	any time in the window	—
	Cycle 2 and additional cycles (Cycle 3, 7, 9, 13, 17, 25, 33, and every 8 cycles thereafter) (21 days) [3]	1 ± 2	Predose	-60 min to 0 h	X
			Postdose	End infusion to 30 min[1]	—
	Cycle 5 (21 days)	1 ± 2	Predose	-60 min to 0 h	X
			Postdose	End infusion to 30 min[1]	—
		2	--	24 h[2]	—
		4	--	72 h[2]	—
		8 ± 2	--	any time in the window	—
		15 ± 2	--	any time in the window	—
	Cycle 6	1 ± 2	Predose	-60 min to 0 h	—
Safety Follow-up	(30 days ± 7 days after last dose)	30 ± 7	--	any time in the window	X[4]

Please note: Actual drug dosing and PK sampling times must be documented by the sites and will be captured in the database.

1. Sample collection must be from opposite arm to that used for study drug infusion. If drug was administered via a central venous catheter, sample collection for PK should be from a different site.
2. Window: ±2 h.
3. Cycle 2, Cycle 3 and subsequently every 2 cycles except for Cycle 5 during the first 6 months of study therapy, every 4 cycles through Cycle 17, and then every 8 cycles thereafter.
4. PK assays should be performed at the mandatory Safety Follow-up Visit (if there is a ≥ Grade 3 irAE, an additional PK sample may be taken per the Schedule of Assessments).

Abbreviations: h, hour(s); irAE, immune-related adverse event; min, minutes; PK, pharmacokinetic(s).

3.3. Duration of Study

Total duration of study participation will vary by patient. Each study phase is further discussed in the main protocol in Section 3.3.1 though Section 3.3.4.

3.4. Dose-Limiting Toxicities

Dose-limiting toxicities will be assessed among evaluable patients after 28 days on study. An evaluable patient is defined as the patient who has received at least 80% of the dose and completed all safety assessments required during the first 28 days, or any patient who has experienced a DLT within 28 days. However, patients who received < 80% of the dose but experienced a DLT will also be part of the DLT assessment.

Among the 6 patients in the 200-mg Q3W cohort, if 2 or more patients experience DLT within 28 days, such starting dose will be considered as exceeding the maximum tolerated dose (MTD), and a lower dose, such as 150 mg Q3W, will be assessed subsequently in 3-6 patients. The first 6 patients will remain hospitalized for the first week of Cycle 1. Patients should have laboratory testing and a physical examination before determination of discharge by the physician. Based on clinical judgement, the hospital stay should be extended as required to manage patients. If the patient is discharged on Cycle 1 Day 7, the patient should additionally undergo the weekly assessment as specified per protocol. After the first week, hematology and serum chemistry (including liver function tests) will be performed weekly through the end of the 28-day DLT assessment period.

After the DLT assessment period, hematology and serum chemistry (including liver function tests) will be performed weekly for Cycle 2 and Cycle 3 and then at the beginning of subsequent cycles for Cycle 4 and beyond (data collected as specified in Appendix 5).

If the 200-mg Q3W dose passes the DLT assessment, the cohort at such dose level can be expanded to approximately 10 patients to further assess the safety, tolerability, PK and preliminary pharmacodynamic characteristics of tislelizumab. Up to 20 patients may be enrolled if more than 1 dose level is evaluated. In order to continuously monitor safety, when the cohort has been expanded to 10 patients and ≥ 33% of them have experienced DLT within 28 days, enrollment will be suspended, and an Independent Data Monitoring Committee (IDMC) meeting will be immediately held to discuss and determine whether such dose is safe.

3.5. Study Rationale

Before initiating a large Phase 3 study in Japan, this substudy in Japanese patients will provide additional safety information allowing the enrollment of patients into the larger Phase 3 study in Japan. Please see the main protocol, Section 3.4.1 and Section 3.4.2 for rationale on selection of tislelizumab in HCC and selection of dose.

4. MATERIALS AND METHODS

4.1. Selection of Study Population

4.2. Inclusion Criteria

To be eligible to participate in this study, a patient must meet all of the following criteria:

1. Is male or female Japanese patient, aged ≥20years on the day the patient voluntarily agrees to participate in the study (or the legal age of consent in the jurisdiction in which the study is taking place)
2. Has a histologically confirmed diagnosis of HCC
3. Has either Barcelona Clinic Liver Cancer (BCLC) Stage C disease, or BCLC Stage B disease that is not amenable to or has progressed after loco-regional therapy, and is not amenable to a curative treatment approach (see Forner et al, 2010)
4. For whom standard treatment for unresectable HCC is not available, not tolerated, or refused
5. Has ≥ 1 measurable lesion as defined per RECIST v1.1, provided that:
   - The target lesion(s) selected have not been previously treated with local therapy OR
   - The target lesion(s) selected that are within the field of prior local therapy have subsequently progressed as defined by RECIST v1.1
6. Has a Child-Pugh A classification for liver function assessed within 7 days before enrollment (Appendix 2 of the main protocol)
7. Has Eastern Cooperative Oncology Group (ECOG) Performance Status score ≤ 1
8. Has adequate organ function, as demonstrated by meeting all of the following clinical laboratory assessment criteria at Screening:
   - Absolute neutrophil count ≥ 1.5 × 10^9/L, platelets ≥ 75 × 10^9/L, and hemoglobin ≥ 85 g/L
     NOTE: Patients must not have required a transfusion of blood product and/or hematopoietic growth factors within the 14 days before sample collection
   - Estimated glomerular filtration rate (eGFR) > 30 mL/min/1.73m^2 by Chronic Kidney Disease Epidemiology Collaboration equation (Appendix 8 of the main protocol)
   - Serum albumin ≥ 29 g/L
   - Serum total bilirubin ≤ 51.3 µmol/L (3 mg/dl)
   - Aspartate aminotransferase (AST) and alanine aminotransferase (ALT) both ≤ 5 × ULN
9. If patient has hepatitis B virus (HBV) or hepatitis C virus (HCV) infection, meets the following criteria as applicable to the infection type:

   For patients with inactive/asymptomatic carrier, chronic, or active HBV:

- Has HBV deoxyribonucleic acid (DNA) < 500 IU/mL (or 2500 copies/mL) at Screening
  NOTE: Patients with detectable hepatitis B surface antigen (HBsAg) or detectable HBV DNA should be managed per treatment guidelines. Patients receiving antivirals at Screening should have been treated for > 2 weeks prior to enrollment and should continue treatment for 6 months after study drug treatment discontinues.

For patients with HCV:

- Infection is evidenced by detectable HCV ribonucleic acid (RNA)

10. If a female of childbearing potential (ie, physiologically capable of becoming pregnant), agrees to practice highly effective methods of birth control (Appendix 6 of the main protocol) for the duration of the study and for > 120 days after the last dose of tislelizumab and have a negative urine or serum pregnancy test within 7 days of the first study drug administration.
11. If a non-sterile male, agrees to practice highly effective methods of birth control for the duration of the study and for > 120 days after the last dose of tislelizumab.

4.3. Exclusion Criteria

To be eligible to participate in this study, a patient cannot meet any of the following exclusion criteria:

1. Has known fibrolamellar HCC, sarcomatoid HCC, or mixed cholangiocarcinoma and HCC histology
2. Has tumor thrombus involving main trunk of portal vein or inferior vena cava
3. Has received within 28 days before enrollment loco-regional therapy to the liver (ie, TACE, transcatheter embolization, hepatic arterial infusion, radiation, radioembolization, or ablation)
4. Has received within 28 days before enrollment any prior immunotherapy (eg, interleukin, interferon, thymoxin, et cetera) **or within 14 days** any Chinese herbal medicine or patent medicine used to control cancer (Appendix 11)
5. Has, at Screening, and/or has any prior history of ≥ Grade 2 hepatic encephalopathy (Appendix 2 of the main protocol)
6. Has, at Screening, pericardial effusion, uncontrollable pleural effusion, or clinically significant ascites defined as meeting either of (a) detectable ascites on Screening physical examination OR (b) has at Screening, ascites requiring paracentesis
7. Has a history of severe hypersensitivity reaction to other monoclonal antibodies
8. Has, at Screening, or has had within 6 months before enrollment any clinical evidence of portal hypertension with bleeding esophageal or gastric varices
9. Patients with toxicities which have not recovered to baseline or stabilized as a result of prior anticancer therapy, except alopecia

10. Has, at Screening, or has had within 6 months before enrollment, any bleeding or thrombotic disorder or any prescribed anticoagulant requiring therapeutic international normalized ratio monitoring (eg, warfarin or similar agents)
11. Has, at Screening, or has had within the 2 years before enrollment, any active malignancy, with the exception of the HCC under investigation in this trial and any locally recurring cancer that has been treated curatively (eg, resected basal or squamous cell skin cancer, superficial bladder cancer, carcinoma in situ of the cervix or breast)
12. Has, at Screening, any known central nervous system metastasis and/or leptomeningeal disease
13. Has, at Screening, any active immune deficiency or autoimmune disease and/or has a history of any immune deficiency or autoimmune disease that may relapse (Appendix 4 of the main protocol)

    NOTE: Patients with the following diseases are not excluded:

    - Type 1 diabetes
    - Hypothyroidism (provided it is managed with hormone replacement therapy only)
    - Controlled celiac disease
    - Skin diseases not requiring systemic treatment (eg, vitiligo, psoriasis, alopecia)
    - Any other disease that is not expected to recur in the absence of external triggering factors
14. Has any condition that has required systemic treatment with either corticosteroids (> 10 mg daily of prednisone or equivalent) or other immunosuppressive medication within 14 days before enrollment

    NOTE: Patients who are currently or have previously been on any of the following steroid regimens are not excluded:

    - Adrenal replacement steroid (dose < 10 mg daily of prednisone or equivalent) in the absence of active autoimmune disease
    - Topical, ocular, intra-articular, intranasal, or inhalational corticosteroid with minimal systemic absorption
    - Short course of corticosteroid prescribed prophylactically (eg, for contrast dye allergy) or for the treatment of a non-autoimmune condition (eg, delayed-type hypersensitivity reaction caused by contact allergen)
15. Has any history of interstitial lung disease or non-infectious pneumonitis, unless induced by radiation therapy
16. Has, at Screening, any severe chronic or active infection (excluding viral hepatitis) requiring systemic antibacterial, antifungal, or antiviral therapy (eg, tuberculosis)
17. Has Screening ECGs with QT interval corrected for heart rate (QTc) (corrected by Fridericia's method) > 450 msec

NOTE: If any patient has QTc > 450 msec on initial ECG, a follow-up ECG will be performed to confirm result

18. Has any of the following cardiovascular risk factors:
    - Cardiac chest pain, defined as moderate pain that limits instrumental activities of daily living (ADL), within 28 days before enrollment
    - Symptomatic pulmonary embolism within 28 days before enrollment
    - Any history of acute myocardial infarction within 6 months before enrollment
    - Any history of heart failure meeting New York Heart Association Classification III or IV (Appendix 7 of the main protocol) within 6 months before enrollment
    - Any event of ventricular arrhythmia > Grade 2 in severity within 6 months before enrollment
    - Any history of cerebrovascular accident or transient ischemic attack within 6 months before enrollment
19. Has a known history of human immunodeficiency virus
20. Has any underlying medical condition that, in the investigator's opinion, will make the administration of study treatment hazardous or potentially obscure the interpretation of AEs/toxicities
21. Has undergone prior allogeneic stem cell transplantation or organ transplantation
22. Has been administered a live vaccine within 4 weeks before enrollment
    NOTE: Seasonal vaccines for influenza are generally inactivated vaccines and are allowed. Intranasal vaccines are live vaccines, and are not allowed.
23. Has undergone any major surgical procedure within 28 days before enrollment
24. Female patients who are pregnant or nursing

    Note : Female patients who suspend nursing are allowed. If the patient wishes to resume nursing after completion of treatment, time from the completion of study drug treatment until nursing resumption would be 90 days (>5 half-lives of tislelizumab).

5. STUDY TREATMENT

5.1. Formulation, Packaging, Handling, and Storage

Please refer to the Section 5.1 of the main protocol for information on the formulation, packaging, handling and storage of tislelizumab.

5.2. Dosage, Administration, and Compliance

Patients will be treated with 200 mg intravenous (IV) tislelizumab Q3W until intolerable toxicity, withdrawal of informed consent, or the time point at which, in the opinion of the investigator, the patient is no longer benefiting from study therapy. All patients will be monitored continuously for AEs. Treatment modifications (eg, dose delay, reduction,

interruption, or discontinuation) will be based on specific laboratory assessment and AE criteria, as described in Section 5.5 of the main protocol.

5.3. Handling of Overdose

Please refer to Section 5.3 of the main protocol for detailed information.

5.4. Investigational Medicinal Product Accountability

Please refer to Section 5.4 of the main protocol for detailed information.

5.5. Disposal and Destruction

Please refer to Section 5.6 of the main protocol for detailed information.

6. PRIOR AND CONCOMITANT THERAPY

Please refer to Section 6 of the main protocol for detailed information.

7. STUDY ASSESSMENTS AND PROCEDURES

Study assessments and procedures will be similar to those in the main protocol, Section 7 (Sections 7.1.3 through Section 7.13), with the exception that patients will not be randomized for the substudy and there is no second treatment arm. In addition, PK assessments have been increased in this substudy for thorough characterization of PK after single dose and at steady state in Japanese patients and are provided in further detail in Section 9.7 of this substudy protocol. Patients will be closely monitored for safety and tolerability throughout the study and the first 6 patients will be hospitalized during Cycle 1 of treatment. Patients should have laboratory testing and physical examination before determination of discharge by the physician. Based on clinical judgement, hospital stays should be extended as required to manage patients. If a patient is discharged on Cycle 1 Day 7, the patient should additionally undergo the weekly assessment as specified per protocol. All assessments must be performed and documented in the medical record and electronic case report form (eCRF) for each patient.

Tumor response will be assessed by investigators based on RECIST v1.1. For immune therapies such as tislelizumab, pseudo-progression may occur due to immune-cell infiltration and other mechanisms leading to apparent increase of existing tumor masses or appearance of new tumor lesions. Thus, for progressive disease (PD) suspected by the investigator as pseudo-progression, the following criteria must be met in order to treat patients continuously until PD is confirmed by repeated imaging performed no more than 6 to 8 weeks beyond the initial diagnosis of radiographic PD:

- Absence of clinical symptoms and signs of disease progression (including worsening laboratory values)
- ECOG PS ≤ 1

- Absence of rapid progression of disease or progression at a critical anatomical site (eg, progression of a spinal lesion with impending cord compression) or that necessitates urgent alternative medical intervention

8. SAFETY MONITORING AND REPORTING

Safety monitoring and reporting procedures will follow those in the main protocol (Section 8). An addition to the safety assessment will be digital pulse oximetry. Another addition to this substudy is the definition of DLTs. All toxicities or AEs will be graded according to the NCI-CTCAE Version 4.03. The occurrence of any of the following toxicities within 28 days after the first dose of tislelizumab if judged by the investigator as related to tislelizumab will be considered a DLT. The first 6 patients will remain hospitalized for the first week of Cycle 1. After the first week, hematology and serum chemistry (including liver function tests) will be performed weekly through the end of the 28-day DLT assessment period. After the DLT assessment period, hematology and serum chemistry (including liver function tests) will be performed weekly for Cycle 2 and Cycle 3 and then at the beginning of subsequent cycles from Cycle 4 and beyond (data collected as specified in Appendix 5).

Hematologic:

1. Grade 4 neutropenia lasting >7 days
2. Grade 3 febrile neutropenia (defined as absolute neutrophil count [ANC] <1000/mm^3 with a single temperature of 38.3^{o}C or a sustained temperature of 38°C for >1 hour).
3. Grade 3 neutropenia with infection.
4. Grade 3 thrombocytopenia with bleeding or requiring transfusion.
5. Grade 4 thrombocytopenia with or without transfusion.
6. Grade 4 anemia (life-threatening) or Grade 3 anemia requiring transfusion.

Non-hematologic:

1. Grade 4 or above toxicity
2. Grade 3 toxicity lasting more than 7 days despite optimal supportive care

Note: The following AEs will not be considered as DLTs:

- Grade 3 endocrinopathy that is adequately controlled by hormonal replacement
- Grade 3 AE of tumor flare (defined as local pain, irritation, or rash localized at sites of known or suspected tumors)
- Grade 3 rash
- Grade 3 to Grade 4 laboratory abnormalities that are not associated with clinical sequelae (eg, lactate dehydrogenase [LDH])

In addition, AEs leading to temporary or permanent discontinuation of tislelizumab dosing, with the exception of infusion-related reactions should be considered a DLT and any clinically

important or persistent toxicities (including infusion-related reactions) that are not included above may also be considered a DLT following review by the IDMC.

Patients who received < 80%of the assigned dose of tislelizumab (eg, because the infusion had to be discontinued due to an infusion reaction) and did not experience a DLT will not be taken into account in the assessment of the overall DLT rate for the particular dose level.

Independent Data Monitoring Committee

An IDMC (described in Section 10.2 of the main protocol) will evaluate and confirm the Phase 3 dose based on the safety and tolerability of tislelizumab, and decide whether or not to add unscheduled dose levels for trial. In order to confirm the pivotal Phase 3 dose, an IDMC will be held within 6 weeks after the 6th patient is treated. If ≥ 33% have experienced DLT within 28 days after the first dose of tislelizumab, enrollment will be suspended, and an IDMC will determine whether such a dose is safe or if a lower dose level should be tested. If < 33% have experienced DLT, the cohort will be expanded to 10 patients in total to provide more safety experience on treatment.

Safety review will then subsequently be followed as per the main protocol. The IDMC may recommend modifications to the study, including termination due to safety and/or efficacy concerns.

9. STATISTICAL METHODS AND ANALYSIS PLAN

As described in the main protocol, Section 9, the statistical analyses will be performed by the sponsor or designee after the data collection for the primary efficacy and safety analyses are completed and the database is locked and released. Data will be listed and summarized using SAS® Version 9.3 or higher (SAS Institute, Inc., Cary, North Carolina) per sponsor agreed reporting standards, where applicable. Details of the statistical analyses will be included in a separate Statistical Analysis Plan.

9.1. Analysis Sets

- DLT Evaluable Analysis Set: Includes all patients who have received at least 80% of the dose and completed all safety assessments required in Cycle 1, or any patient who has experienced DLT in Cycle 1
- PK Analysis Set: Includes all patients who receive at least 1 dose of tislelizumab per the protocol, for whom any postdose PK data are available
- Safety Analysis Set: Includes all patients who received > 1 dose of study drug and is the primary analysis set used for all safety analyses

9.2. Patient Disposition

Please refer to Section 9.2 of the main protocol for detailed information.

9.3. Demographic and Other Baseline Characteristics

Please refer to Section 9.3 of the main protocol for detailed information.

9.4. Prior and Concomitant Therapies

Please refer to Section 9.4 of the main protocol for detailed information.

9.5. Analyses

Descriptive statistics will be used to summarize the demographic, disease characteristic, efficacy and safety data. No statistical hypotheses are planned in this exploratory study.

Safety analyses will be performed with the Safety analysis set and PK analyses with the PK analysis set. For other parameters, all evaluable data will be included in the summaries.

9.5.1. Primary Analyses

Tislelizumab safety and tolerability: The safety of tislelizumab will be assessed throughout the study by monitoring AEs and serious SAEs per NCI-CTCAE v4.03, relevant physical examination, ECGs and laboratory assessments as needed.

The recommended dose for the Phase 3 for tislelizumab will be determined based on safety, tolerability, PK, and other available data.

9.5.2. Secondary Analyses

Efficacy evaluations per RECIST v1.1 (ie, ORR, PFS and DOR) will be summarized to explore the preliminary anticancer activities in Japanese patients.

The ORR is defined as the proportion of patients who had confirmed complete response (CR) or partial response (PR) assessed by the investigator using RECIST v1.1.

The DOR is defined as the time from the first determination of a confirmed objective response by investigator according to RECIST v1.1 until the first documentation of progression or death, whichever comes first.

Progression-Free Survival is defined as the time from the date of first dose of study drug to the date of first documentation of disease progression assessed by investigator using RECIST v1.1 or death, whichever occurs first.

The ORR and its 95% confidence interval (CI) will be summarized in the Safety analysis set. The PFS and DOR will be estimated using the Kaplan-Meier method. Waterfall plot of maximum tumor shrinkage per patient will be presented.

Immunogenic responses to tislelizumab will be assessed by monitoring the incidence of ADAs.

9.5.3. Exploratory Analyses

Assessments of the correlations between drug exposure and response (efficacy and safety endpoints) will be made. Results of such exploratory analysis may be reported separately from the clinical study report (CSR).

9.6. Safety Analyses

As described in the main protocol (Section 9.6), safety will be assessed by monitoring and recording of all AEs graded by NCI-CTCAE v4.03. Laboratory values (eg, hematology, clinical chemistry), vital signs, ECGs, and physical examinations will also be used in determining safety.

Descriptive statistics will be used to analyze all safety data in the Safety analysis set. Substudy Protocol Section 8 includes DLT definitions as well as specific information on the role of the IDMC in the assessment of DLTs and the confirmation of the Phase 3 dose.

9.7. Pharmacokinetic Analyses

Pharmacokinetic samples will be collected in this study as outlined in Appendix 13 Table 2. The PK analysis will use noncompartmental methods to calculate the following PK parameters, as appropriate and allowed by data: including but not limited to area under the concentration-time curve from Day 0 to Day 21 (area under the concentration-time curve from Day 0 to Day 21 [$AUC_{0\text{-}21d}$]), C_{max}, time to maximum plasma concentration [T_{max}], trough serum concentration [C_{trough}], elimination half-life ($t_{1/2}$), clearance (CL) and volume of distribution (Vd). Mean serum tislelizumab concentration data and PK parameters will be tabulated and summarized by visit/cycle at which these data are available. Concentrations of tislelizumab will be summarized descriptively. Descriptive statistics will include means, medians, ranges, and standard deviations, as appropriate. Additional PK analyses may be conducted as appropriate.

Details concerning handling of PK serum samples, including labeling and shipping instructions will be provided in the Lab Manual. Samples will be shipped to the central laboratory where all samples will be analyzed for serum tislelizumab concentrations using a validated method. The actual time each sample was collected will be captured to the nearest minute in the electronic CRF (eCRF) and recorded in the database.

9.8. Immunogenicity Analyses

As described in the main protocol (Section 9.8), the immunogenicity results will be summarized using descriptive statistics by the number and percentage of patients who develop detectable ADAs. The incidences of positive ADAs and neutralizing ADAs will be reported for evaluable patients. The effect of immunogenicity on PK, efficacy, and safety may be evaluated if data allow.

10. STUDY COMMITTEES AND COMMUNICATION

Please refer to Section 10 of the main protocol for detailed information.

11. SOURCE DOCUMENTS AND ACCESS TO SOURCE DATA/DOCUMENTS

Please refer to Section 11 of the main protocol for detailed information.

12. QUALITY ASSURANCE AND QUALITY CONTROL

Please refer to Section 12 of the main protocol for detailed information.

13. ETHICS/PROTECTION OF HUMAN SUBJECTS

Please refer to Section 13 of the main protocol for detailed information.

14. DATA HANDLING AND RECORD KEEPING

Please refer to Section 15 of the main protocol for detailed information.

APPENDIX 14. REGIONAL PRODUCT LABELS FOR SORAFENIB

Regional product labels for sorafenib are provided in the following links:

US FDA Label:

http://labeling.bayerhealthcare.com/html/products/pi/Nexavar_PI.pdf

UK Label:

http://www.ema.europa.eu/docs/en_GB/document_library/EPAR_-_Product_Information/human/000690/WC500027704.pdf

France SmPC:

https://www.ema.europa.eu/en/documents/product-information/nexavar-epar-product-information_fr.pdf

Japan Label:

https://protect-us.mimecast.com/s/cRARCJ6A6oT8E1GBSVGYmm?domain=info.pmda.go.jp/
