# Supplementary material for: Case Report: Clinical Responses to Tislelizumab as a First-Line Therapy for Primary Hepatocellular Carcinoma With B-Cell Indolent Lymphoma
Source: Front Immunol. 2021 Mar 31;12:634559. doi: 10.3389/fimmu.2021.634559 (PMC8044442; doi:10.3389/fimmu.2021.634559)
Supplement: Supplementary file 1 [file Presentation_1.zip › 634559_SupMaterial/Figure Legends.DOCX]

**Figure Legends**

**Figure S1. The histopathology of the liver tumor in the left lateral lobe in Dongyang People's Hospital.**

The pathology of lesion in the left lateral segment liver showed hepatocellular carcinoma as well as chronic hepatitis with cirrhosis.

**Figure S2. Hematological detections of the patient in recent follow-up.**

The figure S2B-S2D shows that from Jun 2020, the counts of lymphocytes ascended gradually, and we in order to find the main increasing subgroup, we complete the detection of lymphocytes subsets (figure S2A) which indicates the high level of B lymphocytes.
