# Supplementary figures and images for: Case Report: Clinical Responses to Tislelizumab as a First-Line Therapy for Primary Hepatocellular Carcinoma With B-Cell Indolent Lymphoma
Source: Front Immunol. 2021 Mar 31;12:634559. doi: 10.3389/fimmu.2021.634559 (PMC8044442; doi:10.3389/fimmu.2021.634559)

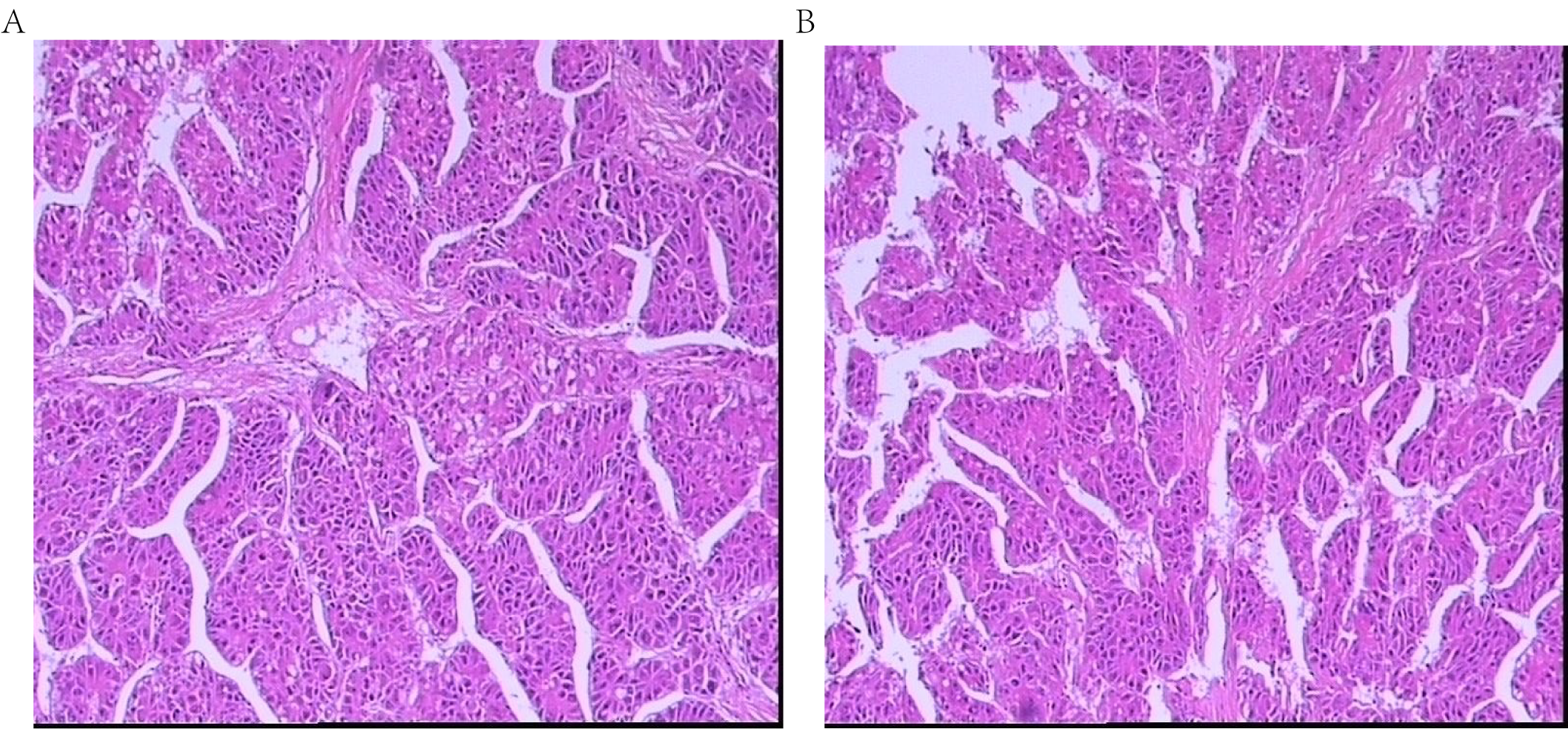

Supplement: Supplementary file 1 [file Presentation_1.zip › 634559_SupMaterial/Image 1.TIF]

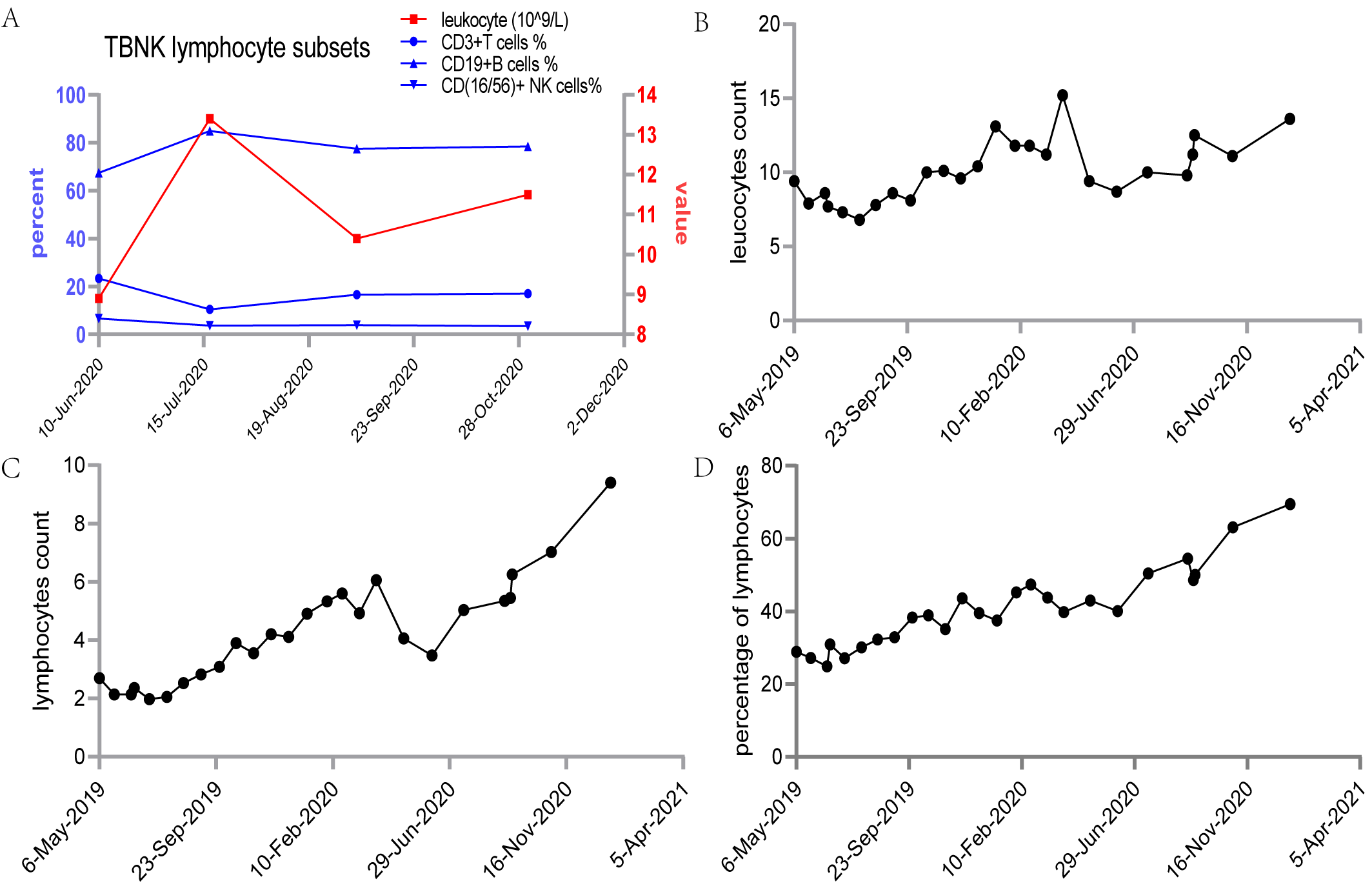

Supplement: Supplementary file 1 [file Presentation_1.zip › 634559_SupMaterial/Image 2.TIF]
